# Cryo‐EM reveals mechanisms of angiotensin I‐converting enzyme allostery and dimerization

**DOI:** 10.15252/embj.2021110550

**Published:** 2022-07-12

**Authors:** Lizelle Lubbe, Bryan Trevor Sewell, Jeremy D Woodward, Edward D Sturrock

**Affiliations:** ^1^ Department of Integrative Biomedical Sciences, Institute of Infectious Disease and Molecular Medicine University of Cape Town Cape Town South Africa; ^2^ Electron Microscope Unit University of Cape Town Cape Town South Africa

**Keywords:** angiotensin I‐converting enzyme, cryo‐electron microscopy, glycoprotein, homodimerization, zinc metalloprotease, Cardiovascular System, Structural Biology

## Abstract

Hypertension (high blood pressure) is a major risk factor for cardiovascular disease, which is the leading cause of death worldwide. The somatic isoform of angiotensin I‐converting enzyme (sACE) plays a critical role in blood pressure regulation, and ACE inhibitors are thus widely used to treat hypertension and cardiovascular disease. Our current understanding of sACE structure, dynamics, function, and inhibition has been limited because truncated, minimally glycosylated forms of sACE are typically used for X‐ray crystallography and molecular dynamics simulations. Here, we report the first cryo‐EM structures of full‐length, glycosylated, soluble sACE (sACE^S1211^). Both monomeric and dimeric forms of the highly flexible apo enzyme were reconstructed from a single dataset. The N‐ and C‐terminal domains of monomeric sACE^S1211^ were resolved at 3.7 and 4.1 Å, respectively, while the interacting N‐terminal domains responsible for dimer formation were resolved at 3.8 Å. Mechanisms are proposed for intradomain hinging, cooperativity, and homodimerization. Furthermore, the observation that both domains were in the open conformation has implications for the design of sACE modulators.

## Graphical abstract

The highly dynamic nature of the somatic isoform of the blood pressure regulator, sACE, as revealed by CryoEM provides new mechanistic insights.

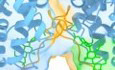

## Introduction

The zinc metalloprotease angiotensin I‐converting enzyme (ACE;EC 3.4.15.1) is well‐known for its crucial role in blood pressure regulation and fluid homeostasis via the renin–angiotensin–aldosterone system (RAAS), where it catalyzes the hydrolysis of angiotensin I (Ang I) to the vasopressor angiotensin II (Ang II; Ehlers & Riordan, 1989). Hypertension is a major risk factor for heart disease and stroke, which are the two leading causes of death worldwide (https://www.who.int/news‐room/fact‐sheets/detail/the‐top‐10‐causes‐of‐death). The centrality of ACE in the RAAS led to the early development of ACE inhibitors for the treatment of hypertension (Ondetti *et al*, 1977). However, despite their widespread use, the function of ACE and the effects of its inhibition at the molecular level remain poorly understood.

**Figure 1:**
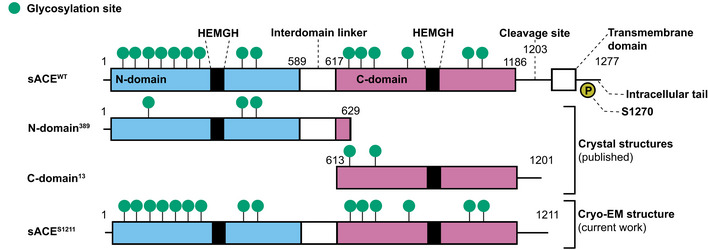
sACE protein sequence schematic The differences in glycosylation and construct length between wild‐type (WT) sACE and the constructs used for X‐ray crystallography and cryo‐EM are shown. N‐domain^389^ is only glycosylated at sites 3 (N45), 8 (N416), and 9 (N480) while C‐domain^13^ is only glycosylated at sites 1 (N648) and 3 (N685). The yellow circle indicates phosphorylation at the intracellular S1270. The zinc‐binding HEMGH motif is indicated for each domain. The R1203/S1204 secretase cleavage site for proteolytic release of soluble sACE from the cell membrane is indicated.

The somatic isoform of ACE (sACE) is a 1,277‐amino acid type‐I integral membrane protein with a short C‐terminal cytoplasmic tail (Fig 1). A soluble form of sACE is released following ectodomain shedding (Ehlers *et al*, 1996, 2012; English *et al*, 2012). Each sACE molecule is composed of two homologous domains (N‐ and C‐domains) separated by a short linker region and each domain harbors a catalytic site with a HEMGH zinc‐binding motif (Wei, 1991). sACE is related to the carboxypeptidase ACE2 which, in addition to its role in the RAAS (Turner & Nalivaeva, 2022), acts as the functional receptor for the severe acute respiratory syndrome (SARS) coronavirus (Li *et al*, 2003) and SARS coronavirus 2 (SARS‐CoV‐2; Hoffmann *et al*, 2020). However, ACE2 only has a single peptidase domain and differs from sACE in terms of ligand binding specificity (Donoghue *et al*, 2000; Rice *et al*, 2004).

The two sACE domains are highly homologous (60% overall sequence identity and 90% active site identity) but display distinct differences in substrate and inhibitor binding specificities. A wide range of substrates can be cleaved by sACE through endo‐ or exopeptidase activity (Bernstein *et al*, 2013). Despite the increasing awareness of the importance of sACE in different physiological processes (Bernstein *et al*, 2013; Cao *et al*, 2020, 2022; Le *et al*, 2021), the molecular mechanisms of substrate binding/hydrolysis remain poorly understood, partly due to limitations in previous structure–function relationship studies. The N‐ and C‐domains have 9 and 6 potential *N*‐linked glycosylation sites, respectively, which are important for folding, processing, and thermal stability (Gordon *et al*, 2003; O'Neill *et al*, 2008; Anthony *et al*, 2010) but their flexibility, and that of the interdomain linker, has previously hampered crystal structure determination.

Thus far, crystal structures have only been solved for truncated, single‐domain, minimally glycosylated forms of sACE. Crystal structures (Cozier *et al*, 2020b), normal mode analysis (NMA; Watermeyer *et al*, 2006), and molecular dynamics (MD) simulations (Lubbe *et al*, 2016; Vy *et al*, 2020) have suggested that each domain transitions from an open to a closed conformation upon ligand binding. There appears to be a form of allosteric regulation within each domain since mutations at distal sites drastically alter inhibitor binding (Lubbe *et al*, 2016; Sturrock *et al*, 2019; Cozier *et al*, 2020a). However, sACE is a two‐domain protein *in vivo*, and the existing crystal structures naturally cannot offer insights into its dynamics. The relative domain orientation is also unknown and is important to determine, since interdomain cooperativity occurs upon the binding of certain substrates or inhibitors (Ehlers & Riordan, 1991; Binevski *et al*, 2003; Skirgello *et al*, 2005; Masuyer *et al*, 2015; Larmuth *et al*, 2016). In addition, N‐domain patient mutations (Danilov *et al*, 2011) or shear stress (via increased blood velocity) (Barauna *et al*, 2011) altered proteolytic cleavage/shedding of sACE at the C‐terminal side of the C‐domain (Ehlers *et al*, 1996).

Apart from intra‐ and interdomain interactions, both soluble and membrane‐bound sACE are also capable of homodimerization (Kost *et al*, 2000, 2003; Kohlstedt *et al*, 2004, 2006; Gordon *et al*, 2010; Abrie *et al*, 2018). sACE also has noncatalytic roles where it initiates inhibitor‐ and substrate‐induced dimerization and intracellular signaling (Fleming, 2006; Sun *et al*, 2010). This is noteworthy since some beneficial effects of ACE inhibitors cannot merely be ascribed to a reduction in Ang II formation (Lee *et al*, 1999; Ehlers *et al*, 2013). Currently, the mechanism of dimerization is unclear, and is important to elucidate since dimerization altered cyclooxygenase‐2 (COX‐2) and sACE expression (Kohlstedt *et al*, 2004; Sun *et al*, 2010), and is likely linked to shedding.

As a first step towards uncovering these mechanisms of shapeshifting within and between sACE molecules, in the present study, we aimed to (i) determine the apo structure of full‐length soluble sACE; (ii) gain insight into intradomain hinging, interdomain cooperativity, and allostery; and (iii) determine the mechanism of sACE homodimerization. While protein and glycan flexibility can hinder structure solution via X‐ray crystallography, this is not the case for cryo‐electron microscopy (cryo‐EM; Orlova & Saibil, 2011). Recent advances in cryo‐EM data collection and image processing algorithms (Bai *et al*, 2015; Egelman, 2016) have made it possible to study sACE, which is relatively small and highly flexible, in its native glycosylated state under close‐to‐physiological conditions.

Here, we report the first cryo‐EM reconstructions of both monomeric and dimeric soluble apo sACE obtained from a single dataset at resolutions of 3.6–4.1 Å. Both domains of the apoprotein were in an open conformation. Potential mechanisms for intra‐ and interdomain cooperativity were revealed experimentally using 3D variability analysis (3DVA), which describes the continuous structural flexibility of proteins imaged by cryo‐EM, and confirmed computationally using NMA. Bending, breathing, and swinging motions of the domains were observed for the first time. Cavity detection with analysis of their motion correlation allowed the identification of promising allosteric sites. We conclusively show that homodimerization under basal conditions occurs via N‐domain protein–protein and glycan‐glycan interactions. These interactions are proximal to an allosteric site, dramatically alter C‐domain dynamics, and induce rotamer changes in key N‐domain catalytic residues to potentially reduce sACE activity. This study represents an important milestone in understanding sACE and heralds a new era of research into its structure–function relationship, which will facilitate improved ACE inhibitor design.

## Results

### Soluble sACE^S1211^ dimerizes *in vitro*

**Figure 2:**
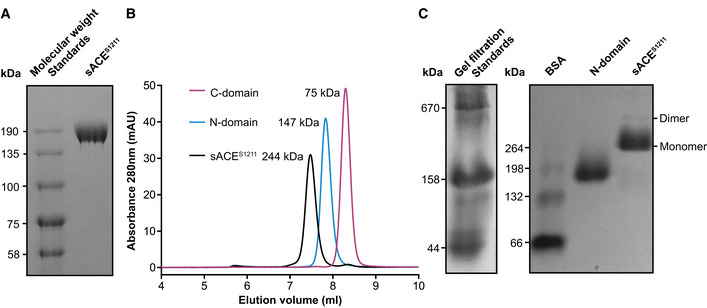
Biochemical characterization of purified sACE^S1211^ ASDS–PAGE analysis of purified sACE^S1211^ showed a single band of ~170 kDa and thus indicates extensive *N*‐glycosylation (protein‐only molecular weight is 138 kDa).BSEC–HPLC analysis of the respective samples of purified sACE^S1211^, truncated N‐domain, or truncated C‐domain showed a single symmetric peak for each protein with calibrated molecular weights of 244, 147, and 75 kDa, respectively. The sACE^S1211^ sample did not show any peaks corresponding to single‐domain or homodimer molecular weights.CNative PAGE analysis showed a major diffuse band of ~260 kDa and a minor band of ~500 kDa for purified sACE^S1211^, representing monomeric and dimeric forms of the protein. Commercial gel filtration standards and bovine serum albumin (BSA) were used to estimate the molecular weight. Purified truncated N‐domain was used as an additional molecular weight marker for sACE^S1211^ and showed a single diffuse band of ~160 kDa. Source data are available online for this figure.

An engineered form of sACE (sACE^S1211^) was recombinantly expressed in CHO‐K1 cells and purified to homogeneity. This construct encodes L1‐S1211 of sACE (138 kDa protein‐only molecular weight) and thus includes the two ectodomains, an interdomain linker, and a part of the juxtamembrane stalk, but lacks the transmembrane (TM) domain and intracellular tail (Fig 1). It does not have N→Q mutation of glycosylation sites, which are used for X‐ray crystallography (Fig 1), and since glycosylation was not inhibited during expression or enzymatically removed, the purified protein is heavily *N*‐glycosylated. This is evident from the single band of ~170 kDa that was observed by SDS–PAGE (Fig 2A).

**Figure EV1:**
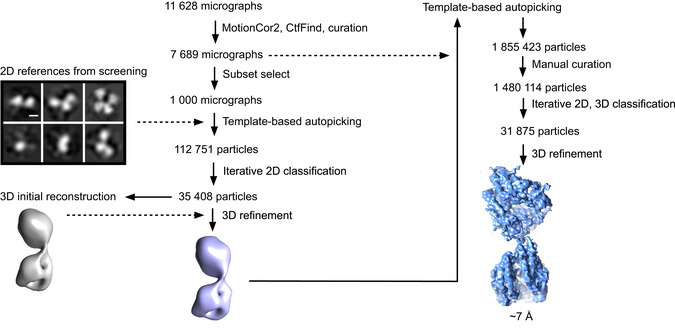
Schematic of initial image processing from all micrographs in RELION using 2D references obtained during sample screening A 3D reconstruction of the dimer was not obtained while the monomer yielded a final ~7 Å reconstruction. Scale bar: 50 Å.

Our purified sACE^S1211^ had a specific activity of 49,094 ± 5,464 mU/mg in the presence of zinc and chloride cofactors, which is comparable to previous results (Lanzillo *et al*, 1980). Analytical size‐exclusion chromatography (SEC; Fig 2B) showed a single symmetrical peak for sACE^S1211^ at a molecular weight of 244 kDa (see Appendix Fig S1A for the trace of the gel filtration standards and Appendix Fig S1B for the calibration curve), suggesting that sACE^S1211^ was mostly monomeric in solution, and confirming its integrity as no peaks corresponding to the individual domains were detected (Fig 2B). A fraction of dimer particles was observed during preliminary cryo‐EM analysis (Fig EV1), the presence of which was confirmed as a faint band of ~500 kDa by native PAGE (Fig 2C). Our attempts to obtain separate fractions of apo monomeric and dimeric sACE^S1211^ by SEC were unsuccessful, potentially due to a monomer‐dimer equilibrium in solution. This sample was therefore directly used for cryo‐EM grid preparation.

### Cryo‐EM image processing reveals sACE^S1211^ conformational heterogeneity

**Figure EV2:**
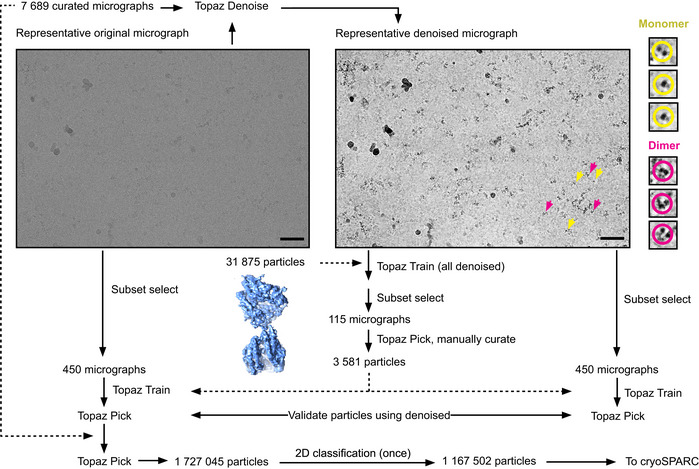
Schematic depiction of Topaz processing for sACE^S1211^ Micrographs were denoised and training of a picking model for sACE^S1211^ done in parallel on noisy micrographs and their denoised counterparts. The 31,875 monomer particles, which refined to ~7 Å resolution during initial processing, were used as positive labels. Coordinates obtained from the denoised micrographs were used for manual validation and optimization of picking parameters. After validating picking from noisy micrographs by comparison with the corresponding denoised coordinates, the model trained on noisy data was used for picking from all micrographs. Solid arrows indicate sequential jobs where the results from one step fed directly into the next. Dashed arrows indicate where results from earlier steps were used as additional input. Scale bar: 50 nm.

An initial 7 Å cryo‐EM reconstruction of the monomer was obtained by: (i) template‐based autopicking of a subset of micrographs using 2D class averages obtained during screening, (ii) 3D reconstruction, (iii) a second round of template‐based autopicking from all 7,689 micrographs, and (iv) iterative 2D and 3D classifications in RELION (Scheres, 2012; Zivanov *et al*, 2018; see Fig EV1 for details). Particles from this reconstruction were used as positive labels to train a positive‐unlabelled neural network particle picking model using Topaz (Bepler *et al*, 2019; Fig EV2). The model was optimized by manual curation to pick both monomer and dimer particles. Although micrograph denoising with Topaz‐Denoise (Bepler *et al*, 2020) aided visualization, the globular nature of the sACE^S1211^ domains still made it difficult to distinguish them from noise in micrographs collected at minimal defocus. Thus, optimization of particle picking was challenging, especially for the monomer top view, which was essentially a single circular spot on the micrograph.

**Figure EV3:**
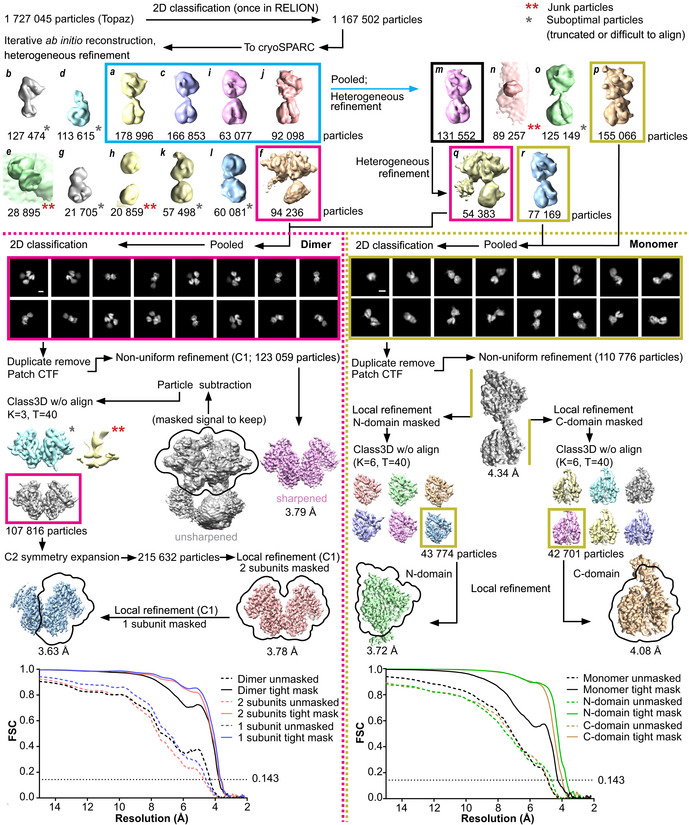
Schematic depiction of image processing performed to obtain monomeric and dimeric sACE^S1211^ reconstructions Class3D without align and particle subtraction were done in RELION with all other stages performed in cryoSPARC. 3D classes are labeled *a‐r*. Scale bar: 50 Å.

Following a single round of 2D classification, the stack of ~1.2 million Topaz‐picked particles was used directly for 3D reconstruction in cryoSPARC (Punjani *et al*, 2017). *Ab initio* reconstruction and heterogeneous refinement revealed substantial heterogeneity. We observed 3D classes of seemingly truncated single‐domain forms (classes *b*, *d*, and *g* Fig EV3), monomer classes with no clefts but with variations in the degree of separation between the two domains (classes *k*, *l*, and *o*), and monomer classes with clear clefts in both domains (classes *a*, *c*, *i*, *j*, *m*, *p*, and *r*). Although the protein seemed mostly monomeric in solution, the dimer was detected in both 2D and 3D, and showed clear density for three of the domains (classes *f* and *q*). Correlation artifacts likely affected sACE^S1211^ separation and caused particles from distinct conformations to pool together, for example, a single monomer 3D class (class *m*) with no extra density separated into ~40% dimer (class *q*) and ~60% monomer (class *r*) upon further heterogeneous refinement.

**Figure 3:**
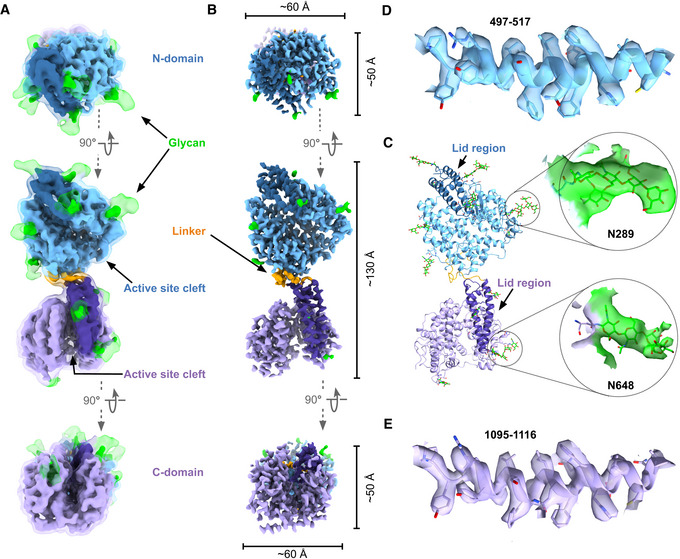
Cryo‐EM structure of monomeric sACE^S1211^ A, B(A) The consensus 4.3 Å full‐length soluble monomer map from nonuniform refinement resembled an hourglass shape with close association between the two domains. Glycan density was observed at a low volume threshold of the unsharpened map (transparent surface). (B). The higher resolution maps after local refinement showed a wide active site cleft for each domain. (A, B) Orthogonal views of the cryo‐EM density show the disparity in particle dimensions.CFull‐length soluble monomer model with fitting of a representative glycan shown for each domain.D, EModel fit to map illustrated for the equivalent representative region of the N‐ and C‐domain.

**Figure 4:**
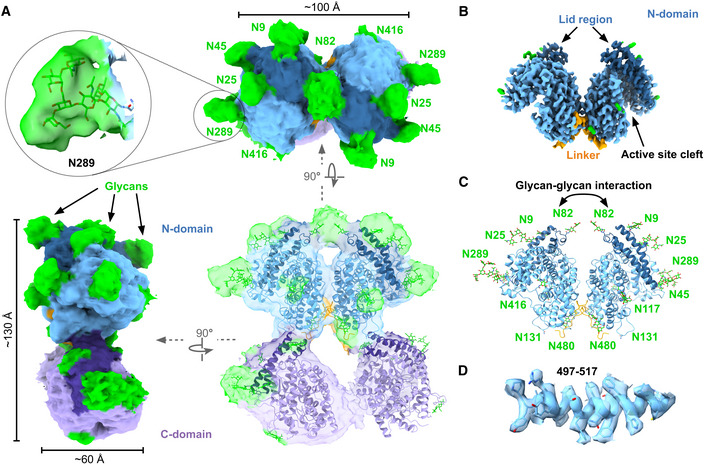
Cryo‐EM structure of dimeric sACE^S1211^ AThe consensus 3.8 Å full‐length soluble dimer map from nonuniform refinement showed close association between two N‐domains with glycan‐glycan interactions at N82. Fitting of a representative glycan at site N289 is shown. The 4‐domain frontal view and the 2‐domain top view at a low contour level illustrate the shield of glycans surrounding the dimer and the disordered C‐domain density. Two monomer C‐domain models were docked to illustrate the approximate full‐length soluble dimer structure.B, CThe local refinement map of the interacting N‐domains was resolved to a higher resolution, and the model showed wide active site clefts, and contact between the interdomain linker and subdomain II regions at the interface.DModel fit to map illustrated for a representative region of the 2‐domain dimeric sACE^S1211^.

Two monomer and two dimer 3D classes were selected, and each particle pool underwent a final round of 2D classification (Punjani *et al*, 2017). This revealed small regions of fuzzy density, representing glycans, on the protein surface, secondary structure in top views of the interacting dimer domains, and weak density for the rest of the molecule in frontal views with one of the domains almost completely averaged out (Fig EV3). Refinement was thus performed using the recently developed nonuniform refinement algorithm in cryoSPARC (Punjani *et al*, 2020), which is well‐suited for proteins with variations in disorder across the molecule. Global resolution values of 4.3 and 3.8 Å were obtained for the monomer and dimer refinements, respectively (Fig EV3, Appendix Fig S2A and B, Table 1, Figs 3A, and 4A).

Following 3DVA (Punjani & Fleet, 2021), local refinement with a mask around each individual domain of the consensus structure (Punjani *et al*, 2020), focused 3D classification without alignment (Scheres, 2016), and a final local refinement step of the best class (43,774 and 42,701 particles for the N‐ and C‐domains, respectively), reconstructions of the N‐ and C‐domain were generated at global resolutions of 3.7 and 4.1 Å, respectively (Figs EV3 and 3B, and Appendix Fig S2C and D). Glycan densities were observed at a low volume threshold (Fig 3A and C), and several bulky sidechains were visualized, especially in the protein core (Fig 3D and E). The sphericity (as determined by the 3DFSC server (Tan *et al*, 2017)) improved from 0.71 for the consensus structure to 0.98 for the individual domains (Table 1 and Appendix Fig S2A, C, and D).

**Table 1:** Statistics for 3D reconstruction, model refinement, and validation.

	sACE^S1211^ Monomer			sACE^S1211^ Dimer		
	Full	N‐domain	C‐domain	Full	2 N‐domains	1 N‐domain
Cryo‐EM map (EMDB)	EMD‐13797	EMD‐13799	EMD‐13801	EMD‐13802	EMD‐13803	EMD‐13804
Fitted model (PDB)	PDB: 7Q3Y	PDB: 7Q49	PDB: 7Q4C		PDB: 7Q4D	PDB: 7Q4E
Data collection						
Sample	sACE^S1211^ (apo)					
Magnification	81,000					
Voltage (kV)	300					
Total dose (e^−^/Å^2^)	43					
Electron flux (e^−^/pixel/s)	4					
Exposure time (s)	3					
Defocus range (μm)	−1.8 to −3.0					
Pixel size (Å)						
Super resolution	0.53					
Final	1.06					
Movies curated/total	7,689/11,628					
Image processing						
Initial particle images (Topaz‐picked)	1,167,502					
Final particle images	110,776	43,774	42,701	123,059	215,632 (C2 symmetry‐expanded)	
Symmetry imposed	C1					
Map resolution (Å)	4.3	3.7	4.1	3.8	3.8	3.6
FSC threshold	0.143	0.143	0.143	0.143	0.143	0.143
Local resolution range (min/max/mean, Å)	4.2/8.9/5.3	3.8/12.0/5.9	4.1/14.7/6.3	3.7/25.2/9.7	3.6/8.8/4.6	3.6/8.9/4.6
Map sharpening B‐factor (Å^2^)	−216	−150	−190	−130	−132	−148
Sphericity (out of 1)	0.714	0.980	0.977	0.870	0.903	0.920
Refinement						
Model vs data						
CC_mask_	0.78	0.70	0.75		0.80	0.76
CC_box_	0.84	0.67	0.71		0.83	0.70
CC_peaks_	0.72	0.53	0.61		0.74	0.54
CC_volume_	0.77	0.70	0.74		0.79	0.76
Composition (#)						
Nonhydrogen atoms	10,241	5,337	4,905		10,638	5,319
Residues	1,201	616	585		1,216	608
Water	1	1	0		0	0
Ligands						
Zn^2+^	1	1	0		2	1
NAG	22	14	8		34	17
MAN	5	4	1		8	4
BMA	7	6	1		12	6
FUC	1	1	0		2	1
Cl^−^	1	0	1		0	0
RMS deviations						
Bond lengths (Å)	0.006	0.008	0.007		0.007	0.008
Angles (°)	0.811	0.961	0.873		0.869	0.845
Ramachandran plot						
Favored (%)	96.83	97.07	96.74		97.52	97.52
Allowed (%)	3.17	2.93	3.26		2.48	2.48
Outliers (%)	0.00	0.00	0.00		0.00	0.00
Rama‐Z						
Whole	−1.33 (0.22)	−1.50 (0.30)	−1.44 (0.31)		−1.89 (0.21)	−1.91 (0.29)
Helix	−0.73 (0.16)	−0.60 (0.22)	−1.08 (0.23)		−1.26 (0.16)	−1.32 (−0.22)
Sheet	−0.82 (1.11)	−0.97 (1.76)	−0.64 (1.40)		0.48 (1.06)	0.65 (1.57)
Loop	−0.89 (0.29)	−1.48 (0.40)	−0.40 (0.42)		−1.09 (0.26)	−1.06 (0.37)
Cβ outliers (%)	0.00	0.00	0.00		0.00	0.00
Rotamer outliers (%)	0.19	0.19	0.00		0.00	0.00
EM Ringer	1.56	2.02	2.30		3.21	3.06
Peptide plane						
Cis proline/general	4.2/0.0	5.4/0.0	3.0/0.0		5.3/0.0	5.3/0.0
Twisted proline/general	0.0/0.0	0.0/0.0	0.0/0.0		0.0/0.0	0.0/0.0
CaBLAM outliers (%)	0.67	0.98	0.86		0.99	1.16
ADP (B‐factors) Protein (min/max/mean)	65.22/199.24/129.67	120.62/186.54/147.87	35.94/82.67/51.11		62.71/108.10/79.57	61.11/125.05/83.17
Clash score	5.30	6.06	6.44		4.58	4.73
MolProbity score	1.48	1.50	1.56		1.33	1.34

FSC, Fourier shell correlation; RMS deviations, root mean square deviations.

A similar approach was followed to reconstruct the dimer. The 4‐domain consensus structure was resolved to 3.8 Å global resolution with the two closely interacting domains well‐resolved, but the remaining two domains were only observed in the raw map and lost upon B‐factor sharpening (Fig EV3, Appendix Fig S2B, and Fig 4A). By comparing the pattern of additional glycan density around the stable dimer domains with the monomer structure and the expected pattern of glycosylation from the sequence of each domain, the former were identified as two N‐domains. The N‐domains showed only slight variations upon 3DVA (Punjani & Fleet, 2021) of the entire 4‐domain structure, while substantial C‐domain motions were observed. Particle subtraction was therefore performed with a mask encompassing the two N‐domain molecules (signal to keep) followed by focused 3D classification without alignment (Scheres, 2016), C2 symmetry expansion, and local refinement without enforcing symmetry (Punjani *et al*, 2017; Punjani *et al*, 2020; Fig EV3). This did not affect the global resolution but improved the local resolution (Table 1, Fig 4B and Appendix Fig S2E). Since 3DVA revealed flexibility within each N‐domain molecule, a single dimer N‐domain was locally refined to yield a 3.6 Å reconstruction (Fig EV3 and Appendix Fig S2F). The resolution obtained for the different forms of sACE^S1211^ and availability of crystal structures for each truncated domain allowed model building with flexible fitting in ISOLDE (Croll, 2018; Figs 3C–E, and 4C and D) and refinement in Phenix (Afonine *et al*, 2018; Appendix Fig S3A and B).

### *N*‐linked glycosylation of sACE^S1211^ is observed in the cryo‐EM maps

Although the glycosylation of human sACE expressed in CHO‐K1 cells has not yet been rigorously studied, mass spectrometry was previously performed on the truncated domains expressed in these cells. The N‐domain was glycosylated at all nine potential sites (Anthony *et al*, 2010). The testicular isoform of ACE (tACE), which is identical to the truncated C‐domain of sACE, was glycosylated at sites N648, N666, and N685 while sites N731, N913, and N1162 were detected as both glycosylated and nonglycosylated peptides (Yu *et al*, 1997). A biantennary fucosylated complex‐type glycan structure was proposed by Yu *et al* and was therefore modeled into cryo‐EM maps in the present study. Glycans were built for each dimer N‐domain at N9, N25, N45, N82, N117, N289, N416, and N480, guided by difference maps from Privateer (Agirre *et al*, 2015; Bagdonas *et al*, 2020). For the monomer, glycans were built at these N‐domain residues and the C‐domain N648, N666, N685, N731, and N913. The glycans varied in length from a single sugar to a complete pentasaccharide core (Figs 3A and C, 4A and C, and Appendix Table S1). No glycans were built at N131 or N1162, since there was insufficient density in the maps to support it, although these residues may be glycosylated.

### The sACE^S1211^ monomer domains interact and are in an open conformation

Boginskaya *et al* (2021) proposed a compact, bent sACE structure based on surface‐enhanced Raman spectroscopy (SERS) data, while Chen *et al* (2010) proposed a dumbbell‐shaped porcine sACE structure with the two domains separated by 20–25 Å based on low‐resolution negative‐stain EM data. In contrast to these studies, the overall cryo‐EM structure of monomeric human sACE^S1211^ shows an extended hourglass‐shaped conformation with minimal occlusion of surface area by the presence of a second domain (Fig 3A). The domains are distinct but closely associated and are separated by only ~5 Å (Fig 3B and C). The 29‐amino acid interdomain linker (G589‐E617) loop does not spatially separate the domains as it was expected to but is instead wrapped around the N‐domain (Fig 3C).

**Figure EV4:**
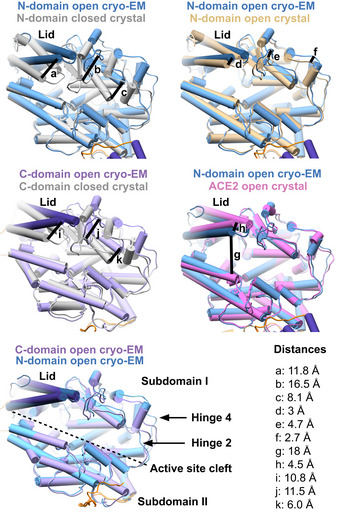
Comparison between the monomeric sACE^S1211^ structure determined here by cryo‐EM and published crystal structures Differences in active site cleft exposure are shown for the truncated single‐domain crystal structures (C‐domain PDB ID: 1O8A; N‐domain PDB ID: 4BXK for the closed conformation and PDB ID: 6ZPQ for the open conformation) and the open conformation of ACE2 (PDB ID: 1R42). Hinge regions 2 and 4, which are key for active site closure, are indicated.

Like high‐resolution crystal structures of the truncated domains, the cryo‐EM structure shows overall ellipsoid alpha‐helical domains. Each domain divides into two subdomains flanking an active site cleft that spans the entire length of the domain (~50 Å). All C‐domain structures to date showed the active site cleft occluded by a lid‐like region composed of the first three N‐terminal helices, with a small N‐terminal pore of ~9 Å in diameter. Until recently, all N‐domain structures were also in this conformation despite the ability of sACE to cleave large substrates. The latest apo N‐domain structure revealed a more open conformation (Cozier *et al*, 2020b). Interestingly, the cryo‐EM structure shows both domains in a fully open conformation for the first time (Fig EV4), reminiscent of the open conformation of ACE2 (Towler *et al*, 2004). ACE2 has 42% sequence identity and 75–76% sequence similarity to each sACE domain. The sACE^S1211^ domains are comparable to each other and to apo ACE2 (PDB ID 1R42) with overall root‐mean‐square deviation (RMSD) values of 1.4 and 1.8–1.9 Å, respectively. The N‐domain cleft is slightly more open than that of the C‐domain, and both active sites are large with respective volumes of 6,020 and 6,634 Å^3^ according to the Computed Atlas of Surface Topography of Proteins (CASTp) server (Tian *et al*, 2018). As such, the open cryo‐EM structures differ significantly from the closed crystal structures (N‐domain PDB ID 4BXK; C‐domain PDB ID 1O8A) with RMSD values of 4.9 and 3.9 Å between N‐ and C‐domain open and closed conformations, respectively. The open cryo‐EM N‐domain conformation compares well (1.6 Å RMSD) with the recently determined open N‐domain crystal structure (Cozier *et al*, 2020b). However, there are some differences: (i) the lid‐like structure comprised of the first three N‐terminal helices is shifted by ~3 Å; (ii) the antiparallel beta‐sheets of subdomain I are shifted by ~2 Å, while the loop connecting them is shifted by ~5 Å; and (iii) a loop identified previously as hinge region 2 (Lubbe *et al*, 2016) is shifted by ~3 Å (Fig EV4). These changes all contribute to a more open cryo‐EM structure. This crystal structure thus represents a partially open conformation. By comparing the monomer cryo‐EM structure to crystal structures of truncated domains complexed to ligands, intradomain hinge regions can be visualized and the full extent of ligand‐induced active site closure is appreciated (Fig EV4). C‐domain hinge points are likely to be similar to those described for the truncated N‐domain (Cozier *et al*, 2020b).

### The sACE^S1211^ monomer active site residues are primed for zinc and ligand binding

**Figure 5:**
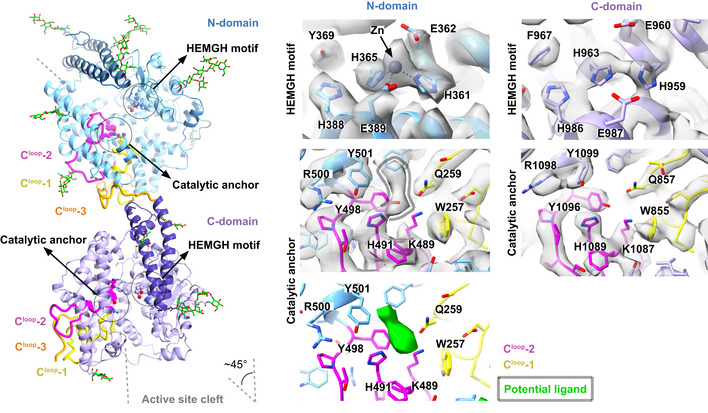
Relative domain orientation and active site architecture of monomeric sACE^S1211^ The active site clefts (gray dashed lines) of the two domains are inclined by ~45° and show a conserved zinc‐binding site (H361/959, H365/963, E362/960, and E389/987) and catalytic anchor residues (Q259/857, K489/1,087, and Y498/1,096) which are responsible for binding the substrate C‐terminus. Three C‐terminal loops (C^loop^‐1 to ‐3) in both domains are closely associated and extend from the protein surface into the active site at the protein core. The N‐domain map revealed a potential ligand in the catalytic anchor site as illustrated by the hashed region around the sharpened map (gray surface) and the fractional difference map density (green surface). Zinc was modeled for the N‐ but not the C‐domain.

Although the domains are closely associated, full‐length soluble sACE^S1211^ does not have a continuous active site channel for substrate cleavage. The N‐domain active site cleft is instead inclined by ~45° relative to the C‐domain. This incline could prevent simultaneous binding of large substrates to both domains by sterically blocking access of a long peptide to the central C‐domain cleft through the C‐domain lid region, upon exit of the peptide C‐terminus from the N‐domain prime subsite (Fig 5). Simultaneous binding of, for example, amyloid‐β to both domains is thus unlikely to explain the observed endopeptidase activity of sACE at multiple sites (Oba *et al*, 2005; Zou *et al*, 2009; Larmuth *et al*, 2016).

According to quantum mechanics/molecular mechanics (QM/MM) calculations, substrate cleavage by sACE requires zinc coordination by conserved N‐/C‐domain residues H361/959, H365/963, and E389/987, with activation of a zinc‐coordinated water molecule for nucleophilic attack by E362/960 (Wang *et al*, 2011; Zhang *et al*, 2013; Brás *et al*, 2014; Mu *et al*, 2016). In the present study, the N‐domain structure showed poor density for the E362 and E389 sidechains, but strong density for zinc coordinated by H361 and H365 in the canonical tetrahedral arrangement (Fig 5). The C‐domain H959 and H963 were in the same arrangement but zinc could not be modeled as there was no density to support it. Fractional difference map calculations (Joseph *et al*, 2020) showed extra density around N‐domain residues Q259, K489, and Y498 (Fig 5) but not their C‐domain counterparts (Q857, K1087, and Y1096). These three residues form part of the prime subsite where they are oriented towards each other and bound to either ligands or components of the crystallization condition, like acetate, in all crystal structures. Although the density is not of sufficient resolution to identify the potential ligand, the open N‐domain active site is clearly primed for substrate binding to the zinc site and these three catalytic anchor residues.

### Monomeric sACE^S1211^ undergoes bending and breathing motions

**Figure 6:**
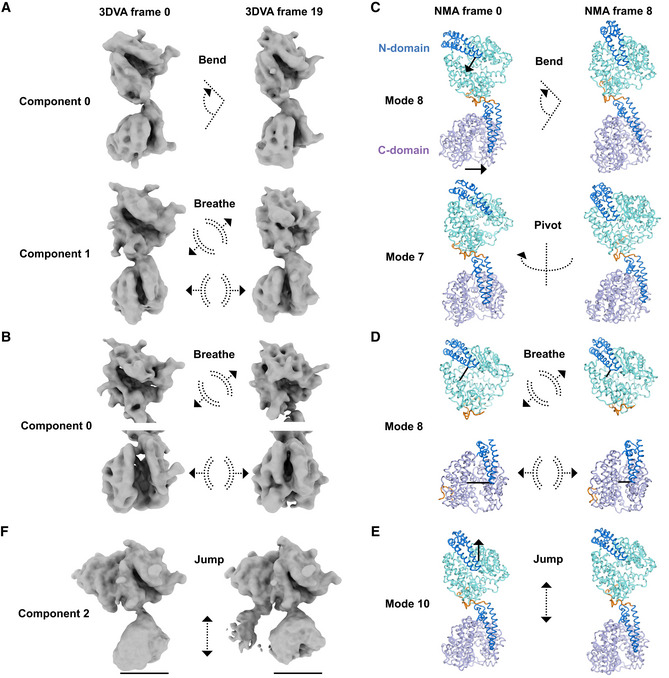
Dynamics of monomeric sACE^S1211^ A3D variability analysis (3DVA) of the monomer consensus refinement particles showing bending at the interdomain linker and breathing of each domain with opening and closing of the active site cleft. A sideways shift of the C‐domain is observed upon linker bending.B3DVA after local refinement of the N‐ (top) and C‐domain (bottom) showed breathing of the domains with opening and closing of their active site clefts.CNormal mode analysis (NMA) of the full‐length soluble monomer model. Mode 8 showed bending at the interdomain linker (orange), similar to 3DVA component 0 (in A). Mode 7 showed pivoting at the linker.DNMA of the individual N‐ and C‐domain models of monomeric sACE^S1211^ showed breathing motions like that observed by 3DVA (in B).ENMA mode 10 of the full‐length soluble monomer model showed jumping at the linker, which caused a change in the separation between the two domains.F3DVA component 2 of dimeric sACE^S1211^ showed C‐domain jumping with linker contraction, as seen for N‐domain jumping of monomeric sACE^S1211^ (NMA mode 10 in E).

**Figure EV5:**
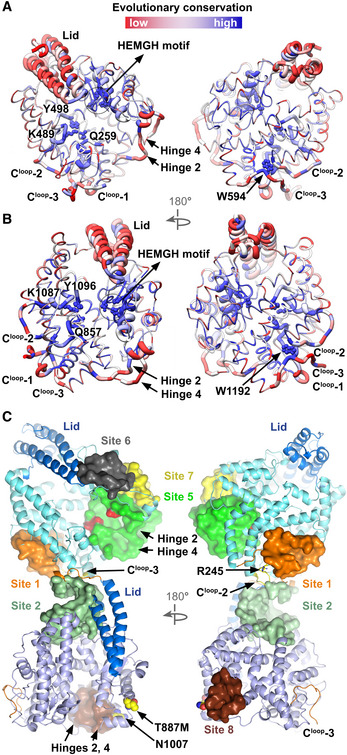
Evolutionary conservation and allosteric regulation of sACE^S1211^ Evolutionary conservation was calculated using ConSurf and mapped onto the protein structure with high and low conservation shown in blue and red, respectively. The lid region and hinges 2 and 4 were poorly conserved while the zinc‐binding motif, catalytic anchor (Q259/857, K489/1,087, and Y498/1,096), and a key tryptophan residue (W594/1,192) on C^loop^‐3 were highly conserved in both domains. The zinc‐binding motif and catalytic anchor residues are shown as spheres while C^loop^‐2, C^loop^‐3, the two hinges, and the lid region are shown as thick helices/coils. Allosteric sites were predicted for the N‐ and C‐domain orthosteric sites (zinc‐binding and catalytic anchor residues) using CorrSite2.0.
A, BN‐ and C‐domain structures, respectively, with evolutionary conservation mapped.CAllosteric sites (Sites 1–8) predicted for sACE^S1211^ shown as colored surfaces. The central active site clefts (Sites 3 and 4) allosterically regulated each other but were omitted from the figure for clarity. Sites 1, 2, and 6 were predicted to only regulate the N‐domain orthosteric site, while Sites 5 and 7 also affected the C‐domain orthosteric site. Site 8 was predicted to regulate the C‐ but not the N‐domain orthosteric site. The Alzheimer’s disease‐associated mutation T887M is shown as a yellow sphere. The unique D354 and E431 residues in the distal N‐domain prime subsite extension are shown as red spheres for orientation.

Despite the independent active site arrangement, cooperativity occurs between the sACE domains (Ehlers & Riordan, 1991; Binevski *et al*, 2003; Skirgello *et al*, 2005; Masuyer *et al*, 2015; Larmuth *et al*, 2016) and between different subsites of each domain (Lubbe *et al*, 2016; Sturrock *et al*, 2019; Cozier *et al*, 2020a). Such allosteric effects can be triggered by changes in the protein's dynamics. To gain insight into the dynamics of monomeric sACE^S1211^ and how it could regulate cooperativity, we first used 3DVA to analyze heterogeneity between experimental cryo‐EM particles from the consensus and local refinements. Bending at the interdomain linker region resulted in the swinging of the domains and variations around the N‐domain C‐terminus where the linker wraps around (Movie EV1 and Fig 6A). 3DVA of the consensus particles after local refinement of each domain (but before 3D classification without alignment) revealed breathing of each domain (Movie EV1 and Fig 6B). Active site opening/closure consisted of motion in the subdomain I lid region, subdomain II rearrangements, and bending around the previously assigned (Lubbe *et al*, 2016; Cozier *et al*, 2020b) hinge 2 (268–289/867–876) and 4 (409–417/1,006–1,015) loops located between the two subdomains (see Figs EV4 and EV5 for the location of these two hinges).

Second, we used NMA of the models to assess whether the motion observed by 3DVA represents the protein's intrinsic continuous flexibility, or if it was biased by noise/suboptimal particle alignment during reconstruction. Horizontal swinging and deep bending of the interdomain linker elicited alternative rotation of the two domains, and movement of N‐domain subdomain II towards C‐domain subdomain I or II (Movie EV2 and Fig 6C), respectively, which is in agreement with 3DVA. Active site breathing was observed by NMA of the single‐domain models (Fig 6D) and showed comparable results for each domain, with active site breathing via sliding or clamshell‐like motions of the two subdomains along the cleft (Movie EV3 and Fig 6D) in agreement with 3DVA of the N‐ and C‐domain (Movie EV1 and Fig 6A and B). NMA of the full‐length soluble monomer showed a similar sliding motion along the N‐domain cleft together with a downward shift of the full N‐domain towards the C‐domain (Movie EV2 and Fig 6E). Although this jumping motion was not observed by 3DVA of the monomer, 3DVA of the dimer showed jumping of the C‐domain (Fig 6F, see later Results section on sACE^S1211^ dimer flexibility). The interdomain linker and C‐domain lid N‐terminus thus acted like a spring. These results clearly illustrate the dynamic nature of sACE^S1211^ and emphasize that its full ensemble of states cannot be described by MD simulations/crystal structures of the truncated domains.

### A sACE^S1211^ allosteric site is formed by three C‐terminal loops

**Figure 7:**
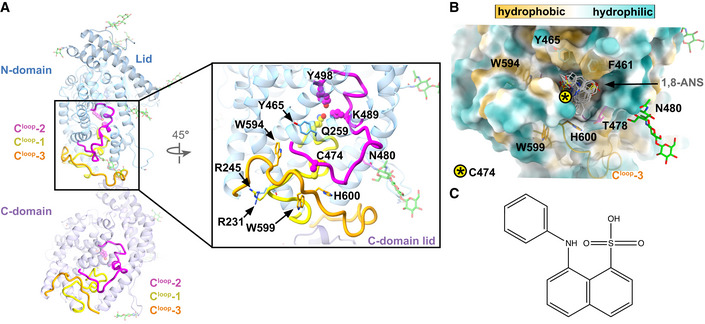
Close association between three loops at the C‐terminus of each sACE^S1211^ domain forms two hydrophobic surface pockets AThree C‐terminal loops (C^loop^‐1 to ‐3) on the protein surface are closely associated and likely stabilize the N‐domain catalytic anchor residues Q259, K489, and Y498 for ligand binding. Disruptions in these contacts could be transmitted to the protein core to allosterically alter catalytic activity since C^loop^‐1 and ‐2 extend from the surface into the active site. The C‐domain lid is next to N‐domain C^loop^‐1 and ‐3.BC^loop^‐1 to ‐3 form a hydrophobic pocket on the surface of each domain (N‐domain surface is shown). The highest‐ranking docked poses for 1,8‐ANS (1‐anilinonaphthalene‐8‐sulphonic acid) in this pocket are shown as gray lines.CChemical structure of the 1,8‐ANS molecule.

During 3DVA and NMA, variations were noted towards the N‐domain C‐terminus. Since subdomain II was similar in both open and closed N‐domain crystal structures (Cozier *et al*, 2020b), it was previously assumed to be relatively rigid. Upon closer inspection of the structures in the present study, three closely associated loops were identified in subdomain II of each domain, namely C‐terminal loop 1 (C^loop^‐1): N‐domain 238–261/C‐domain 836–859; C^loop^‐2: N‐domain 472–498/C‐domain 1,070–1,096; and C^loop^‐3: N‐domain 589–617/C‐domain 1,187–1,201 (Fig 7A). C^loop^‐1 and C^loop^‐2 extend from the surface into the active site while C^loop^‐3 wraps around the surface and passes between C^loop^‐1 and C^loop^‐2. Importantly, N‐domain C^loop^‐3 is the interdomain linker and C^loop^‐1 contains Q259/857 while C^loop^‐2 contains K489/1087 and Y498/1096. These three prime subsite residues form a critical anchor for substrate/inhibitor binding (Naqvi *et al*, 2005).

Loop regions are generally more flexible than alpha helices or beta‐sheets and often play important roles in catalysis. To investigate whether these three loops could regulate interdomain cooperativity or provide a means of allosteric regulation within each domain by passing signals from the surface to the core catalytic residues, we firstly analyzed the evolutionary conservation of sACE^S1211^ using ConSurf (Landau *et al*, 2005; Ashkenazy *et al*, 2016) because slowly evolving residues are usually more functionally important. The lid, hinge 2, and hinge 4 of both domains were poorly conserved while residues proximal to the critical zinc‐binding motif of subdomain I were highly conserved (Fig EV5A and B). Importantly, C^loop^‐1 and ‐2, which include the three catalytic anchor residues and the essential chloride‐binding R500/1098 residue of both domains (Wei, 1991), were highly conserved throughout evolution. On the protein surface, C^loop^‐2 R479/1,077 is highly conserved and likely functions to stabilize the loop, so that the catalytically essential K489/1,087 and Y498/1,096 are poised for ligand binding. While C^loop^‐3 is generally poorly conserved, W594/1,192 is highly conserved (Fig EV5A and B) and could hold this loop in place to indirectly stabilize C^loop^‐2 by slotting between two helices (207–237/805–835, and 460–471/1,058–1,069; Fig 7A).

Next, we evaluated the potential for allosteric regulation of sACE^S1211^ using the Cavity, CorrSite2.0, and CorrCys modules of CavityPlus (Xu *et al*, 2018; Xie *et al*, 2022). After protein cavity detection, CorrSite2.0 predicts allosteric sites based on the hypothesis that the motion of orthosteric and allosteric sites are highly correlated (Ma *et al*, 2016) with an accuracy of > 90% (Xie *et al*, 2022). Allosteric sites are those cavities with a *Z*‐score > 0.5 where pseudoligand binding elicits dynamic changes in the orthosteric site. For the N‐domain orthosteric site, allosteric Site 1 (*Z*‐score 1.04) was identified at the C‐terminus of the N‐domain formed by the 461–471 helix, the adjacent C^loop^‐2 residues 474–5 and 478–9, and C^loop^‐3 residues 597–600, while Site 2 (*Z*‐score 0.82) was identified between the N‐domain C^loop^‐3 and the C‐domain N‐terminus (Fig EV5C).

CorrSite2.0 showed that binding at Sites 1 or 2 allosterically affects the N‐ but not the C‐domain orthosteric site, which can be explained by the fact that C^loop^‐2 and ‐3 extend from the N‐domain surface to the key K489 and Y498 active site residues (Figs 5 and 7A). Site 1 is hydrophobic and contains the only free N‐domain cysteine residue C474 on C^loop^‐2 (Fig 7A and B) which was identified by CovCys as a covalently targetable cysteine with 73% probability. Since cysteine residues are especially prone to oxidative inactivation (Huang *et al*, 2015) and it was previously shown that binding of the hydrophobic 1‐anilinonaphthalene‐8‐sulfonic acid (1,8‐ANS) dye could protect bovine sACE from rapid inactivation by gamma radiation‐mediated water radiolysis (Voronov *et al*, 2002, 2003), we investigated whether this pocket could be the 1,8‐ANS binding site. The 1,8‐ANS molecule was blindly docked to the N‐domain using EADock at the SwissDock server (Grosdidier *et al*, 2007) revealing the binding of top‐ranking poses into allosteric Site 1 (Fig 7B and C), and substantiating our hypothesis that this surface pocket is important for catalytic activity.

CorrSite2.0 further predicted allosteric sites which can affect both orthosteric sites. The two central clefts are highly correlated: Site 3 (N‐domain cleft) affects Site 4 (C‐domain cleft) with a *Z*‐score of 3.48, and vice versa with a *Z*‐score of 3.10. Site 5 was identified in the prime subsite extension of the N‐domain cleft (Fig EV5C), distal to the orthosteric site. Site 5 is highly correlated to the N‐domain (*Z*‐score 1.69) but weakly correlated to the C‐domain (*Z*‐score 0.63) orthosteric site. Site 6, which only affected the N‐domain orthosteric site (*Z*‐score 1.15), was identified near the N‐domain antiparallel beta‐sheet loop and lid tip on subdomain I, while Site 7, which affected both domains (N‐ and C‐domain *Z*‐scores 0.93 and 1.16, respectively), was identified near N‐domain hinges 2 and 4. Site 8 is in a similar region on the C‐domain surface but does not affect the N‐domain orthosteric site. Since Sites 6–8 are regions that undergo substantial shifts upon active site closure (Fig EV4), their predicted allosteric effects are not surprising. Together, these results emphasize the importance of C^loop^‐3 and hinging for N‐domain activity and interdomain cooperativity.

### sACE^S1211^ homodimerization occurs via the N‐domain

sACE homodimerization has previously been investigated using reverse micelles, a split‐ubiquitin assay, fluorescence energy resonance transfer and small‐angle X‐ray scattering (Kost *et al*, 2000, 2003; Kohlstedt *et al*, 2006; Abrie *et al*, 2018), but these studies provided no definitive data regarding the mechanism. The native soluble sACE^S1211^ homodimer structure was determined in the present study. Although the C‐domains were unresolved due to high flexibility, the N‐domains resolved to a high resolution and allowed for the identification of the dimerization interface. To investigate how biological or “native‐like” the modeled interface is, the dimer's protein interface score (PI score; Malhotra *et al*, 2021) was determined and gave a slightly unfavorable overall PI score of −0.04 (Table EV1). However, the interface had an area of 1,054.76 Å^2^ and scored a favorable solvation free energy gain (Δ^i^G) of −7.29 kcal/mol upon formation of the complex (excluding the contribution of satisfied hydrogen bonds and salt bridges across the interface).

The *P*‐value of the observed solvation free energy gain (∆^i^G *P*‐value) of 0.27 suggests that the interface is specific. Furthermore, the TEMPy Segment‐based Manders' Overlap Coefficient (SMOC) score (Joseph *et al*, 2016), averaged over the interface residues, gave an iSMOC score of 0.842 for the 3.8 Å 2‐domain dimer map. Interfaces between chains within the asymmetric unit of available truncated N‐domain crystal structures were also analyzed using the PI‐score metric. Two crystal structures (PDB ID 2C6F and 2C6N) showed a different interface while 23 showed the same interface as the cryo‐EM structure (Table EV1). All crystal structures showed positive PI scores, but the two with a different interface scored lower overall. Although the shape complementarity of these two structures compared well to the other crystal structures, their Δ^i^G was low and their Δ^i^G *P*‐value was high, suggesting that their interface was a crystal packing artifact.

**Figure 8:**
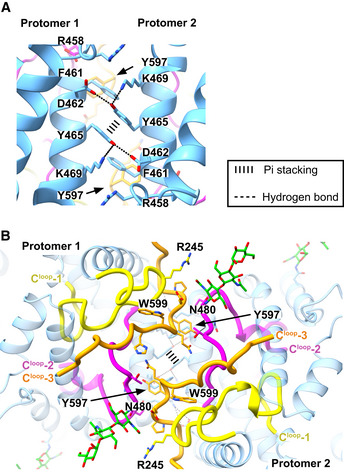
Mechanism of sACE^S1211^ dimerization between N‐domain molecules AThe C2‐symmetric dimer interface is formed by hydrogen bonds and pi‐stacking interactions. Y465 is at the centre of the interface.BThe C‐terminal loops (C^loop^‐1 to ‐3) are involved in dimerization, and the N480 glycan (green sticks) is proximal to R245 of C^loop^‐1.

The individual interface scores compared well between the cryo‐EM structure and the 23 crystal structures, except for the geometric shape complementarity score, which was lower for the cryo‐EM structure at 0.497. The dimeric sACE^S1211^ cryo‐EM structure shows a bridge formed between glycans on N82 of the two protomers (Fig 4). As these interactions likely contribute to the energy but are not considered by the current scoring algorithms, the intermediate shape complementarity could have caused the slightly negative PI score compared with the minimally glycosylated N‐domain crystal structures, although the interface is favorable. Apart from the glycan‐glycan bridge, the interface is further formed between residues of subdomain II of each N‐domain, specifically the helix of 456–471 (Fig 8A), C^loop^‐2, and C^loop^‐3 (Fig 8B). Equivalent interactions occur for both domains due to C2 symmetry. Residues from the second protomer are denoted here in italics. Residue Y465 is at the centre of the interface and forms hydrogen bonds to K469 and *D462*, and sandwich pi‐stacking with *Y465*. Y597 on C^loop^‐3 interacts with *F461* via T‐shaped pi‐stacking and hydrogen bonds with the *N460* backbone carbonyl. The interface also involves close associations between C^loop^‐2 and C^loop^‐3 of the two protomers. Here, we provide the first conclusive structural evidence that sACE^S1211^ dimerizes via this N‐domain interface in a native state.

### sACE^S1211^ dimerization increases flexibility at the allosteric site

**Figure 9:**
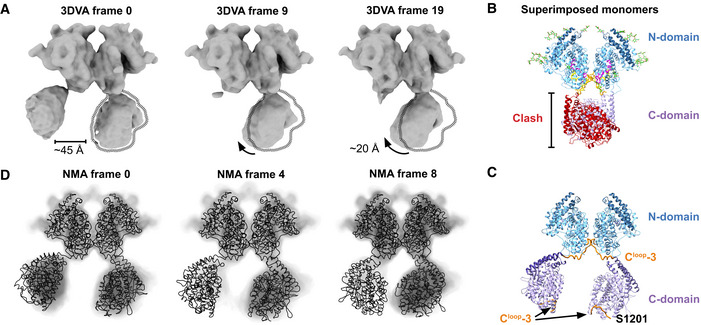
Structure and dynamics of the full‐length soluble sACE^S1211^ dimer A3DVA (component 0) of dimer particles from the consensus 4‐domain refinement showed large swinging motions of the C‐domains.BSuperposition of two full‐length soluble monomeric sACE^S1211^ chains onto the two‐domain dimeric model reveals a large steric clash between the C‐domains if the monomer linker conformation is maintained.CA hypothetical full‐length soluble dimer model built into 3DVA frame 0 of component 0 through flexible, restrained fitting of two full‐length soluble monomer copies. The linker (N‐domain C^loop^‐3) conformation differs from the monomer and the C‐domain subdomain II regions face each other, and thus, the R1203/S1204 secretase cleavage site would be shielded.DNMA performed on the full‐length soluble dimer model showed close agreement between the NMA conformations (mode 9, black tubes) and selected movie frames from experimental 3DVA conformations of the consensus 4‐domain refinement (component 0, transparent solid view).

To investigate the potential effect of dimerization on the overall conformation and dynamics of the N‐domain, the models and 3DVA/NMA results of monomeric and dimeric sACE^S1211^ were compared. Dimerization does not appear to cause large‐scale changes in the apo N‐domain conformation, since the monomer and dimer cryo‐EM N‐domain structures compare very well (0.65 Å RMSD). The active sites are in an open conformation for both states. Since the interface is essentially identical to that of ligand‐bound crystal structures, ligand‐binding and active site closure also do not alter N‐domain dimerization (Appendix Fig S4A). Like the monomer, the dimer N‐domain undergoes continuous active site breathing motions (Appendix Fig S4B). The proposed Site 1 allosteric switch of C^loop^‐1 to ‐3 is proximal to the dimerization interface (Figs 8B and EV5C). Dimerization alters the N‐domain C^loop^‐3 flexibility and drastically increases the C‐domain dynamics (Movie EV4, Figs 6F and 9A). The N‐domain contacts are likely to be very strong, since they occur and can be refined to high resolution despite this. The consensus 4‐domain dimer C‐domains showed large lateral swinging motions (Fig 9A), along with rotation, and contraction and lengthening of the interdomain linker (N‐domain C^loop^‐3; Movie EV4 and Fig 6F). The one C‐domain showed stronger density during refinement and 3DVA and is likely more stable than the second where the linker is more bent. The two C‐domains are separated by ~45 Å at their most extended conformation (component 0, frame 0, Fig 9A). Superposition of two copies of the full monomer structure onto the 2‐domain dimer structure showed that a substantial clash would occur between subdomain II of the two C‐domains in the monomer conformation (Fig 9B). The large separation observed in the most extended dimer conformation contrasts with this, suggesting that the relative domain orientation is altered upon dimerization.

Because the first frame of component 0 showed low‐resolution density for both C‐domains, the relative domain orientations were known and were used to build a hypothetical model of full‐length soluble dimeric sACE^S1211^ (Fig 9C). NMA of this model showed large ~20 Å swinging motions of the C‐domains with subdomain II of each domain facing the other (Fig 9D), and rotation at the linker region (Movie EV5). Since the extremes of 3DVA component 0 matched the extremes of NMA mode 9 very well for the more stable C‐domain (Fig 9D), this coarse dimer model seems to be reasonable. The linker thus acts as a pivot, hinge, and a spring in both states, but its dynamics are altered upon dimerization.

### Dimerization of sACE^S1211^ activates the allosteric switch

**Figure 10:**
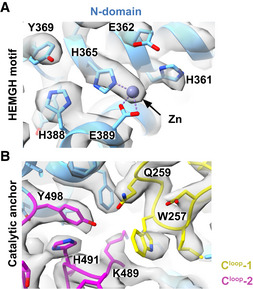
Active site architecture of dimeric sACE^S1211^ AA noncanonical H361 rotamer was seen for the HEMGH zinc‐binding motif with the sidechain pointing away from the zinc, in contrast to its orientation in the sACE^S1211^ monomer cryo‐EM structure.BA noncanonical K489 rotamer was observed with the sidechain pointing away from the catalytic anchor residues Q259 and Y498 (into the viewing plane), in contrast to its orientation in the sACE^S1211^ monomer cryo‐EM structure.

Given the altered dimer N‐domain C^loop^‐3 dynamics and proximity of the interface to Site 1, this allosteric switch could be engaged upon dimerization. To investigate this, the N‐domain active sites of monomeric and dimeric sACE^S1211^ were compared. Interestingly, the active site was altered upon dimerization. While the monomeric N‐domain showed zinc coordinated by H361 and H365, both dimer N‐domain active sites showed a rotamer flip of H361 away from the zinc, with metal coordination only via the H365 sidechain (Fig 10A). This was observed in the consensus and local refine maps, with no evidence for the canonical tetrahedral coordination. The prime subsite also differed from the monomeric N‐domain, since no evidence of ligand density was observed near the catalytic anchor residues, and the dimer K489 sidechain was flipped out from the active site. Instead of facing Q259 and Y498, as in the monomer N‐domain cryo‐EM structure (Fig 5) and all current crystal structures, the dimer K489 instead faces the backbone carbonyl of D485 and the imidazole of H164 (Fig 10B). The K489 rotamer flip in the protein core likely arose due to interactions between C^loop^‐2 (on which it resides) and C^loop^‐3 across the dimer interface, and consequent changes in their dynamics. Overall, the altered N‐domain active site suggests that dimerization induces a shift from an active to a resting or inactive state, since H361 and K489 are essential for activity (Wei *et al*, 1992; Naqvi *et al*, 2005).

## Discussion

Given the high global prevalence of cardiovascular disease and the use of ACE inhibitors for first‐line treatment (Stapff & Hilderbrand, 2019), it is crucial to develop ACE inhibitors with better safety profiles and/or novel mechanisms of action. It is therefore urgent to improve our understanding of sACE structure and function. Recent significant advancements in cryo‐EM instrumentation and image processing methods have enabled the study of sub‐100 kDa proteins to a high resolution (Herzik *et al*, 2019). However, sample heterogeneity, and especially continuous heterogeneity, can still be a limiting factor in the resolution of small proteins. Here, we have described the detailed image processing methodology used to solve the first cryo‐EM structures of human sACE. The soluble form (sACE^S1211^) of this ~170 kDa glycoprotein was studied as it occurs naturally in the blood, seminal fluid, and cerebrospinal fluid (Ehlers *et al*, 2012). The physiological relevance of sACE shedding is unknown but membrane anchoring is not expected to induce structural changes since substrate hydrolysis, inhibitor binding, and chloride activation kinetics are equivalent between soluble and membrane‐bound sACE in purified form, cell culture, or *in vivo* (Lanzillo *et al*, 1980; Wei, 1991; Jaspard & Alhenc‐Gelas, 1995; Azizi *et al*, 2000).

Unlike the crystal structures, where most glycans are removed and the protein is truncated to the N‐ or C‐domain, the cryo‐EM structures produced in the present study were of the full‐length soluble enzyme in a fully glycosylated and apo state. The protein was catalytically active, and native PAGE revealed the presence of a small fraction of dimeric sACE^S1211^. SEC yielded a single peak, and could thus not be used to separate the two discrete states *in vitro*. The sample containing this minor dimer fraction was therefore used for cryo‐EM data collection and yielded high‐resolution reconstructions of both monomeric and dimeric sACE^S1211^. Identifying particles on micrographs was challenging for several reasons: (i) the protein had a low signal‐to‐noise ratio due to its small size; (ii) the protein domains are globular and resembled noise at close‐to‐focus, even in denoised micrographs; (iii) two neighboring monomer top views could look like a monomer side view and only be picked once, thus potentially leading to a loss of particles. Once particle picking was optimized, 2D reconstruction also posed a challenge, since: (i) the size of the orthogonal monomer projections showed large disparity; (ii) the signal‐to‐noise ratio was low (especially for the monomer top view); (iii) the dimer showed high flexibility and thus poor alignment; (iv) the discrete monomer and dimer states were pooled together into the same class averages, and (v) a sizeable proportion of the mass of the particle consists of flexible glycans.

Dimer side, bottom, and top views were likely pooled together with monomer side views, as they are all essentially two neighboring globular domains and only differ in the degree of separation between them. We found that 3D classification (*ab initio* reconstruction and heterogeneous refinement) of the entire Topaz‐picked particle stack in cryoSPARC (Punjani *et al*, 2017) performed much better than 2D classification since 3D classification likely introduced less human bias than the extensive 2D classifications in RELION, and allowed separation of discrete states, although several iterations were still required. Local refinement of individual domains with the nonuniform algorithm (Punjani *et al*, 2020) allowed reconstruction to high resolution but required very soft masks (only including the protein density but with wide padding around the glycans and excluded domain) to avoid overfitting artifacts.

In the present study, we revealed the hourglass‐shaped monomeric sACE^S1211^ domain orientation and detailed dimeric sACE^S1211^ active site architecture for the first time. Both domains were in an open conformation which could support the design of allosteric inhibitors with fewer side effects than competitive inhibitors. Compounds that bind distal to the active site, and thus independent of zinc chelation, could have increased selectivity over other metalloproteases. Distal sites with less similarity could also be more suitable for domain‐selective inhibitor design than the N‐ and C‐domain active sites which are 90% identical. Clinically available ACE inhibitors are associated with the development of side effects such as persistent cough in 5–20% of patients and severe swelling below the skin surface in 0.1–0.7% of patients (Bicket, 2002; Kostis *et al*, 2018). These are caused by the accumulation of bradykinin (which is efficiently cleaved by both sACE domains) due to nonselective inhibition of both domains. Ang I is predominantly cleaved by the C‐domain, and thus its selective inhibition would lower blood pressure while allowing bradykinin cleavage through the N‐domain. The two domains' unique functions also support the development of ACE inhibitors for alternative indications. Since the antifibrotic peptide AcSDKP is selectively cleaved by the N‐domain (Rousseau *et al*, 1995), N‐domain‐selective ACE inhibitors can be used to treat fibrosis by preventing its hydrolysis without affecting blood pressure (Junot *et al*, 2001).

Although some progress has been made towards domain‐selective ACE inhibitors in the last 20 years, no compounds are clinically available at present. The mechanisms of domain selectivity are poorly understood, particularly for the N‐domain, where mutations of distal amino acids dramatically alter competitive inhibitor binding (Lubbe *et al*, 2016), yet crystal structures of the mutant and wild‐type are essentially identical (Cozier *et al*, 2020a). This highlights the existence of allostery and the limitations of these static structures. Based on the present results, the nonprime subsite below the lid region is poorly conserved between species and between the human sACE domains. Given its prominence in active site closure, and thus its likely participation in substrate hydrolysis, the inner lid region could provide the ideal site for allosteric domain‐selective ACE inhibitor design.

Crystal structures have been solved where a second ACE inhibitor molecule (Masuyer *et al*, 2014a; Cozier *et al*, 2018) or the N‐terminus of a large C‐domain‐selective peptide from snake venom bound proximal to the inner lid region (Masuyer *et al*, 2012; Sturrock *et al*, 2019). Since this pocket also contains a critical chloride ion (Yates *et al*, 2014; Masuyer *et al*, 2014b), it could be the ideal target for designing allosteric drugs that inhibit ACE by (i) blocking active site closure, and (ii) displacing the key chloride ion. This seems feasible, since a potent allosteric inhibitor bound to the equivalent inner lid region of the ACE homolog neurolysin (PDB 4FXY) locked it in an open, inactive conformation (Hines *et al*, 2014). However, the domain selectivity of such compounds should be validated on full‐length soluble sACE since the present study showed strong allosteric effects between the two active site clefts by motion correlation analysis. This analysis also identified allosteric Site 5 in the N‐domain prime subsite extension which may hold promise for N‐domain‐selective inhibitor design since it is poorly conserved between the two domains, and has a stronger effect on the N‐ than on the C‐domain. This prediction agrees with our previous findings, where S357V and E431D mutations in Site 5 altered protein hinging and decreased N‐domain‐selective inhibitor binding to the orthosteric site (Lubbe *et al*, 2016). It is interesting to note that the Alzheimer's disease‐associated T887M mutation, which drastically reduces antibody binding (Sassi *et al*, 2016; Popova *et al*, 2021), is located on C‐domain hinge 4 near Site 8 and could thus alter hinging. In agreement with the predicted allosteric effects of hinge 2 and 4 from the present study, a strong N‐domain‐selective anticatalytic effect was previously observed upon binding of monoclonal antibody i2H5 to its epitope formed by Site 7 and hinge 4 (Skirgello *et al*, 2006).

In the present study, NMA and 3DVA showed that sACE is a very dynamic molecule that undergoes continuous conformational changes both within and between the two domains through bending, pivoting, and swinging of the interdomain linker. Although intradomain hinging has been described for related peptidases like neurolysin (Teixeira *et al*, 2018), ACE2 (Towler *et al*, 2004), *Escherichia coli* dicarboxypeptidase (Comellas‐Bigler *et al*, 2005), and thimet oligopeptidase (Ray *et al*, 2004), the interdomain hinging described in the present study is unique to two‐domain sACE. Since the input particles for 3DVA were already iteratively classified in 3D to separate seemingly discrete states, the full spectrum of heterogeneity present in this dataset was likely not visualized.

We propose that three closely associated loops, C^loop^‐1 to ‐3, on subdomain II of the N‐domain allosterically regulate both domains. C^loop^‐3 (interdomain linker) wraps around the N‐domain surface between C^loop^‐1 and ‐2, which extends into the active site and harbors the critical substrate‐anchoring Q259, K489, and Y498 residues. C^loop^‐3 likely regulates interdomain cooperativity with ligand binding and active site closure of one domain affecting the other. Our study revealed that C^loop^‐3 is highly flexible and undergoes conformational changes upon active site breathing, and that the active site clefts allosterically regulate each other since their motions are highly correlated. Ligand binding to Q259 (C^loop^‐1), K489, and Y498 (C^loop^‐2) in the N‐domain active site likely alters the dynamics of the nearby C^loop^‐3, to either elicit a change in bending between the domains or in the C‐domain lid breathing motion. Likewise, ligand binding to C^loop^‐1 and C^loop^‐2 in the C‐domain active site may affect the N‐domain by causing C‐domain active site closure and lid motion adjacent to the N‐domain C^loop^‐3. Experimentally, there is a strong link between these three loops and sACE activity. Mutation of the C‐domain equivalents of K489 and Y498 (K1087A and Y1096F) or a key salt bridge between C^loop^‐1 and ‐3 (R828H mutation of the C‐domain equivalent of R230 on C^loop^‐1) was previously shown to drastically decrease sACE activity and inhibitor binding (Naqvi *et al*, 2005; Michaud *et al*, 2014). Mutation of the highly conserved C^loop^‐3 residue W594 resulted in perinatal death *in vivo*, and impaired trafficking and decreased activity for both domains *in vitro* (Michaud *et al*, 2014). In agreement with these studies, our analysis of motion correlation between protein cavities and docking of the 1,8‐ANS molecule, which is known to protect sACE against oxidative inactivation (Voronov *et al*, 2002, 2003), showed that these three loops form the allosteric Site 1 which could regulate N‐domain activity. Further research is required to understand the intra‐ and interdomain cooperativity of different ligands, potentially via all‐atom MD simulations.

It is important to fully understand the physiological effects of current clinically available compounds in the context of the present sACE structures. As expected, short‐term ACE inhibitor use leads to a decrease in Ang II levels. However, with chronic administration, Ang II levels return to baseline, while the cardioprotective effects remain (Lee *et al*, 1999; Ehlers *et al*, 2013). Under basal conditions, low levels of soluble and membrane‐bound sACE dimers were observed (Kohlstedt *et al*, 2006; Abrie *et al*, 2018). However, upon ACE inhibitor treatment, a rapid (within 2 min) and dramatic increase in dimerization were observed, which coincided with S1270 phosphorylation on the short intracellular tail (Kohlstedt *et al*, 2006). CK2‐mediated phosphorylation led to the retention of sACE on the cell membrane and decreased shedding (Kohlstedt *et al*, 2002), and initiated a signaling cascade involving mitogen‐activated protein kinase kinase 7 and c‐Jun N‐terminal kinase which altered sACE and COX‐2 expression (Kohlstedt *et al*, 2004; Reis *et al*, 2018).

Prior to the present study, there was no consensus regarding the mechanism of homodimerization. Although inhibitor binding can increase dimerization, no inhibitor was added to our sample as we aimed to solve the apo structures. Successful reconstruction of the sACE dimer to a high resolution enabled unambiguous identification of the dimerization interface between two N‐domains. The Y465 residue is at the centre of the interface. This interface was previously proposed by Danilov *et al*, based on the results of protein interfaces, surfaces and assemblies (PISA; http://www.pdbe.org/pisa) analysis, and protein–protein docking of N‐domain crystal structures (Danilov *et al*, 2011, 2021) but was inconclusive since glycosylation was limited, the protein truncated, and the crystal structure interface mediated by polyethylene glycol (PEG), which would never encounter ACE in nature (Anthony *et al*, 2010). Close glycan‐glycan interactions between the N82 sites of interacting N‐domains contribute to sACE^S1211^ dimerization, in agreement with the observation by Kost *et al* that dimerization in reverse micelles is inhibited by galactose (Kost *et al*, 2000, 2003). However, Kohlstedt *et al* did not observe significant changes in the presence of sugars. The glycan‐glycan interactions shown in the present study likely contribute to, but are not essential for, dimerization.

The interdomain linker (N‐domain C^loop^‐3) flexibility was significantly increased upon dimerization and resulted in large C‐domain swinging motions. This increased flexibility could explain why basal dimerization in cell culture was less efficient for sACE than for truncated N‐ or C‐domain (Abrie *et al*, 2018). The superposition of two monomer chains to the dimer's interacting domains showed overlapping C‐domains; thus, dimerization requires linker rearrangements to avoid this clash. We built a hypothetical full‐length soluble dimer model based on a 3DVA frame of the most extended dimer. The C‐domains' subdomain II regions face each other, thereby occluding the R1203‐S1204 sheddase recognition site on C^loop^‐3 of the C‐domain, and the entire dimer was surrounded by a shield of glycans. This suggests that dimerization decreases shedding from the cell membrane, in agreement with previous reports where inhibitor‐induced dimerization of membrane‐bound sACE increased S1270 phosphorylation (Kohlstedt *et al*, 2006) and phosphorylation decreased sACE shedding (Kohlstedt *et al*, 2002). This also agrees with the findings of Danilov *et al*, where a Y465D mutation dramatically increased shedding (Danilov *et al*, 2011), since in our structure, this mutation would probably cause charge repulsion and decreased dimerization. Inhibitor binding likely stabilizes the dimer and reduces C‐domain steric hindrance by triggering active site closure and decreasing the domain volumes. An inhibitor‐bound dimer reconstruction would be required to test this hypothesis.

We observed close associations between C^loop^‐1 to ‐3 of the interacting N‐domains at the dimerization interface, and propose that these interactions and increased linker flexibility led to the observed noncanonical H361 and K489 sidechain orientations of the dimer. The C‐domain swinging motion likely destabilized the N480 glycans, or interactions between the loops, to disrupt the stacking interaction of W257 (C^loop^‐2) with K489 (C^loop^‐3), and ultimately cause the K489 rotamer flip. Dimerization appears to activate the proximal allosteric switch (Site 1) and shift sACE^S1211^ from an active to an inactive or resting state, although this should be investigated further. This could be the ideal pocket to target for stabilization or induction of dimerization. The single free N‐domain cysteine C474 is located in this pocket and is separated by ~17 Å between interacting N‐domains. The design of a molecular tether based on 1,8‐ANS to covalently link these pockets could allow studies into the effect of dimerization on sACE activity and intracellular signaling. If dimerization indeed decreases sACE activity and elicits beneficial signaling, a molecule that shifts the equilibrium from monomer to dimer could be a viable novel therapeutic agent.

The results from this study also have diagnostic applications. Although sACE glycosylation occurs to varying degrees in different tissues (Kryukova *et al*, 2015) the structural and functional relevance of this tissue specificity is unknown, but is important since it affects antibody binding and thus the diagnosis of diseases where sACE is upregulated (Danilov *et al*, 2010, 2018, 2021; Danilov, 2017). The present study paves the way for investigations into the detailed glycan structures of different sACE glycoforms using MD simulations along with comprehensive mass spectrometry analysis and will aid the development of monoclonal antibodies by allowing for enhanced epitope mapping (Popova *et al*, 2021).

In conclusion, the monomeric and dimeric cryo‐EM structures determined here provide novel insights into the structure of sACE, its complex mechanisms of action, and have important implications for future research into its inhibition and the development of novel therapeutic interventions.

## Materials and Methods

### Constructs and protein expression

The sACE^S1211^ construct used for protein expression encodes a form of sACE which has been engineered to enhance the secretion of soluble sACE into the culture medium. The pcDNA^3.1+^‐sACE^S1211^ construct, which was previously stably transfected into Chinese hamster ovary (CHO‐K1) cells (Redelinghuys, 2006), was expressed in the current work using the CelCradle™ Benchtop Bioreactor (Esco VacciXcell) according to the manufacturer's protocol.

### Protein purification

Protein purification from the culture medium was performed using lisinopril–Sepharose affinity chromatography, as previously described (Bull *et al*, 1985). Briefly, Sepharose beads which have been chemically coupled to the potent ACE inhibitor lisinopril were packed and the column equilibrated with 20 mM HEPES (pH 7.5) containing 500 mM NaCl at a flow rate of 1.5 ml/min. All buffers used for protein purification and dialysis were prepared using deionized Milli‐Q^®^ water with a resistivity of 18.2 MΩ.cm (Millipore). Harvested culture medium was loaded onto the column at a flow rate of 3 ml/min after which the column was washed using equilibration buffer at a flow rate of 1.5 ml/min. The protein was subsequently eluted using 50 mM Boric acid (pH 9.5) at a flow rate of 3 ml/min. Elution was monitored by a Bio‐Rad Econo UV Monitor (0.05 AUFS, Bio‐Rad Laboratories, Inc.) and Ross Chart Recorder. Fractions were collected and assayed for activity (see Materials and Methods section on ACE activity assay) after which peak fractions with activity were pooled into SnakeSkin^®^ dialysis tubing (Pierce Biotechnology Ltd.). The protein was dialyzed for 6 h at 4°C in 5 mM HEPES (pH 7.5) followed by second dialysis in 50 mM HEPES (pH 7.5) for 24 h at 4°C. The dialyzed protein was concentrated to 3 mg/ml using an Amicon^®^ Ultra 30 kDa molecular weight cut‐off centrifugal filter (Millipore), filtered using a 0.45 μm Nanosep MF GHP centrifugal filter (Pall LifeSciences), and stored as single‐use aliquots in liquid nitrogen.

### Characterization of protein size and purity

Protein purity was assessed via SDS–PAGE and Coomassie staining. An 8% acrylamide resolving gel was used. The concentration of pure sACE^S1211^ was determined using the Bio‐Rad Bradford Protein Assay according to the manufacturer's protocol with calibration against IgG (Bio‐Rad Laboratories Inc.). The native state of purified sACE^S1211^ was analyzed using native PAGE. Stacking and resolving gels were prepared to 4 and 6% acrylamide, respectively, and the running buffer consisted of 25 mM Tris–HCl (pH 8.3) with 125 mM NaCl. The sACE^S1211^ sample, bovine serum albumin (BSA) standard, and gel filtration standards (Bio‐Rad Laboratories Inc.) were prepared in sample buffer containing 125 mM NaCl and, for sACE^S1211^, 10 μM ZnCl_2_. Gels were run on ice at 25 and 100 mA for the stacking and resolving stages, respectively, and stained with AcquaStain (Bulldog Bio Inc). Native sACE^S1211^ was further analyzed by size‐exclusion chromatography on an Agilent 1260 Infinity HPLC system with a BioSEP™ SEC S3000 column (Phenomenex). The column was equilibrated using 5 mM Hepes (pH 7.5) with 125 mM NaCl and operated at a flow rate of 0.5 ml/min. Absorbance was monitored at 280 nm and compared between sACE^S1211^, gel filtration standards (Bio‐Rad Laboratories Inc.), and truncated glycosylated N‐ or C‐domain. The truncated proteins were expressed and purified separately as detailed in (Kroger *et al*, 2009). Loading volume for each sample was equal to 5 μg of truncated or full‐length soluble sACE.

### ACE activity assay

The catalytic activity of purified sACE^S1211^ was assessed according to the protocol by Schwager *et al* (2006) using the fluorogenic substrate *Z*‐phenylalanyl‐histidyl‐leucine (*Z*‐FHL; Bachem Ltd.) and a fluorescence spectrophotometer (Cary Eclipse Varian Inc.). Briefly, protein samples were diluted in 100 mM K_2_HPO_4_/KH_2_PO_4_ (pH 8.3), 300 mM NaCl, 10 μM ZnSO_4_, and incubated with 1 mM *Z*‐FHL for 15 min at 37°C. The reaction was stopped by the addition of 0.28 M NaOH, followed by derivatization of the HL product with *o*‐phthaldialdehyde (Sigma‐Aldrich^®^ Co.), incubation for 10 min at room temperature, the addition of 3 M HCl, and reading of fluorescence intensity at excitation and emission wavelengths of 360 and 385 nm, respectively. One milliunit (mU) of sACE^S1211^ activity equals 1nmol of HL product per min.

### Cryo‐EM grid preparation and data collection

Purified sACE^S1211^ at a final concentration of 1.5 mg/ml in 50 mM Hepes (pH 7.5), 125 mM NaCl and 10 μM ZnCl_2_ was incubated for 30 min on ice. These essential cofactors were added to ensure that the protein is imaged in an active state. Quantifoil^®^ 200‐mesh R2/2 copper grids were glow discharged for 30 s after which 2.5 μl of sACE^S1211^ was applied and incubated on the grid for 30 s in a Vitrobot Mark IV (Thermo Fisher Scientific) chamber at 100% humidity and 4°C. The grid was blotted for 3.5 s and vitrified by plunging into slushy ethane. Grids were stored under liquid nitrogen. Electron micrograph movies were recorded using a Titan Krios (Thermo Fisher Scientific) equipped with a K3 direct electron detector (Gatan) and high‐brightness field emission gun (X‐FEG) operated at 300 kV in super‐resolution mode at a magnification of 81,000× corresponding to a calibrated pixel size of 0.53 Å per pixel. A total of 11,628 micrograph movies consisting of 40 frames each were automatically acquired from 35 grid squares of a single grid using EPU (Thermo Fisher Scientific) with aberration‐free image shift (AFIS), defocus range of −1.8 to −3.0 μm, dose per frame of 1.075 e^−^/Å^2^, and exposure time of 3 s for a total dose of 43 e^−^/Å^2^.

### Initial image processing

The first round of image processing was performed in RELION version 3.1 (Scheres, 2012; Zivanov *et al*, 2018). Dose‐fractionated movies were corrected for beam‐induced motion using MotionCor2 (Zheng *et al*, 2017; 5 × 5 patches, dose‐weighting, binning by a factor of 2 to a pixel size of 1.06 Å, and gain correction by flipping the gain reference upside down). Contrast Transfer Function (CTF) estimation was performed using CTFFIND‐4.1 (Rohou & Grigorieff, 2015). Micrographs were manually curated using sorting by rln_CtfFigureOfMerit with a cut‐off of 0.05 and exclusion of micrographs with prominent ice rings. The resulting dataset of 7,689 micrographs was used for downstream image processing. Initial particle picking was done using six 2D class averages from 1,382 particles obtained during screening on a Tecnai F20 microscope with US4000 CCD detector (Gatan). These included what appeared to be monomer and dimer top and side/frontal views (Fig EV1). Picking from a subset of 1,000 micrographs yielded 112,751 particles which were extracted at 4.028 Å/pixel in a 60‐pixel box and subjected to five rounds of 2D classification. As most class averages appeared to represent monomeric sACE, dimer class averages were discarded and 35,408 particles were used to create an initial 3D reconstruction of monomeric sACE. It was used as a reference for 3D refinement and yielded a similar low‐resolution map for these 35,408 particles. This map was used as a reference for autopicking from all 7,689 micrographs (lowpass‐filter 20 Å, 3D angular sampling 30°, in‐plane angular sampling 5°, picking threshold 0.5, minimum inter‐particle distance 100 Å). The 1,855,423 coordinates obtained were manually curated to exclude picks on ethane contamination, micrograph edges, or carbon edges. A final set of 1,480,114 coordinates were extracted at 3.18 Å/pixel with a box size of 104 pixels. This was predicted to be large enough to accommodate monomeric and dimeric sACE particles. Two rounds of 2D classification (*T* = 2, *K* = 200, 25 iterations) were performed to remove junk particles. Again, since most class averages appeared monomeric, dimeric class averages were discarded. A coarse search (24‐/4‐pixel range/step, 5°) was required as the initial coordinates were not centred. Additionally, the expectation/alignment step (*E*‐step) was limited to 10 Å resolution. A stack of 1,254,521 monomer‐like particles were selected and underwent a third round of 2D classification (*T* = 2, *K* = 200, 25 iterations, *E*‐step limit 6 Å, 5‐/1‐pixel range/step, 5°). The resulting 2D class averages still showed evidence of junk (e.g., micrograph edges) and dimer‐like particles (three or four globular domains) in addition to the monomer top (single globular domain) and side (two connected globular domains) view particles. Thus, dimer and monomer particles were pooled into the same 2D class averages and not adequately separated. The elongated nature of monomeric sACE and difference in top and side view diameters (~60 vs. ~180 Å, respectively) likely also resulted in suboptimal alignment of the particles when using a uniform box size, as previously seen for alcohol dehydrogenase (Herzik *et al*, 2019). Class average of the monomeric top (355,239 particles) and side (678,179 particles) views were therefore selected and underwent separate 2D classifications (25 iterations, *T* = 2, *K* = 200, *E*‐step limit 6 Å, 5‐/1‐pixel range/step, 5°) with mask diameters of 120 and 200 Å for the top and side views, respectively. Top views underwent one round of 2D classification, while side views underwent four rounds of 2D classification during which dimer classes still separated out. The resulting stacks of 275,142 top view particles and 397,765 side view particles were combined. An initial 3D reconstruction was generated, centred, and 3D classification performed with coarse sampling (*T* = 4, *K* = 6, 60 Å reference lowpass, *E*‐step limit 12 Å, 30‐/5‐pixel range/step, 7.5°) for 11 iterations and finer sampling (5‐/1‐pixel range/step, 7.5°) for 13 iterations. A wide circular mask diameter of 311 pixels was set to allow detection of any remaining dimer classes. Two 3D classes (37,868 particles at ~16 Å resolution, and 604,420 particles at ~14 Å resolution) were selected, pooled, and the particles re‐extracted with recentering at 1.06 Å/pixel in a 360‐pixel box. The particles underwent two further rounds of 2D classification (*T* = 2, *K* = 200, *E*‐step limit 7 Å, 5‐/1‐pixel range/step, 5°) to yield 536,833 monomer‐like particles. A 240 Å mask diameter was used for all monomer views, i.e., top and side views were not separated. One round of 3D classification was performed (*T* = 4, *K* = 6, *E*‐step limit 7 Å, 5‐/1‐pixel range/step, 3.7° with local searches in a 5° range) for 22 iterations. One class (142,185 particles at ~14 Å resolution) was selected and refined (initial lowpass of 60 Å, initial translational search range/step of 10/2 pixels, respectively, and angular sampling of 7.5°) to yield a 10 Å resolution reconstruction. A final round of 3D classification was performed as before, except with *K* = 3. One 3D class (31,875 particles at ~12 Å resolution) was selected and refined as before but with local angular searches from 1.8°, a soft mask around the protein, and solvent‐flattened FSCs. This resulted in a ~7 Å resolution reconstruction. To test whether the resolution was limited by the box size, these refined particles were re‐extracted in a 560‐pixel box and refined as before. No improvement was observed and the resolution was thus not limited by box size.

### Final image processing

The first round of image processing confirmed the presence of dimer particles, but dimeric sACE could not be reconstructed as most 2D class averages appeared monomeric. Monomeric sACE, on the other hand, was reconstructed but its resolution was likely limited by the low particle number and/or heterogeneity. To obtain a better initial particle stack, potentially containing sufficient monomeric and dimeric sACE particles to yield a high‐resolution 3D reconstruction, Topaz version 0.2.5 (Bepler *et al*, 2019) was employed. Scripts for Topaz integration in RELION version 3.1 (Scheres, 2012; Zivanov *et al*, 2018) were obtained from Github (https://github.com/tbepler/topaz/). The set of 7,689 curated micrographs was denoised using the pretrained Topaz‐denoise (Bepler *et al*, 2020) denoising model (unet model, patch size 1,280, patch padding 384) to improve particle visualization (Fig EV2). The 31,875 particles of the ~7 Å monomer reconstruction were used as positive labels to train a picking model for sACE. These particles were distributed across all micrographs and thus not manually curated. The picking model was trained on all denoised micrographs using the resnet8 model (10 epochs, number of particles per micrograph 100, scale factor 4, radius 3) which has a receptive field of 70 pixels and would accommodate monomeric or dimeric sACE after 4× downsampling (diameters for the longest dimension of monomeric and dimeric sACE were expected to be ~33 and ~42 pixels, respectively). Particles were picked from a subset of 115 denoised micrographs (representing a range of defocus values) using the trained model with a scale factor of 4, radius of 10, pick threshold of −6, and select threshold of 0. As the positive labels used to train this model were not manually curated, some false positives and negatives were obtained. Particle coordinates were therefore manually curated by visually inspecting the 115 denoised micrographs. Picks on aggregation were excluded with the monomeric top (single globular domain), side (two neighboring globular domains), and dimeric (3 or 4 globular domains) views kept and manually picked/recentered where applicable. The selected 3,581 coordinates were extracted from the original noisy micrographs, and these particles were used as manually curated positive labels. Since the low signal‐to‐noise ratio of sACE on the original noisy micrographs precluded optimization of training and picking parameters, two Topaz models were trained in parallel on a subset of 450 denoised micrographs and their corresponding original noisy micrographs. The same settings were used as with the first Topaz training, except for the positive labels and that the number of particles per micrograph was set to 50. Particles were picked from the subset of 450 denoised micrographs using the model trained on denoised data, and the optimal parameters were identified as a scale factor of 4, radius of 13, pick threshold of −6, and select threshold of −3. These parameters were subsequently used with the model trained on noisy data to pick from 450 noisy micrographs. The resulting coordinates roughly matched those obtained and visually confirmed as true positives on denoised data, thus the model trained on noisy data was reliable. Picking from all 7,689 noisy micrographs yielded 1,727,045 particles which were extracted at 3.18 Å/pixel in a 112‐pixel box and subjected to one round of 2D classification (25 iterations, *T* = 2, *K* = 200, *E*‐step limit 7 Å, 10‐/2‐pixel range/step, 5°) to remove junk particles. A total of 1,167,502 particles were selected for further processing. Reconstruction was first attempted using iterative 2D and 3D classification in RELION (Scheres, 2012; Zivanov *et al*, 2018). At each stage, monomeric and dimeric class averages were selected, and particles of each species were joined from the various stages. The globular nature and small size of the sACE domains, and the similarity between dimer and monomer views, made it difficult to separate the two species and human bias likely contributed to the low resolution and, in the case of the dimer, strongly preferred orientation of the resulting reconstructions.

The 1,167,502 Topaz‐picked particles were therefore imported to cryoSPARC version 3.2 (Punjani *et al*, 2017) and split into three 3D classes using *ab initio* reconstruction (class similarity of 0) and heterogeneous refinement. This was followed by duplicate removal from each class and re‐extraction at 2.12 Å/pixel in a 256‐pixel box. This box size allowed for the removal of particles with nearby neighbors or micrograph edges/contaminants. Class 0 (306,470 particles) and class 2 (280,468 particles) seemed to only consist of a single globular domain each. *Ab initio* reconstruction (two classes and class similarity of 0) and heterogeneous refinement, however, separated each of these seemingly single‐domain classes into a 2‐domain class (*a*: 178,996 particles, ~9.5 Å resolution; *c*: 166,853 particles, ~10.9 Å resolution; Fig EV3) and a single‐domain class where a short stump of density was observed at the interdomain linker region (*b*: 127,474 particles, ~9.8 Å resolution; *d*: 113,615 particles, ~11.4 Å resolution; Fig EV3). Particles from *b* and *d* were discarded. Class 1 (501,893 particles) showed two linked globular domains and was separated into 4 classes by a second round of *ab initio* reconstruction (class similarity of 0) and heterogeneous refinement. Class 1 subclasses 0 (155,175 particles, ~7 Å resolution) and 2 (117,579 particles, ~8.4 Å resolution) showed two linked globular domains while subclasses 1 (123,131 particles, ~6.5 Å resolution) and 3 (42,568 particles, ~9 Å resolution) showed three and one globular domains, respectively. Class 1 subclass 1 was subjected to a third round of *ab initio* reconstruction (class similarity of 0) and heterogeneous refinement, which allowed for the separation of 28,895 junk particles (particles with close neighbors or micrograph edges; class *e* on Fig EV3) from the 3‐domain class *f* (94,236 particles, ~6.5 Å resolution). A third round was also performed for Class 1 subclasses 0, 2, and 3. For the latter, classes *g* (21,705 particles) and *h* (20,859 particles) were obtained and thus discarded. Subclasses 0 and 2 yielded two‐domain classes *i*‐*l* (Fig EV3). Classes *a*, *c*, *i*, and *j* were pooled (501,024 particles) and subjected to *ab initio* reconstruction (class similarity of 0.1) and heterogeneous refinement into four classes. Classes *n* and *o* were discarded as *n* (89,257 particles) contained junk while *o* (125,149 particles, ~9.6 Å resolution) showed two globular domains with a slightly longer interdomain linker, which could not be subclassified or further refined likely due to remaining heterogeneity (Fig EV3). Class *p* (155,066 particles, ~7 Å resolution) showed two linked globular domains and clear glycan stumps on the surface. Class *m* (131,552 particles, ~8.3 Å resolution) also showed two linked globular domains. The latter underwent another round of *ab initio* reconstruction (class similarity 0.1) and heterogeneous refinement into two classes. Surprisingly, this yielded a two‐domain class *r* (77,169 particles, ~8.8 Å resolution) and a four‐domain class *q* (54,383 particles, ~8.6 Å resolution), each with clear glycan stumps on the surface (Fig EV3). The 77,169 and 155,066 monomer particles from the clear two‐domain classes *r* and *p* were pooled while the 94,236 and 54,383 dimer particles from the clear four‐domain classes *f* and *q* were pooled. Subsequently, the two‐domain monomer and four‐domain dimer particles were processed separately.

The monomer was reconstructed as follows. The stack of 232,235 particles was subjected to *ab initio* reconstruction (class similarity of 0.5) and heterogeneous refinement to generate four classes of which two were discarded. Particles from the two selected classes (each ~7–8 Å resolution) were pooled and re‐extracted at 1.06 Å/pixel in a 256‐pixel box, and underwent one round of 2D classification (Punjani *et al*, 2017; 20 online‐EM iterations, maximum resolution 6 Å, 100 classes) to remove any remaining junk. A total of 81 class averages (121,372 particles) were selected, and duplicates were removed within 20 Å for a final stack of 110,776 particles (Fig EV3). At this point, patch CTF estimation was performed on the micrographs and applied to the particles. Nonuniform refinement (Punjani *et al*, 2020; initial lowpass 20 Å, dynamic mask threshold 0.15, dilation 6 pixels, padding 34 pixels) was performed on these particles for a final two‐domain monomer reconstruction at 4.3 Å resolution. 3D variability analysis (Punjani & Fleet, 2021; 3DVA) revealed flexibility at the interdomain linker. A mask (dilation 2 pixels, padding 28 pixels) was created around each individual domain using the Volume Eraser tool in Chimera version 1.15 (Pettersen *et al*, 2004) and Volume Tools in cryoSPARC (Punjani *et al*, 2017), and each domain locally refined without particle subtraction to yield 3.9 and 4.2 Å resolution reconstructions for the N‐ and C‐domain, respectively. These particles were exported to RELION (Scheres, 2012; Zivanov *et al*, 2018) using pyem (Asarnow *et al*, 2019) and subjected to focused 3D classification without alignment (*K* = 6, *T* = 40, 30 iterations) with a mask (dilation 2 pixels, padding 5 pixels) around each respective domain. One class was selected for each domain and imported to cryoSPARC (Punjani *et al*, 2017). Local refinement with a soft static mask (dilation 3 pixels, padding 35 pixels) yielded final reconstructions for the N‐domain (43,774 particles) and C‐domain (42,701 particles).

The dimer was reconstructed as follows. The stack of 148,619 dimer particles was re‐extracted at 1.06 Å/pixel in a 360‐pixel box and subjected to one round of 2D classification (Punjani *et al*, 2017; 20 online‐EM iterations, maximum resolution 6 Å, 100 classes). A total of 70 class averages (130,675 particles) were selected, and duplicates were removed within 20 Å for a final stack of 123,059 particles (Fig EV3). At this point, patch CTF estimation was performed on the micrographs and applied to the particles. Nonuniform refinement (Punjani *et al*, 2020; initial lowpass 20 Å, dynamic mask threshold 0.2, dilation 14 pixels, padding 20 pixels) was performed for a final four‐domain dimer reconstruction at 3.79 Å resolution. 3DVA showed pronounced flexibility of two domains. The particles were therefore exported to RELION (Scheres, 2012; Zivanov *et al*, 2018) for particle subtraction using pyem (Asarnow *et al*, 2019). A mask (dilation 6 pixels, padding 12 pixels) encompassing the two stable domains was created from the four‐domain dimer reconstruction using the Volume Eraser Tool in Chimera (Pettersen *et al*, 2004) and Volume Tools in cryoSPARC (Punjani *et al*, 2017). Particle subtraction was performed in RELION (Scheres, 2012; Zivanov *et al*, 2018) and followed by focused 3D classification without alignment (*K* = 3, *T* = 40, 25 iterations) of the two stable domains. A single class of 107,816 particles was imported to cryoSPARC (Punjani *et al*, 2017). Nonuniform refinement (initial lowpass 20 Å, static mask with 6 pixels dilation and 30 pixels padding) gave a 2‐domain dimer reconstruction at 3.87 Å resolution with clear C2 symmetry. The particles underwent C2 symmetry expansion. Local nonuniform refinement of the 215,632 particles (with C1 symmetry enforced) yielded a 3.78 Å resolution two‐domain reconstruction. Following 3DVA, local nonuniform refinement was performed without particle subtraction with a mask (dilation 5 pixels, padding 35 pixels) around a single dimer domain. Resolution values quoted for nonuniform and local, but not heterogeneous, refinements were according to the gold‐standard FSC at a threshold of 0.143.

### 3D variability analysis (3DVA)

3DVA (Punjani & Fleet, 2021) was performed in cryoSPARC (Punjani *et al*, 2017) using an 8 Å filter for the monomer consensus (six components) and local refinement particles (three components each), a 10 Å filter for the dimer consensus particles (five components), and a 6 Å filter for the dimer local refinement particles (three components). The latter was performed on the particle stack following C2 symmetry expansion and local refinement in C1 symmetry. In all cases, 3DVA was performed using C1 symmetry. The results were filtered to the gold‐standard FSC of the respective input refinements and visualized as 20‐frame movies with a min/max range percentile of 3%.

### Map postprocessing

Global B‐factor sharpening was performed in cryoSPARC (Punjani *et al*, 2017) using the B‐factor determined during local or consensus nonuniform refinement. To aid model building, the final half‐maps were also subjected to model‐free density modification by phenix.resolve_cryo_em in Phenix version 1.19.2 (Echols *et al*, 2012; Afonine *et al*, 2018; Terwilliger *et al*, 2020) using the auto‐tightened FSC mask from cryoSPARC refinement. Local resolution values of the final reconstructions were estimated in RELION (Scheres, 2012; Zivanov *et al*, 2018). Orientational distributions were calculated using the 3DFSC server (Tan *et al*, 2017; https://3dfsc.salk.edu) with the auto‐tightened FSC mask from the final cryoSPARC nonuniform/local refinements applied with a default cone angle of 20.0, FSC cut‐off of 0.143, sphericity threshold of 0.5, and high‐pass filter of 150.0 Å.

### Model building

As the two domains of sACE are very homologous, their identities were not immediately apparent from the protein density maps. However, visual inspection of the glycan stump positions on the final maps with the truncated crystal structures of the two domains as a guide for the potential locations of *N*‐glycans, allowed for the identification of the domains. Additionally, the N‐terminal lid region formed by the first three helices of each domain also served as a marker. The stable dimer domains were identified as two molecules of the N‐domain of sACE. Chain B from PDB ID 6ZPQ (truncated N‐domain crystal structure; https://doi.org/10.2210/pdb6ZPQ/pdb) was therefore used as the initial model for the dimer. All glycans and ligands (except the active site zinc) were removed, residues mutated in the crystal structure restored to the wild‐type sequence of sACE (Uniprot P12821, except for Pro576Leu, which is present in the construct used here), and the protein rigidly docked into the 3.63 Å single‐domain dimer map using Chimera (Pettersen *et al*, 2004). Flexible fitting of all models was performed using ISOLDE version 1.2.0 (Croll, 2018) in ChimeraX version 1.2.5 (Pettersen *et al*, 2021). Fitting was first performed for the single‐domain dimer map as it had the highest resolution. Adaptive distance and torsion restraints (Croll & Read, 2021) were applied to the protein with additional distance restraints (500 kJ/mol/Å^2^) applied at the zinc coordination site, followed by bulk flexible fitting of the entire chain. After release of the adaptive restraints, localized flexible fitting was performed with inspection of each residue at least once. The density‐modified, globally sharpened, and unsharpened maps were used. Adjustments were made where necessary, and missing residues and glycans were built using ISOLDE (Croll, 2018). A copy of this model was rigidly docked into the second dimer domain using the 3.78 Å two‐domain dimer map and relaxed into the density with a short, restrained simulation. Each residue was visually inspected as before to confirm optimal fit. As the zinc site geometry differed from that observed in the crystal structures, the distance restraint between H361 and zinc was released, and the residue was adjusted to fit the density. C‐domain models were not built for the dimer as the density was disordered. The monomer N‐domain was built by rigidly docking one chain of the dimer N‐domain model into the 3.72 Å monomer N‐domain map. Each residue was again inspected and adjusted where necessary, as before. In this case, zinc coordination was in line with the crystal structure and was thus restrained as such. The monomer C‐domain was built using PDB ID 6ZPU (https://doi.org/10.2210/pdb6ZPU/pdb) as an initial model. Residues were renumbered to sACE numbering and bulk/localized fitting into the 4.08 Å C‐domain map performed as for the N‐domain. A chloride ion was modeled at the Chloride (II) site in the C‐domain, while zinc was removed from the final C‐domain structure as there was no density to support it. The interdomain linker was built using the AlphaFold structure prediction for sACE (https://alphafold.ebi.ac.uk/entry/P12821; Jumper *et al*, 2021) as an additional guide. Although the protein active sites are not in the same conformation as observed here and the relative domain orientation prediction of low confidence, the interdomain linker prediction proved useful for building into the disordered density in this region after alignment of the C‐domain N‐terminus. To obtain a full‐length soluble sACE^S1211^ monomer model, the N‐ and C‐domain models built into local refinement maps were merged and fitted into the consensus 4.34 Å monomer map from nonuniform refinement using a short bulk flexible fitting simulation with adaptive distance and torsion restraints, and zinc distance restraints enabled.

### Model refinement and validation

Refinement was performed using real‐space refinement in Phenix version 1.19.2 (Afonine *et al*, 2018). Global minimization was done using the globally sharpened map without Ramachandran restraints but with harmonic restraints to the input reference model from ISOLDE (Croll, 2018). Comprehensive validation and EM Ringer assessment (Barad *et al*, 2015) were performed in Phenix (Afonine *et al*, 2018) against the globally sharpened map. Glycan validation was performed using Privateer (Agirre *et al*, 2015; Bagdonas *et al*, 2020) with CCP4 (Winn *et al*, 2011) against the unsharpened maps with the difference maps used as a guide for glycan placement and branch length. To identify potential ligand densities, fractional difference maps (Joseph *et al*, 2020) were calculated with local scaling using the TEMPy:DiffMap tool in CCPEM version 1.5.0 (Burnley *et al*, 2017). Densities were compared between a model with refined B‐factors, and the final cryo‐EM map after preprocessing (dust filter enabled at a default threshold of 0.3, and a mask around the protein). Protein Interface score (PI score; Malhotra *et al*, 2021) was used to characterize the protein–protein interaction surface for the dimer reported here, and for contacts found between molecules in the asymmetric unit of published N‐domain crystal structures. A distance cut‐off of 8 Å was used. TEMPy:SMOC (Segment‐based Manders' Overlap Coefficient) scores (Joseph *et al*, 2016) were calculated for the dimer model against the globally sharpened map reported in this study using CCPEM (Burnley *et al*, 2017), and the dimer interface SMOC score (iSMOC) calculated by averaging over the interface residues' scores.

### Normal mode analysis (NMA)

Normal mode analysis calculations of the full‐length soluble monomer structure, single monomer domains, and the 2‐domain dimer structure were performed using the WEBnm@ v2.0 server (Tiwari *et al*, 2014). A model of the full‐length soluble dimer was constructed by merging the refined 2‐domain dimer model with two copies of the refined C‐domain model and performing a bulk fitting into the low‐resolution C‐domain density from 3DVA component 0 frame 0 of the consensus 4‐domain dimer in ISOLDE (Croll, 2018) with torsion and distance restraints enabled. The proposed full‐length soluble dimer model was also subjected to NMA using the WEBnm@ v2.0 server. NMA movies for the first four low‐frequency modes with the highest collectivity were visualized.

### Pocket analysis

The surface topology of monomeric sACE^S1211^ was analyzed using the Computed Atlas of Surface Topography of Proteins (CASTp) server (Tian *et al*, 2018) with a probe radius of 1.4 Å.

### Allosteric site prediction

Potential allosteric sites were predicted for the monomeric sACE^S1211^ model using the Cavity and CorrSite2.0 modules of CavityPlus (Xu *et al*, 2018; Xie *et al*, 2022). Cavities with *Z*‐scores > 0.5 were identified as allosteric sites since their motions were highly correlated to the orthosteric site. Allosteric sites were predicted with two calculations: firstly, the N‐domain orthosteric site was defined as H361, E362, H365, E389, Q259, K489, and Y498 to identify sites, which could regulate its activity, and secondly, the C‐domain orthosteric site was defined as H959, E960, H963, E987, Q857, K1087, and Y1096 to identify sites, which could regulate its activity. The CovCys module (Xu *et al*, 2018) was used to predict covalently targetable cysteine residues within the allosteric pockets.

### Molecular docking

Molecular docking of 1,8‐ANS (1‐anilinonaphthalene‐8‐sulfonic acid) was performed using EADock at the SwissDock server (http://www.swissdock.ch/; Grosdidier *et al*, 2007) with default settings and no prior specification of the approximate binding site, i.e., blind docking was performed.

### Evolutionary conservation

Evolutionary conservation of sACE was assessed for each domain individually using the ConSurf server (Landau *et al*, 2005; Ashkenazy *et al*, 2016). ConSurf was used to generate a multiple sequence alignment using the HMMER homolog search algorithm against the UniRef‐90 database with selection of 150 sequences that sample the automatically generated list of homologs to the reference sequence. The MAFFT‐L‐INS‐I alignment method and Bayesian calculation method were used to build the multiple sequence alignment.

### Structure rendering for figures and movies

All figures and movies were created using ChimeraX version 1.2.5 (developed by the Resource for Biocomputing, Visualization, and Informatics at the University of California, San Francisco; Pettersen *et al*, 2021), Chimera version 1.15 (developed by the Resource for Biocomputing, Visualization, and Informatics at the University of California, San Francisco; Pettersen *et al*, 2004), or The PyMOL Molecular Graphics System, Version 2.4.0 Schrödinger, LLC.

## Data availability

The raw cryo‐EM micrographs, atomic coordinates, and the corresponding cryo‐EM density maps (final unsharpened maps, sharpened maps, density‐modified maps, half‐maps, and masks used for sharpening) are available from the following databases:

i Raw unaligned cryo‐EM micrographs: Electron Microscopy Public Image Archive (EMPIAR; https://www.ebi.ac.uk/empiar)
  a EMPIAR‐10980 (https://www.ebi.ac.uk/empiar/EMPIAR‐10980)
ii Atomic coordinates: PDB (https://www.ebi.ac.uk/pdbe/)
  a 7Q3Y (full‐length soluble monomer) (https://www.ebi.ac.uk/pdbe/entry/pdb/7q3y)
  b 7Q49 (monomer N‐domain) (https://www.ebi.ac.uk/pdbe/entry/pdb/7q49)
  c 7Q4C (monomer C‐domain) (https://www.ebi.ac.uk/pdbe/entry/pdb/7q4c)
  d 7Q4D (2‐domain dimer) (https://www.ebi.ac.uk/pdbe/entry/pdb/7q4d)
  e 7Q4E (single‐domain dimer) (https://www.ebi.ac.uk/pdbe/entry/pdb/7q4e)
iii Cryo‐EM maps: EMDB (https://www.ebi.ac.uk/emdb/)
  a EMD‐13797 (monomer consensus refinement) (https://www.ebi.ac.uk/emdb/EMD‐13797)
  b EMD‐13799 (monomer N‐domain local refinement) (https://www.ebi.ac.uk/emdb/EMD‐13799)
  c EMD‐13801 (monomer C‐domain local refinement) (https://www.ebi.ac.uk/emdb/EMD‐13801)
  d EMD‐13802 (dimer consensus 4‐domain refinement) (https://www.ebi.ac.uk/emdb/EMD‐13802)
  e EMD‐13803 (dimer 2‐domain local refinement) (https://www.ebi.ac.uk/emdb/EMD‐13803)
  f EMD‐13804 (dimer single‐domain local refinement) (https://www.ebi.ac.uk/emdb/EMD‐13804)

## Author contributions

**Lizelle Lubbe:** Conceptualization; data curation; formal analysis; validation; investigation; visualization; methodology; writing – original draft; writing – review and editing. **Bryan Trevor Sewell:** Resources; supervision; funding acquisition; writing – review and editing. **Jeremy D Woodward:** Resources; supervision; funding acquisition; methodology; writing – review and editing. **Edward D Sturrock:** Conceptualization; resources; supervision; funding acquisition; writing – review and editing.

In addition to the CRediT author contributions listed above, the contributions for LL and JDW in detail are:

LL purified the protein, performed biochemical analyses, prepared cryo‐EM grids, collected cryo‐EM data, performed all image processing, model building, refinement, validation, data deposition, wrote the first draft of the manuscript, and edited the manuscript. JDW provided guidance during cryo‐EM grid preparation and initial image processing in RELION, assisted with cryo‐EM data collection, and edited the manuscript.

## Disclosure and competing interests statement

The authors declare that they have no conflict of interest.

## Supporting information




Appendix

Expanded View Figures PDF

Table EV1

Movie EV1

Movie EV2

Movie EV3

Movie EV4

Movie EV5

Source Data for Figure 2

## References

[embj2021110550-bib-0001] Abrie JA , Moolman WJA , Cozier GE , Schwager SL , Acharya KR , Sturrock ED (2018) Investigation into the mechanism of homo‐ and heterodimerization of angiotensin‐converting enzyme. Mol Pharmacol 93: 344–354 29371233 10.1124/mol.117.110866

[embj2021110550-bib-0002] Afonine PV , Poon BK , Read RJ , Sobolev OV , Terwilliger TC , Urzhumtsev A , Adams PD (2018) Real‐space refinement in PHENIX for cryo‐EM and crystallography. Acta Crystallogr D Struct Biol 74: 531–544 29872004 10.1107/S2059798318006551 PMC6096492

[embj2021110550-bib-0003] Agirre J , Iglesias‐Fernández J , Rovira C , Davies GJ , Wilson KS , Cowtan KD (2015) Privateer: software for the conformational validation of carbohydrate structures. Nat Struct Mol Biol 22: 833–834 26581513 10.1038/nsmb.3115

[embj2021110550-bib-0004] Anthony CS , Corradi HR , Schwager SL , Redelinghuys P , Georgiadis D , Dive V , Acharya KR , Sturrock ED (2010) The N domain of human angiotensin‐I‐converting enzyme: the role of N‐glycosylation and the crystal structure in complex with an N domain‐specific phosphinic inhibitor, RXP407. J Biol Chem 285: 35685–35693 20826823 10.1074/jbc.M110.167866 PMC2975193

[embj2021110550-bib-0005] Asarnow D , Palovcak E , Cheng Y (2019) asarnow/pyem: UCSF pyem v0.5. Zenodo 10.5281/zenodo.3576630

[embj2021110550-bib-0006] Ashkenazy H , Abadi S , Martz E , Chay O , Mayrose I , Pupko T , Ben‐Tal N (2016) ConSurf 2016: an improved methodology to estimate and visualize evolutionary conservation in macromolecules. Nucleic Acids Res 44: W344–W350 27166375 10.1093/nar/gkw408 PMC4987940

[embj2021110550-bib-0007] Azizi M , Massien C , Michaud A , Corvol P (2000) In vitro and in vivo inhibition of the 2 active sites of ACE by omapatrilat, a vasopeptidase inhibitor. Hypertension 35: 1226–1231 10856268 10.1161/01.hyp.35.6.1226

[embj2021110550-bib-0008] Bagdonas H , Ungar D , Agirre J (2020) Leveraging glycomics data in glycoprotein 3D structure validation with privateer. Beilstein J Org Chem 16: 2523–2533 33093930 10.3762/bjoc.16.204 PMC7554661

[embj2021110550-bib-0009] Bai XC , McMullan G , Scheres SH (2015) How cryo‐EM is revolutionizing structural biology. Trends Biochem Sci 40: 49–57 25544475 10.1016/j.tibs.2014.10.005

[embj2021110550-bib-0010] Barad BA , Echols N , Wang RY , Cheng Y , DiMaio F , Adams PD , Fraser JS (2015) EMRinger: side chain‐directed model and map validation for 3D cryo‐electron microscopy. Nat Methods 12: 943–946 26280328 10.1038/nmeth.3541 PMC4589481

[embj2021110550-bib-0011] Barauna VG , Campos LC , Miyakawa AA , Krieger JE (2011) ACE as a mechanosensor to shear stress influences the control of its own regulation via phosphorylation of cytoplasmic Ser(1270). PLoS ONE 6: e22803 21901117 10.1371/journal.pone.0022803 PMC3161988

[embj2021110550-bib-0012] Bepler T , Kelley K , Noble AJ , Berger B (2020) Topaz‐Denoise: general deep denoising models for cryoEM and cryoET. Nat Commun 11: 5208 33060581 10.1038/s41467-020-18952-1 PMC7567117

[embj2021110550-bib-0013] Bepler T , Morin A , Rapp M , Brasch J , Shapiro L , Noble AJ , Berger B (2019) Positive‐unlabeled convolutional neural networks for particle picking in cryo‐electron micrographs. Nat Methods 16: 1153–1160 31591578 10.1038/s41592-019-0575-8 PMC6858545

[embj2021110550-bib-0014] Bernstein KE , Ong FS , Blackwell W‐LB , Shah KH , Giani JF , Gonzalez‐Villalobos RA , Shen XZ , Fuchs S (2013) A modern understanding of the traditional and nontraditional biological functions of angiotensin‐converting enzyme. Pharmacol Rev 65: 1–46 23257181 10.1124/pr.112.006809 PMC3565918

[embj2021110550-bib-0015] Bicket DP (2002) Using ACE inhibitors appropriately. Am Fam Physician 66: 461–468 12182524

[embj2021110550-bib-0016] Binevski PV , Sizova EA , Pozdnev VF , Kost OA (2003) Evidence for the negative cooperativity of the two active sites within bovine somatic angiotensin‐converting enzyme. FEBS Lett 550: 84–88 12935891 10.1016/s0014-5793(03)00825-1

[embj2021110550-bib-0017] Boginskaya I , Nechaeva N , Tikhomirova V , Kryukova O , Evdokimov V , Bulaeva N , Golukhova E , Ryzhikov I , Kost O , Afanasev K *et al* (2021) Human angiotensin I‐converting enzyme study by surface‐enhanced Raman spectroscopy. J Raman Spectrosc 52: 1529–1539

[embj2021110550-bib-0018] Brás NF , Fernandes PA , Ramos MJ (2014) QM/MM study and MD simulations on the hypertension regulator angiotensin‐converting enzyme. ACS Catalysis 4: 2587–2597

[embj2021110550-bib-0019] Bull HG , Thornberry NA , Cordes EH (1985) Purification of angiotensin‐converting enzyme from rabbit lung and human plasma by affinity chromatography. J Biol Chem 260: 2963–2972 2982846

[embj2021110550-bib-0020] Burnley T , Palmer CM , Winn M (2017) Recent developments in the CCP‐EM software suite. Acta Crystallogr D Struct Biol 73: 469–477 28580908 10.1107/S2059798317007859 PMC5458488

[embj2021110550-bib-0021] Cao D‐Y , Spivia WR , Veiras LC , Khan Z , Peng Z , Jones AE , Bernstein EA , Saito S , Okwan‐Duodu D , Parker SJ *et al* (2020) ACE overexpression in myeloid cells increases oxidative metabolism and cellular ATP. J Biol Chem 295: 1369–1384 31871049 10.1074/jbc.RA119.011244 PMC6996878

[embj2021110550-bib-0022] Cao D , Veiras L , Ahmed F , Shibata T , Bernstein EA , Okwan‐Duodu D , Giani JF , Khan Z , Bernstein KE (2022) The non‐cardiovascular actions of ACE. Peptides 152: 170769 35182689 10.1016/j.peptides.2022.170769 PMC10405936

[embj2021110550-bib-0023] Chen HL , Lunsdorf H , Hecht HJ , Tsai H (2010) Porcine pulmonary angiotensin I‐converting enzyme—Biochemical characterization and spatial arrangement of the N‐ and C‐domains by three‐dimensional electron microscopic reconstruction. Micron 41: 674–685 20427191 10.1016/j.micron.2010.01.005

[embj2021110550-bib-0024] Comellas‐Bigler M , Lang R , Bode W , Maskos K (2005) Crystal structure of the E.coli dipeptidyl carboxypeptidase Dcp: further indication of a ligand‐dependant hinge movement mechanism. J Mol Biol 349: 99–112 15876371 10.1016/j.jmb.2005.03.016

[embj2021110550-bib-0025] Cozier GE , Arendse LB , Schwager SL , Sturrock ED , Acharya KR (2018) Molecular basis for multiple omapatrilat binding sites within the ACE C‐domain: implications for drug design. J Med Chem 61: 10141–10154 30372620 10.1021/acs.jmedchem.8b01309

[embj2021110550-bib-0026] Cozier GE , Lubbe L , Sturrock ED , Acharya KR (2020a) ACE‐domain selectivity extends beyond direct interacting residues at the active site. Biochem J 477: 1241–1259 32195541 10.1042/BCJ20200060 PMC7148434

[embj2021110550-bib-0027] Cozier GE , Lubbe L , Sturrock ED , Acharya KR (2020b) Angiotensin‐converting enzyme open for business: structural insights into the sub‐domain dynamics. FEBS J 288: 2238–2256 33067882 10.1111/febs.15601 PMC8048788

[embj2021110550-bib-0028] Croll TI (2018) ISOLDE: a physically realistic environment for model building into low‐resolution electron‐density maps. Acta Crystallogr D Struct Biol 74: 519–530 29872003 10.1107/S2059798318002425 PMC6096486

[embj2021110550-bib-0029] Croll TI , Read RJ (2021) Adaptive cartesian and torsional restraints for interactive model rebuilding. Acta Crystallogr D Struct Biol 77: 438–446 33825704 10.1107/S2059798321001145 PMC8025879

[embj2021110550-bib-0030] Danilov SM (2017) Conformational fingerprinting using monoclonal antibodies (on the example of angiotensin I‐converting enzyme‐ACE). Mol Biol 51: 906–920 32287393 10.1134/S0026893317060048 PMC7102274

[embj2021110550-bib-0031] Danilov SM , Balyasnikova IV , Danilova AS , Naperova IA , Arablinskaya NE , Borisov SE , Metzger R , Franke FE , Schwartz DE , Gachok IV *et al* (2010) Conformational fingerprinting of the angiotensin I‐converting enzyme (ACE). 1. Application in sarcoidosis. J Proteome Res 9: 5782–5793 20873814 10.1021/pr100564r

[embj2021110550-bib-0032] Danilov SM , Gordon K , Nesterovitch AB , Lünsdorf H , Chen Z , Castellon M , Popova IA , Kalinin S , Mendonca E , Petukhov PA *et al* (2011) An angiotensin I‐converting enzyme mutation (Y465D) causes a dramatic increase in blood ACE via accelerated ACE shedding. PLoS ONE 6: e25952 21998728 10.1371/journal.pone.0025952 PMC3187827

[embj2021110550-bib-0033] Danilov SM , Jain MS , Petukhov PA , Goldman C , DiSanto‐Rose M , Vancavage R , Francuzevitch LY , Samokhodskaya LM , Kamalov AA , Arbieva ZH *et al* (2021) Novel ACE mutations mimicking sarcoidosis by increasing blood ACE levels. Transl Res 230: 5–20 32726712 10.1016/j.trsl.2020.07.010

[embj2021110550-bib-0034] Danilov SM , Tikhomirova VE , Metzger R , Naperova IA , Bukina TM , Goker‐Alpan O , Tayebi N , Gayfullin NM , Schwartz DE , Samokhodskaya LM *et al* (2018) ACE phenotyping in Gaucher disease. Mol Genet Metab 123: 501–510 29478818 10.1016/j.ymgme.2018.02.007 PMC5891352

[embj2021110550-bib-0035] Donoghue M , Hsieh F , Baronas E , Godbout K , Gosselin M , Stagliano N , Donovan M , Woolf B , Robison K , Jeyaseelan R *et al* (2000) A novel angiotensin‐converting enzyme‐related carboxypeptidase (ACE2) converts angiotensin I to angiotensin 1‐9. Circ Res 87: e1–e9 10969042 10.1161/01.res.87.5.e1

[embj2021110550-bib-0036] Echols N , Grosse‐Kunstleve RW , Afonine PV , Bunkóczi G , Chen VB , Headd JJ , McCoy AJ , Moriarty NW , Read RJ , Richardson DC *et al* (2012) Graphical tools for macromolecular crystallography in PHENIX. J Appl Cryst 45: 581–586 22675231 10.1107/S0021889812017293 PMC3359726

[embj2021110550-bib-0037] Egelman EH (2016) The current revolution in Cryo‐EM. Biophys J 110: 1008–1012 26958874 10.1016/j.bpj.2016.02.001 PMC4788751

[embj2021110550-bib-0038] Ehlers MR , Abrie JA , Sturrock ED (2013) C domain‐selective inhibition of angiotensin‐converting enzyme. J Renin Angiotensin Aldosterone Syst 14: 189–192 23686914 10.1177/1470320313489206

[embj2021110550-bib-0039] Ehlers MR , Gordon K , Schwager SL , Sturrock ED (2012) Shedding the load of hypertension: The proteolytic processing of angiotensin‐converting enzyme. S Afr Med J 102: 461–464 22668937 10.7196/samj.5596

[embj2021110550-bib-0040] Ehlers MRW , Riordan JF (1989) Angiotensin‐converting enzyme: new concepts concerning its biological role. Biochemistry 28: 5311–5318 2476171 10.1021/bi00439a001

[embj2021110550-bib-0041] Ehlers MRW , Riordan JF (1991) Angiotensin‐converting enzyme: Zinc‐ and inhibitor‐binding stoichiometries of the somatic and testis isozymes. Biochemistry 30: 7118–7126 1649623 10.1021/bi00243a012

[embj2021110550-bib-0042] Ehlers MRW , Schwager SLU , Scholle RR , Manji GA , Brandt WF , Riordan JF (1996) Proteolytic release of membrane‐bound angiotensin‐converting enzyme: Role of the Juxtamembrane stalk sequence. Biochemistry 35: 9549–9559 8755736 10.1021/bi9602425

[embj2021110550-bib-0043] English WR , Corvol P , Murphy G (2012) LPS activates ADAM9 dependent shedding of ACE from endothelial cells. Biochem Biophys Res Commun 421: 70–75 22480688 10.1016/j.bbrc.2012.03.113

[embj2021110550-bib-0044] Fleming I (2006) Signaling by the angiotensin‐converting enzyme. Circ Res 98: 887–896 16614314 10.1161/01.RES.0000217340.40936.53

[embj2021110550-bib-0045] Gordon K , Balyasnikova IV , Nesterovitch AB , Schwartz DE , Sturrock ED , Danilov SM (2010) Fine epitope mapping of monoclonal antibodies 9B9 and 3G8 to the N domain of angiotensin‐converting enzyme (CD143) defines a region involved in regulating angiotensin‐converting enzyme dimerization and shedding. Tissue Antigens 75: 136–150 20003136 10.1111/j.1399-0039.2009.01416.x

[embj2021110550-bib-0046] Gordon K , Redelinghuys P , Schwager SL , Ehlers MR , Papageorgiou AC , Natesh R , Acharya KR , Sturrock ED (2003) Deglycosylation, processing and crystallization of human testis angiotensin‐converting enzyme. Biochem J 371: 437–442 12542396 10.1042/BJ20021842 PMC1223310

[embj2021110550-bib-0047] Grosdidier A , Zoete V , Michielin O (2007) EADock: docking of small molecules into protein active sites with a multiobjective evolutionary optimization. Proteins 67: 1010–1025 17380512 10.1002/prot.21367

[embj2021110550-bib-0048] Herzik MA , Wu M , Lander GC (2019) High‐resolution structure determination of sub‐100 kDa complexes using conventional cryo‐EM. Nat Commun 10: 1032 30833564 10.1038/s41467-019-08991-8 PMC6399227

[embj2021110550-bib-0049] Hines CS , Ray K , Schmidt JJ , Xiong F , Feenstra RW , Pras‐Raves M , de Moes JP , Lange JH , Melikishvili M , Fried MG *et al* (2014) Allosteric inhibition of the neuropeptidase neurolysin. J Biol Chem 289: 35605–35619 25378390 10.1074/jbc.M114.620930 PMC4271243

[embj2021110550-bib-0050] Hoffmann M , Kleine‐Weber H , Schroeder S , Krüger N , Herrler T , Erichsen S , Schiergens TS , Herrler G , Wu N‐H , Nitsche A *et al* (2020) SARS‐CoV‐2 cell entry depends on ACE2 and TMPRSS2 and is blocked by a clinically proven protease inhibitor. Cell 181: 271–280 32142651 10.1016/j.cell.2020.02.052 PMC7102627

[embj2021110550-bib-0051] Huang W , Ravikumar KM , Chance MR , Yang S (2015) Quantitative mapping of protein structure by hydroxyl radical footprinting‐mediated structural mass spectrometry: a protection factor analysis. Biophys J 108: 107–115 25564857 10.1016/j.bpj.2014.11.013 PMC4286602

[embj2021110550-bib-0052] Jaspard E , Alhenc‐Gelas F (1995) Catalytic properties of the two active sites of angiotensin I‐converting enzyme on the cell surface. Biochem Biophys Res Commun 211: 528–534 7794265 10.1006/bbrc.1995.1845

[embj2021110550-bib-0053] Joseph AP , Lagerstedt I , Jakobi A , Burnley T , Patwardhan A , Topf M , Winn M (2020) Comparing Cryo‐EM reconstructions and validating atomic model fit using difference maps. J Chem Inf Model 60: 2552–2560 32043355 10.1021/acs.jcim.9b01103 PMC7254831

[embj2021110550-bib-0054] Joseph AP , Malhotra S , Burnley T , Wood C , Clare DK , Winn M , Topf M (2016) Refinement of atomic models in high resolution EM reconstructions using flex‐EM and local assessment. Methods 100: 42–49 26988127 10.1016/j.ymeth.2016.03.007 PMC4854230

[embj2021110550-bib-0055] Jumper J , Evans R , Pritzel A , Green T , Figurnov M , Ronneberger O , Tunyasuvunakool K , Bates R , Žídek A , Potapenko A *et al* (2021) Highly accurate protein structure prediction with AlphaFold. Nature 596: 583–589 34265844 10.1038/s41586-021-03819-2 PMC8371605

[embj2021110550-bib-0056] Junot C , Gonzales MF , Ezan E , Cotton J , Vazeux G , Michaud A , Azizi M , Vassiliou S , Yiotakis A , Corvol P *et al* (2001) RXP 407, a selective inhibitor of the N‐domain of angiotensin I‐converting enzyme, blocks in vivo the degradation of hemoregulatory peptide acetyl‐Ser‐Asp‐Lys‐Pro with no effect on angiotensin I hydrolysis. J Pharmacol Exp Ther 297: 606–611 11303049

[embj2021110550-bib-0057] Kohlstedt K , Brandes Ralf P , Müller‐Esterl W , Busse R , Fleming I (2004) Angiotensin‐converting enzyme is involved in outside‐in signaling in endothelial cells. Circ Res 94: 60–67 14615289 10.1161/01.RES.0000107195.13573.E4

[embj2021110550-bib-0058] Kohlstedt K , Gershome C , Friedrich M , Muller‐Esterl W , Alhenc‐Gelas F , Busse R , Fleming I (2006) Angiotensin‐converting enzyme (ACE) dimerization is the initial step in the ACE inhibitor‐induced ACE signaling cascade in endothelial cells. Mol Pharmacol 69: 1725–1732 16476786 10.1124/mol.105.020636

[embj2021110550-bib-0059] Kohlstedt K , Shoghi F , Müller‐Esterl W , Busse R , Fleming I (2002) CK2 phosphorylates the angiotensin‐converting enzyme and regulates its retention in the endothelial cell plasma membrane. Circ Res 91: 749–756 12386153 10.1161/01.res.0000038114.17939.c8

[embj2021110550-bib-0060] Kost OA , Balyasnikova IV , Chemodanova EE , Nikolskaya II , Albrecht RF 2nd , Danilov SM (2003) Epitope‐dependent blocking of the angiotensin‐converting enzyme dimerization by monoclonal antibodies to the N‐terminal domain of ACE: Possible link of ACE dimerization and shedding from the cell surface. Biochemistry 42: 6965–6976 12795591 10.1021/bi034645y

[embj2021110550-bib-0061] Kost OA , Bovin NV , Chemodanova EE , Nasonov VV , Orth TA (2000) New feature of angiotensin‐converting enzyme: carbohydrate‐recognizing domain. J Mol Recognit 13: 360–369 11114069 10.1002/1099-1352(200011/12)13:6<360::AID-JMR508>3.0.CO;2-K

[embj2021110550-bib-0062] Kostis WJ , Shetty M , Chowdhury YS , Kostis JB (2018) ACE inhibitor‐induced angioedema: a review. Curr Hypertens Rep 20: 55 29884969 10.1007/s11906-018-0859-x

[embj2021110550-bib-0063] Kroger WL , Douglas RG , O'Neill HG , Dive V , Sturrock ED (2009) Investigating the domain specificity of phosphinic inhibitors RXPA380 and RXP407 in angiotensin‐converting enzyme. Biochemistry 48: 8405–8412 19658433 10.1021/bi9011226

[embj2021110550-bib-0064] Kryukova OV , Tikhomirova VE , Golukhova EZ , Evdokimov VV , Kalantarov GF , Trakht IN , Schwartz DE , Dull RO , Gusakov AV , Uporov IV *et al* (2015) Tissue specificity of human angiotensin I‐converting enzyme. PLoS ONE 10: e0143455 26600189 10.1371/journal.pone.0143455 PMC4658169

[embj2021110550-bib-0065] Landau M , Mayrose I , Rosenberg Y , Glaser F , Martz E , Pupko T , Ben‐Tal N (2005) ConSurf 2005: the projection of evolutionary conservation scores of residues on protein structures. Nucleic Acids Res 33: W299–W302 15980475 10.1093/nar/gki370 PMC1160131

[embj2021110550-bib-0066] Lanzillo JJ , Polsky‐Cynkin R , Fanburg BL (1980) Large‐scale purification of angiotensin I‐converting enzyme from human plasma utilizing an immunoadsorbent affinity gel. Anal Biochem 103: 400–407 6247934 10.1016/0003-2697(80)90630-2

[embj2021110550-bib-0067] Larmuth KM , Masuyer G , Douglas RG , Schwager SL , Acharya KR , Sturrock ED (2016) Kinetic and structural characterization of amyloid‐beta peptide hydrolysis by human angiotensin‐1‐converting enzyme. FEBS J 283: 1060–1076 26748546 10.1111/febs.13647 PMC4950319

[embj2021110550-bib-0068] Le D , Brown L , Malik K , Murakami S (2021) Two opposing functions of angiotensin‐converting enzyme (ACE) that links hypertension, dementia, and aging. Int J Mol Sci 22: 13178 34947975 10.3390/ijms222413178 PMC8707689

[embj2021110550-bib-0069] Lee AF , MacFadyen RJ , Struthers AD (1999) Neurohormonal reactivation in heart failure patients on chronic ACE inhibitor therapy: a longitudinal study. Eur J Heart Fail 1: 401–406 10937954 10.1016/s1388-9842(99)00046-x

[embj2021110550-bib-0070] Li W , Moore MJ , Vasilieva N , Sui J , Wong SK , Berne MA , Somasundaran M , Sullivan JL , Luzuriaga K , Greenough TC *et al* (2003) Angiotensin‐converting enzyme 2 is a functional receptor for the SARS coronavirus. Nature 426: 450–454 14647384 10.1038/nature02145 PMC7095016

[embj2021110550-bib-0071] Lubbe L , Sewell BT , Sturrock ED (2016) The influence of angiotensin converting enzyme mutations on the kinetics and dynamics of N‐domain selective inhibition. FEBS J 283: 3941–3961 27636235 10.1111/febs.13900

[embj2021110550-bib-0072] Ma X , Meng H , Lai L (2016) Motions of allosteric and Orthosteric ligand‐binding sites in proteins are highly correlated. J Chem Inf Model 56: 1725–1733 27580047 10.1021/acs.jcim.6b00039

[embj2021110550-bib-0073] Malhotra S , Joseph AP , Thiyagalingam J , Topf M (2021) Assessment of protein‐protein interfaces in cryo‐EM derived assemblies. Nat Commun 12: 3399 34099703 10.1038/s41467-021-23692-x PMC8184972

[embj2021110550-bib-0074] Masuyer G , Akif M , Czarny B , Beau F , Schwager SLU , Sturrock ED , Isaac RE , Dive V , Acharya KR (2014a) Crystal structures of highly specific phosphinic tripeptide enantiomers in complex with the angiotensin‐I converting enzyme. FEBS J 281: 943–956 24289879 10.1111/febs.12660 PMC4154125

[embj2021110550-bib-0075] Masuyer G , Douglas RG , Sturrock ED , Acharya KR (2015) Structural basis of Ac‐SDKP hydrolysis by angiotensin‐I converting enzyme. Sci Rep 5: 13742 26403559 10.1038/srep13742 PMC4585900

[embj2021110550-bib-0076] Masuyer G , Schwager SL , Sturrock ED , Isaac RE , Acharya KR (2012) Molecular recognition and regulation of human angiotensin‐I converting enzyme (ACE) activity by natural inhibitory peptides. Sci Rep 2: 717 23056909 10.1038/srep00717 PMC3466449

[embj2021110550-bib-0077] Masuyer G , Yates CJ , Sturrock ED , Acharya KR (2014b) Angiotensin‐I converting enzyme (ACE): Structure, biological roles, and molecular basis for chloride ion dependence. Biol Chem 395: 1135–1149 25205727 10.1515/hsz-2014-0157

[embj2021110550-bib-0078] Michaud A , Acharya KR , Masuyer G , Quenech'du N , Gribouval O , Morinière V , Gubler MC , Corvol P (2014) Absence of cell surface expression of human ACE leads to perinatal death. Hum Mol Genet 23: 1479–1491 24163131 10.1093/hmg/ddt535 PMC3929087

[embj2021110550-bib-0079] Mu X , Zhang C , Xu D (2016) QM/MM investigation of the catalytic mechanism of angiotensin‐converting enzyme. J Mol Model 22: 132 27184002 10.1007/s00894-016-3004-2

[embj2021110550-bib-0080] Naqvi N , Liu K , Graham RM , Husain A (2005) Molecular basis of exopeptidase activity in the C‐terminal domain of human angiotensin I‐converting enzyme: insights into the origins of its exopeptidase activity. J Biol Chem 280: 6669–6675 15615692 10.1074/jbc.M412638200

[embj2021110550-bib-0081] O'Neill HG , Redelinghuys P , Schwager SL , Sturrock ED (2008) The role of glycosylation and domain interactions in the thermal stability of human angiotensin‐converting enzyme. Biol Chem 389: 1153–1161 18713002 10.1515/BC.2008.131

[embj2021110550-bib-0082] Oba R , Igarashi A , Kamata M , Nagata K , Takano S , Nakagawa H (2005) The N‐terminal active Centre of human angiotensin‐converting enzyme degrades Alzheimer amyloid beta‐peptide. Eur J Neurosci 21: 733–740 15733091 10.1111/j.1460-9568.2005.03912.x

[embj2021110550-bib-0083] Ondetti MA , Rubin B , Cushman DW (1977) Design of specific inhibitors of angiotensin‐converting enzyme: new class of orally active antihypertensive agents. Science 196: 441–444 191908 10.1126/science.191908

[embj2021110550-bib-0084] Orlova EV , Saibil HR (2011) Structural analysis of macromolecular assemblies by electron microscopy. Chem Rev 111: 7710–7748 21919528 10.1021/cr100353t PMC3239172

[embj2021110550-bib-0085] Pettersen EF , Goddard TD , Huang CC , Couch GS , Greenblatt DM , Meng EC , Ferrin TE (2004) UCSF chimera‐‐a visualization system for exploratory research and analysis. J Comput Chem 25: 1605–1612 15264254 10.1002/jcc.20084

[embj2021110550-bib-0086] Pettersen EF , Goddard TD , Huang CC , Meng EC , Couch GS , Croll TI , Morris JH , Ferrin TE (2021) UCSF ChimeraX: Structure visualization for researchers, educators, and developers. Protein Sci 30: 70–82 32881101 10.1002/pro.3943 PMC7737788

[embj2021110550-bib-0087] Popova IA , Lubbe L , Petukhov PA , Kalantarov GF , Trakht IN , Chernykh ER , Leplina OY , Lyubimov AV , Garcia JGN , Dudek SM *et al* (2021) Epitope mapping of novel monoclonal antibodies to human angiotensin I‐converting enzyme. Protein Sci 30: 1577–1593 33931897 10.1002/pro.4091 PMC8284578

[embj2021110550-bib-0088] Punjani A , Fleet DJ (2021) 3D variability analysis: Resolving continuous flexibility and discrete heterogeneity from single particle cryo‐EM. J Struct Biol 213: 107702 33582281 10.1016/j.jsb.2021.107702

[embj2021110550-bib-0089] Punjani A , Rubinstein JL , Fleet DJ , Brubaker MA (2017) cryoSPARC: Algorithms for rapid unsupervised cryo‐EM structure determination. Nat Methods 14: 290–296 28165473 10.1038/nmeth.4169

[embj2021110550-bib-0090] Punjani A , Zhang H , Fleet DJ (2020) Non‐uniform refinement: Adaptive regularization improves single‐particle cryo‐EM reconstruction. Nat Methods 17: 1214–1221 33257830 10.1038/s41592-020-00990-8

[embj2021110550-bib-0091] Ray K , Hines CS , Coll‐Rodriguez J , Rodgers DW (2004) Crystal structure of human thimet oligopeptidase provides insight into substrate recognition, regulation, and localization. J Biol Chem 279: 20480–20489 14998993 10.1074/jbc.M400795200

[embj2021110550-bib-0092] Redelinghuys P (2006) Structure‐function relationship of angiotensin‐converting enzyme: glycosylation and domain‐selectivity. Cape Town: University of Cape Town

[embj2021110550-bib-0093] Reis RI , Nogueira MD , Campanha‐Rodrigues AL , Pereira LM , Andrade MCC , Parreiras‐e‐Silva LT , Costa‐Neto CM , Mortara RA , Casarini DE (2018) The binding of captopril to angiotensin I‐converting enzyme triggers activation of signaling pathways. Am J Physiol Cell Physiol 315: C367–C379 29874111 10.1152/ajpcell.00012.2016

[embj2021110550-bib-0094] Rice GI , Thomas DA , Grant PJ , Turner AJ , Hooper NM (2004) Evaluation of angiotensin‐converting enzyme (ACE), its homologue ACE2 and neprilysin in angiotensin peptide metabolism. Biochem J 383: 45–51 15283675 10.1042/BJ20040634 PMC1134042

[embj2021110550-bib-0095] Rohou A , Grigorieff N (2015) CTFFIND4: Fast and accurate defocus estimation from electron micrographs. J Struct Biol 192: 216–221 26278980 10.1016/j.jsb.2015.08.008 PMC6760662

[embj2021110550-bib-0096] Rousseau A , Michaud A , Chauvet MT , Lenfant M , Corvol P (1995) The hemoregulatory peptide N‐acetyl‐Ser‐Asp‐Lys‐Pro is a natural and specific substrate of the N‐terminal active site of human angiotensin‐converting enzyme. J Biol Chem 270: 3656–3661 7876104 10.1074/jbc.270.8.3656

[embj2021110550-bib-0097] Sassi C , Ridge PG , Nalls MA , Gibbs R , Ding J , Lupton MK , Troakes C , Lunnon K , Al‐Sarraj S , Brown KS *et al* (2016) Influence of coding variability in APP‐Aβ metabolism genes in sporadic Alzheimer's disease. PLoS ONE 11: e0150079 27249223 10.1371/journal.pone.0150079 PMC4889076

[embj2021110550-bib-0098] Scheres SH (2012) RELION: implementation of a Bayesian approach to cryo‐EM structure determination. J Struct Biol 180: 519–530 23000701 10.1016/j.jsb.2012.09.006 PMC3690530

[embj2021110550-bib-0099] Scheres SHW (2016) Chapter six – processing of structurally heterogeneous Cryo‐EM data in RELION. In Methods in Enzymology, RA Crowther (ed), pp 125–157. Cambridge, MA: Academic Press 10.1016/bs.mie.2016.04.012 27572726

[embj2021110550-bib-0100] Schwager SL , Carmona AK , Sturrock ED (2006) A high‐throughput fluorimetric assay for angiotensin I‐converting enzyme. Nat Protoc 1: 1961–1964 17487183 10.1038/nprot.2006.305

[embj2021110550-bib-0101] Skirgello OE , Balyasnikova IV , Binevski PV , Sun ZL , Baskin II , Palyulin VA , Nesterovitch AB , Albrecht RF 2nd , Kost OA , Danilov SM (2006) Inhibitory antibodies to human angiotensin‐converting enzyme: fine epitope mapping and mechanism of action. Biochemistry 45: 4831–4847 16605251 10.1021/bi052591h

[embj2021110550-bib-0102] Skirgello OE , Binevski PV , Pozdnev VF , Kost OA (2005) Kinetic probes for inter‐domain co‐operation in human somatic angiotensin‐converting enzyme. Biochem J 391: 641–647 16033330 10.1042/BJ20050702 PMC1276965

[embj2021110550-bib-0103] Stapff M , Hilderbrand S (2019) First‐line treatment of essential hypertension: a real‐world analysis across four antihypertensive treatment classes. J Clin Hypertens (Greenwich) 21: 627–634 30980608 10.1111/jch.13531 PMC8030363

[embj2021110550-bib-0104] Sturrock ED , Lubbe L , Cozier GE , Schwager SLU , Arowolo AT , Arendse LB , Belcher E , Acharya KR (2019) Structural basis for the C‐domain‐selective angiotensin‐converting enzyme inhibition by bradykinin‐potentiating peptide b (BPPb). Biochem J 476: 1553–1570 31072910 10.1042/BCJ20190290

[embj2021110550-bib-0105] Sun X , Rentzsch B , Gong M , Eichhorst J , Pankow K , Papsdorf G , Maul B , Bader M , Siems W‐E (2010) Signal transduction in CHO cells stably transfected with domain‐selective forms of murine ACE. Biol Chem 391: 235–244 20030584 10.1515/bc.2010.020

[embj2021110550-bib-0106] Tan YZ , Baldwin PR , Davis JH , Williamson JR , Potter CS , Carragher B , Lyumkis D (2017) Addressing preferred specimen orientation in single‐particle cryo‐EM through tilting. Nat Methods 14: 793–796 28671674 10.1038/nmeth.4347 PMC5533649

[embj2021110550-bib-0107] Teixeira PF , Masuyer G , Pinho CM , Branca RMM , Kmiec B , Wallin C , Wärmländer SKTS , Berntsson RPA , Ankarcrona M , Gräslund A *et al* (2018) Mechanism of peptide binding and cleavage by the human mitochondrial peptidase Neurolysin. J Mol Biol 430: 348–362 29183787 10.1016/j.jmb.2017.11.011

[embj2021110550-bib-0108] Terwilliger TC , Ludtke SJ , Read RJ , Adams PD , Afonine PV (2020) Improvement of cryo‐EM maps by density modification. Nat Methods 17: 923–927 32807957 10.1038/s41592-020-0914-9 PMC7484085

[embj2021110550-bib-0109] Tian W , Chen C , Lei X , Zhao J , Liang J (2018) CASTp 3.0: computed atlas of surface topography of proteins. Nucleic Acids Res 46: W363–W367 29860391 10.1093/nar/gky473 PMC6031066

[embj2021110550-bib-0110] Tiwari SP , Fuglebakk E , Hollup SM , Skjærven L , Cragnolini T , Grindhaug SH , Tekle KM , Reuter N (2014) WEBnm@ v2.0: Web server and services for comparing protein flexibility. BMC Bioinformatics 15: 427 25547242 10.1186/s12859-014-0427-6 PMC4339738

[embj2021110550-bib-0111] Towler P , Staker B , Prasad SG , Menon S , Tang J , Parsons T , Ryan D , Fisher M , Williams D , Dales NA *et al* (2004) ACE2 X‐ray structures reveal a large hinge‐bending motion important for inhibitor binding and catalysis. J Biol Chem 279: 17996–18007 14754895 10.1074/jbc.M311191200 PMC7980034

[embj2021110550-bib-0112] Turner AJ , Nalivaeva NN (2022) Angiotensin‐converting enzyme 2 (ACE2): two decades of revelations and re‐evaluation. Peptides 151: 170766 35151768 10.1016/j.peptides.2022.170766 PMC8830188

[embj2021110550-bib-0113] Voronov SV , Binevski PV , Zueva NA , Palyulin VA , Baskin II , Orlova MA , Kost OA (2003) Structural and functional peculiarities of homologous domains of angiotensin‐converting enzyme. Russ J Bioorganic Chem 29: 426–433 10.1023/a:1026045324442 14601401

[embj2021110550-bib-0114] Voronov SV , Skirgello OE , Troshina NN , Orlova MA , Kost OA (2002) A hydrophobic site on the surface of the angiotensin‐converting enzyme molecule. Biochemistry (Mosc) 67: 553–557 12059775 10.1023/a:1015598228545

[embj2021110550-bib-0115] Vy TT , Heo SY , Jung WK , Yi M (2020) Spontaneous hinge‐bending motions of angiotensin I converting enzyme: role in activation and inhibition. Molecules 25: 1288 10.3390/molecules25061288 PMC7146279 32178362

[embj2021110550-bib-0116] Wang X , Wu S , Xu D , Xie D , Guo H (2011) Inhibitor and substrate binding by angiotensin‐converting enzyme: quantum mechanical/molecular mechanical molecular dynamics studies. J Chem Inf Model 51: 1074–1082 21520937 10.1021/ci200083f PMC3156973

[embj2021110550-bib-0117] Watermeyer JM , Sewell BT , Schwager SL , Natesh R , Corradi HR , Acharya KR , Sturrock ED (2006) Structure of testis ACE glycosylation mutants and evidence for conserved domain movement. Biochemistry 45: 12654–12663 17042482 10.1021/bi061146z PMC1892614

[embj2021110550-bib-0118] Wei L (1991) The two homologous domains of human angiotensin I‐converting enzyme are both catalytically active. J Biol Chem 266: 9002–9008 1851160

[embj2021110550-bib-0119] Wei L , Clauser E , Alhenc‐Gelas F , Corvol P (1992) The two homologous domains of human angiotensin I‐converting enzyme interact differently with competitive inhibitors. J Biol Chem 267: 13398–13405 1320019

[embj2021110550-bib-0120] Winn MD , Ballard CC , Cowtan KD , Dodson EJ , Emsley P , Evans PR , Keegan RM , Krissinel EB , Leslie AG , McCoy A *et al* (2011) Overview of the CCP4 suite and current developments. Acta Crystallogr D Biol Crystallogr 67: 235–242 21460441 10.1107/S0907444910045749 PMC3069738

[embj2021110550-bib-0121] Xie J , Wang S , Xu Y , Deng M , Lai L (2022) Uncovering the dominant motion modes of allosteric regulation improves allosteric site prediction. J Chem Inf Model 62: 187–195 34964625 10.1021/acs.jcim.1c01267

[embj2021110550-bib-0122] Xu Y , Wang S , Hu Q , Gao S , Ma X , Zhang W , Shen Y , Chen F , Lai L , Pei J (2018) CavityPlus: a web server for protein cavity detection with pharmacophore modelling, allosteric site identification and covalent ligand binding ability prediction. Nucleic Acids Res 46: W374–W379 29750256 10.1093/nar/gky380 PMC6031032

[embj2021110550-bib-0123] Yates CJ , Masuyer G , Schwager SL , Akif M , Sturrock ED , Acharya KR (2014) Molecular and thermodynamic mechanisms of the chloride‐dependent human angiotensin‐I‐converting enzyme (ACE). J Biol Chem 289: 1798–1814 24297181 10.1074/jbc.M113.512335 PMC3894356

[embj2021110550-bib-0124] Yu XC , Sturrock ED , Wu Z , Biemann K , Ehlers MR , Riordan JF (1997) Identification of N‐linked glycosylation sites in human testis angiotensin‐converting enzyme and expression of an active deglycosylated form. J Biol Chem 272: 3511–3519 9013598 10.1074/jbc.272.6.3511

[embj2021110550-bib-0125] Zhang C , Wu S , Xu D (2013) Catalytic mechanism of angiotensin‐converting enzyme and effects of the chloride ion. J Phys Chem B 117: 6635–6645 23672666 10.1021/jp400974n

[embj2021110550-bib-0126] Zheng SQ , Palovcak E , Armache JP , Verba KA , Cheng Y , Agard DA (2017) MotionCor2: anisotropic correction of beam‐induced motion for improved cryo‐electron microscopy. Nat Methods 14: 331–332 28250466 10.1038/nmeth.4193 PMC5494038

[embj2021110550-bib-0127] Zivanov J , Nakane T , Forsberg BO , Kimanius D , Hagen WJ , Lindahl E , Scheres SH (2018) New tools for automated high‐resolution cryo‐EM structure determination in RELION‐3. eLife 7: e42166 30412051 10.7554/eLife.42166 PMC6250425

[embj2021110550-bib-0128] Zou K , Maeda T , Watanabe A , Liu J , Liu S , Oba R , Satoh Y , Komano H , Michikawa M (2009) Abeta42‐to‐Abeta40‐ and angiotensin‐converting activities in different domains of angiotensin‐converting enzyme. J Biol Chem 284: 31914–31920 19773553 10.1074/jbc.M109.011437 PMC2797262

