# Supplementary material for: Cryo‐EM reveals mechanisms of angiotensin I‐converting enzyme allostery and dimerization
Source: EMBO J. 2022 Jul 12;41(16):e110550. doi: 10.15252/embj.2021110550 (PMC9379546; doi:10.15252/embj.2021110550)
Supplement: Supplementary file 1 — Appendix [file EMBJ-41-e110550-s005.pdf]

Appendix for manuscript

Cryo-EM reveals mechanisms of angiotensin I-converting enzyme allostery and dimerization

Lizelle Lubbe [1, *], B Trevor Sewell [1], Jeremy D Woodward [1] and Edward D Sturrock [1, **]

[1] Department of Integrative Biomedical Sciences, Institute of Infectious Disease and Molecular Medicine, University of Cape Town, Observatory, Cape Town 7925, South Africa

* Corresponding author. Tel: +27 21 406 6312; E-mail: lizelle.lubbe@uct.ac.za

** Corresponding author. Tel: +27 21 406 6312; E-mail: edward.sturrock@uct.ac.za

Appendix table of contents

Appendix Fig S1. Page 2
SEC-HPLC trace of commercial standards used for calibration of sACE molecular weights.

Appendix Fig S2. Page 3
FSC curves and orientational distribution heatmaps of all final cryo-EM reconstructions in cryoSPARC.

Appendix Fig S3. Page 4
Map-to-model FSC curves determined by Phenix validation against the respective globally-sharpened maps.

Appendix Fig S4. Page 5
Active site breathing dynamics of the interacting N-domains in dimeric sACE^{S1211}.

Appendix Table S1. Page 6
Structures of *N*-glycans modelled in this study.

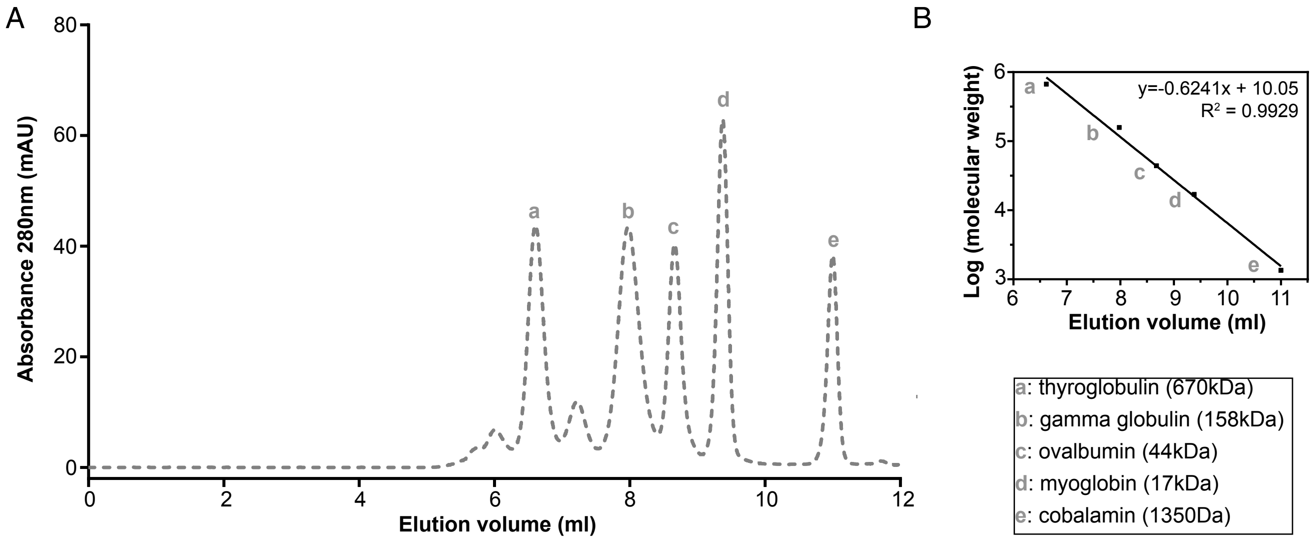

Appendix Figure S1. SEC-HPLC calibration.
A. Trace of a mixture of commercial gel filtration standards with a clearly resolved peak for each protein
B. The molecular weight calibration curve.

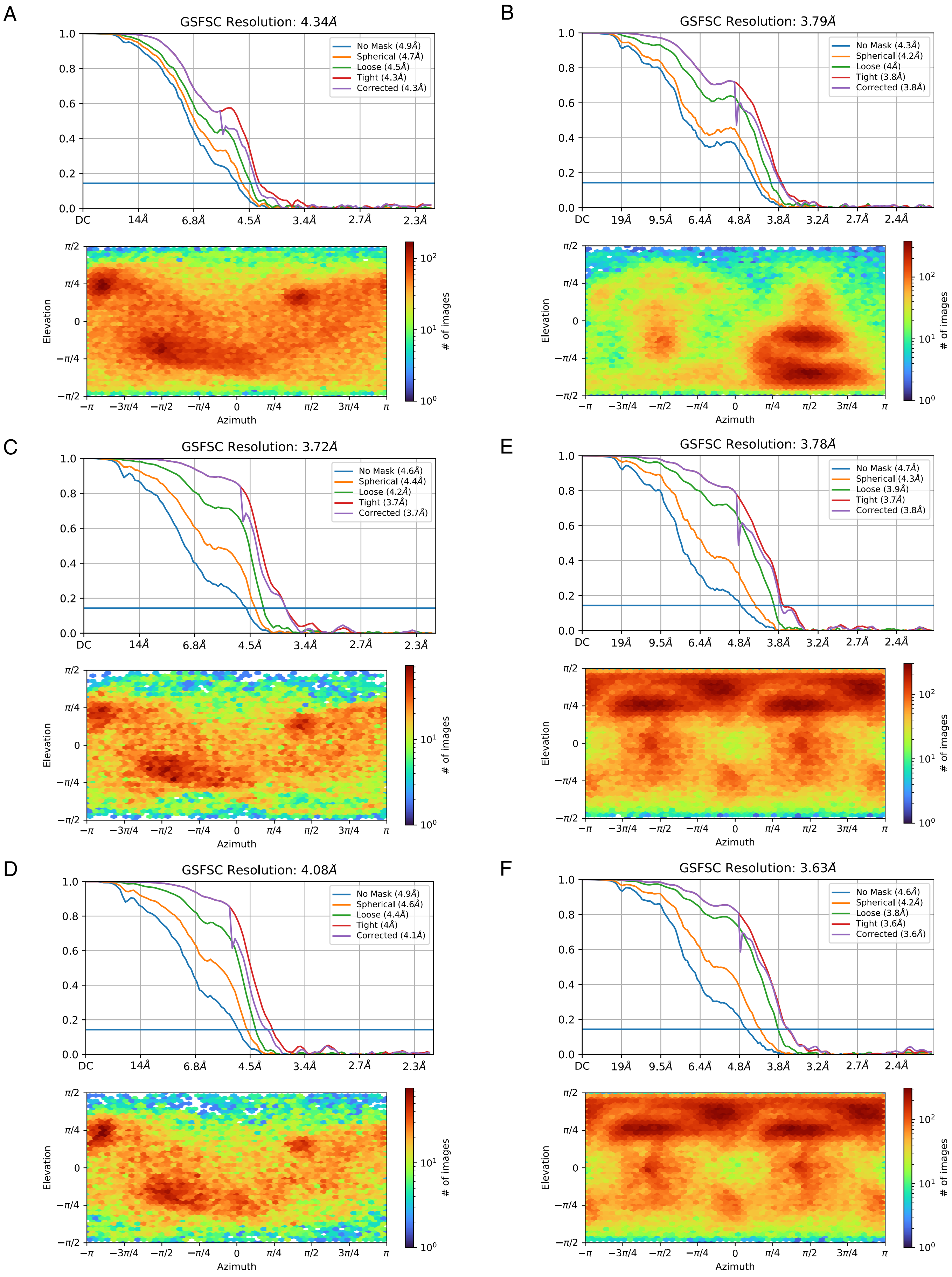

Appendix Figure S2. FSC curves and orientational distribution heatmaps of all final cryo-EM reconstructions in cryoSPARC.
A. Monomer consensus reconstruction (non-uniform refinement).
B. Dimer consensus reconstruction (non-uniform refinement).
C. Monomer N-domain reconstruction (local refinement).
D. Monomer C-domain reconstruction (local refinement).
E. Dimer two N-domain reconstruction after symmetry expansion (local refinement).
F. Dimer single N-domain after symmetry expansion (local refinement).

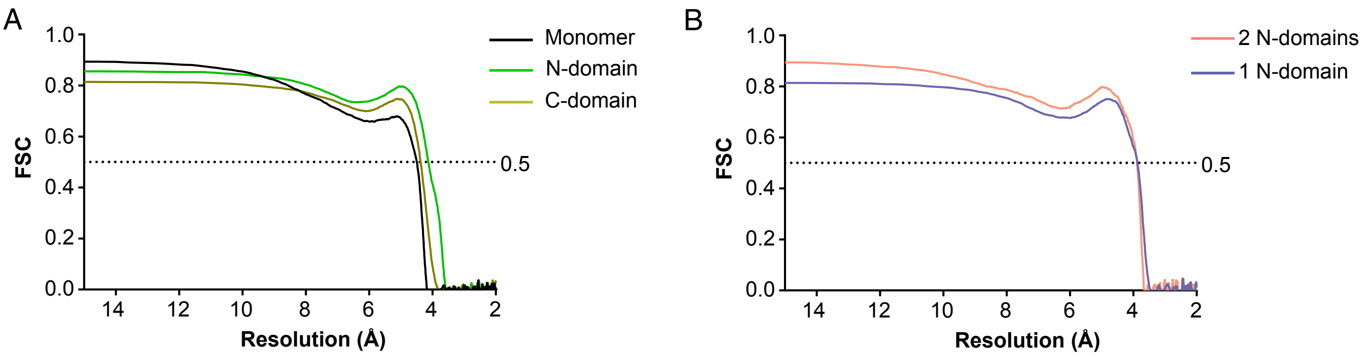

Appendix Figure S3. Map-to-model FSC curves determined by Phenix validation against the respective globally-sharpened maps.
A. Full-length and truncated single-domain monomer models.
B. Two N-domain and single N-domain dimer models.

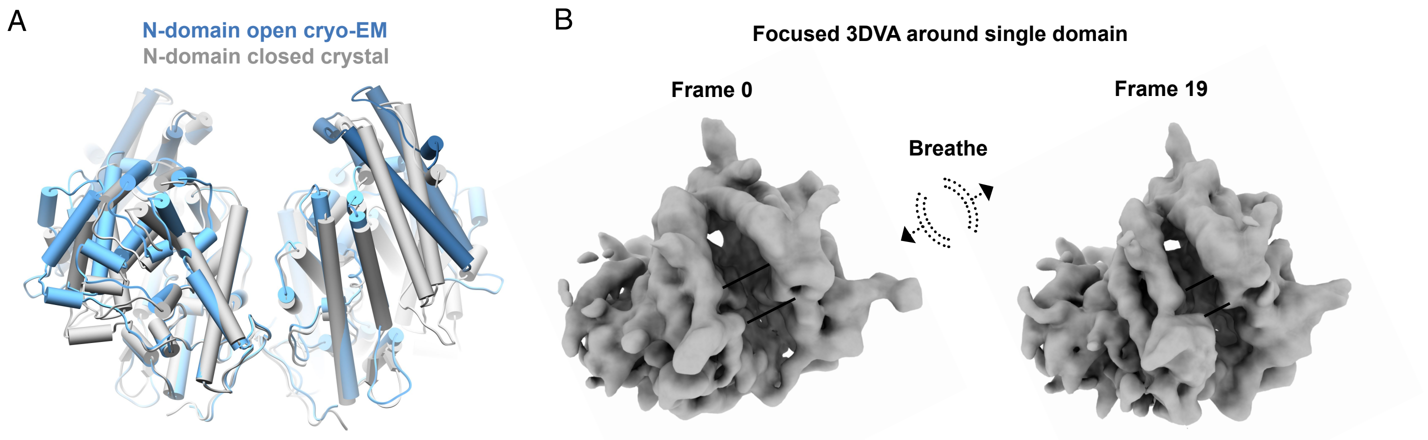

Appendix Figure S4. Active site breathing dynamics of the interacting N-domains in dimeric sACE^{S1211}.
A. Comparison between the cryo-EM structure of dimeric sACE^{S1211} in an open conformation (blue) and the truncated N-domain crystal structure in a closed conformation complexed to inhibitor (PDB ID: 4BXK; grey) where the same interface is seen between two molecules of the asymmetric unit.
B. 3DVA (component 1) performed on the dimer cryo-EM particles after local refinement of a single N-domain showed breathing and active site closure in the apo dimeric state, similar to the crystal structure conformation.

Appendix Table S1. Structures of *N*-glycans modelled in this study.

Model validation was performed against the unsharpened local refinement maps using Privateer and revealed no issues with the anomer, chirality, puckering, or conformation of the refined glycans.

	Site	Glycan structure	Glycan fitting (RSCC [a])	GlyTouCan ID [b]
Monomer N-domain	**9**	Man-b4-GlcNAc-b4-GlcNAc-b-ASN9	Man (0.64), GlcNAc (0.56), GlcNAc (0.42)	G15407YE
	**25**	Man-a6-(Man-a3-)Man-b4-GlcNAc-b4-GlcNAc-b-ASN25	Man (0.84), Man (0.88), Man (0.84), GlcNAc (0.88), GlcNAc (0.69)	G22768VO
	**45**	Man-b4-GlcNAc-b4-GlcNAc-b-ASN45	Man (0.72), GlcNAc (0.84), GlcNAc (0.70)	G15407YE
	**82**	GlcNAc-b-ASN82	GlcNAc (0.47)	G49108TO
	**117**	Man-b4-GlcNAc-b4-GlcNAc-b-ASN117	Man (0.77), GlcNAc (0.81), GlcNAc (0.69)	G15407YE
	**289**	FucMan-a6-(Man-a3-)Man-b4-GlcNAc-b4-GlcNAc-b-ASN289	Fuc (0.62), Man (0.71), Man (0.86), Man (0.76), GlcNAc (0.84), GlcNAc (0.74)	G82348BZ
	**416**	GlcNAc-b-ASN416	GlcNAc (0.55)	G49108TO
	**480**	Man-b4-GlcNAc-b4-GlcNAc-b-ASN480	Man (0.59), GlcNAc (0.76), GlcNAc (0.77)	G15407YE
Monomer C-domain	**648**	Man-a6-Man-b4-GlcNAc-b4-GlcNAc-b-ASN648	Man (0.73), Man (0.66), GlcNAc (0.64), GlcNAc (0.61)	G22573RC
	**666**	GlcNAc-b-ASN666	GlcNAc (0.41)	G49108TO
	**685**	GlcNAc-b4-GlcNAc-b-ASN685	GlcNAc (0.76), GlcNAc (0.74)	G42666HT
	**731**	GlcNAc-b4-GlcNAc-b-ASN731	GlcNAc (0.62), GlcNAc (0.41)	G42666HT
	**913**	GlcNAc-b-ASN913	GlcNAc (0.59)	G49108TO
Dimer single N-domain	**9**	Man-b4-GlcNAc-b4-GlcNAc-b-ASN9	Man (0.71), GlcNAc (0.70), GlcNAc (0.56)	G15407YE
	**25**	GlcNAc-b2-Man-a6-Man-b4-GlcNAc-b4-GlcNAc-b-ASN25	GlcNAc (0.55), Man (0.72), Man (0.86), GlcNAc (0.77), GlcNAc (0.57)	G12045WP
	**45**	Man-a2-Man-b3-GlcNAc-b4-GlcNAc-b-ASN45	Man (0.75), Man (0.75), GlcNAc (0.69), GlcNAc (0.48)	-
	**82**	GlcNAc-b4-GlcNAc-b-ASN82	GlcNAc (0.75), GlcNAc (0.23)	G42666HT
	**117**	Man-b4-GlcNAc-b4-GlcNAc-b-ASN117	Man (0.82), GlcNAc (0.78), GlcNAc (0.53)	G15407YE
	**289**	FucGlcNAc-b2-Man-a6-(Man-a3-)Man-b4-GlcNAc-b4-GlcNAc-b-ASN289	Fuc (0.65), GlcNAc (0.75), Man (0.80), Man (0.78), Man (0.72), GlcNAc (0.81), GlcNAc (0.74)	G45889JQ
	**416**	GlcNAc-b-ASN416	GlcNAc (0.43)	G49108TO
	**480**	Man-b4-GlcNAc-b4-GlcNAc-b-ASN480	Man (0.74), GlcNAc (0.78), GlcNAc (0.69)	G15407YE

[a] RSCC, real space correlation coefficient. Values are given for each sugar in brackets in the order of the glycan structure; [b] GlyTouCan repository accessible at https://glytoucan.org
