# Supplementary material for: Cryo‐EM reveals mechanisms of angiotensin I‐converting enzyme allostery and dimerization
Source: EMBO J. 2022 Jul 12;41(16):e110550. doi: 10.15252/embj.2021110550 (PMC9379546; doi:10.15252/embj.2021110550)
Supplement: Supplementary file 2 — Expanded View Figures PDF [file EMBJ-41-e110550-s008.pdf]

Expanded View Figures

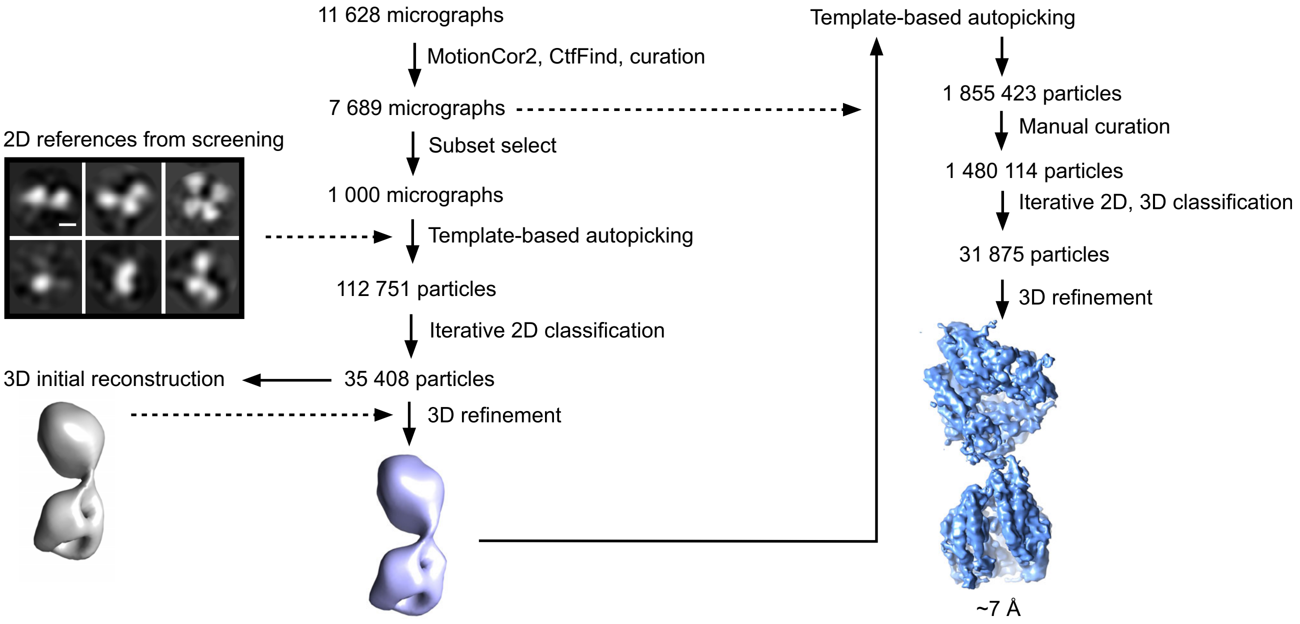

Figure EV1. Schematic of initial image processing from all micrographs in RELION using 2D references obtained during sample screening.

A 3D reconstruction of the dimer was not obtained while the monomer yielded a final ~7 Å reconstruction. Scale bar: 50 Å.

 © 2022 The Authors.

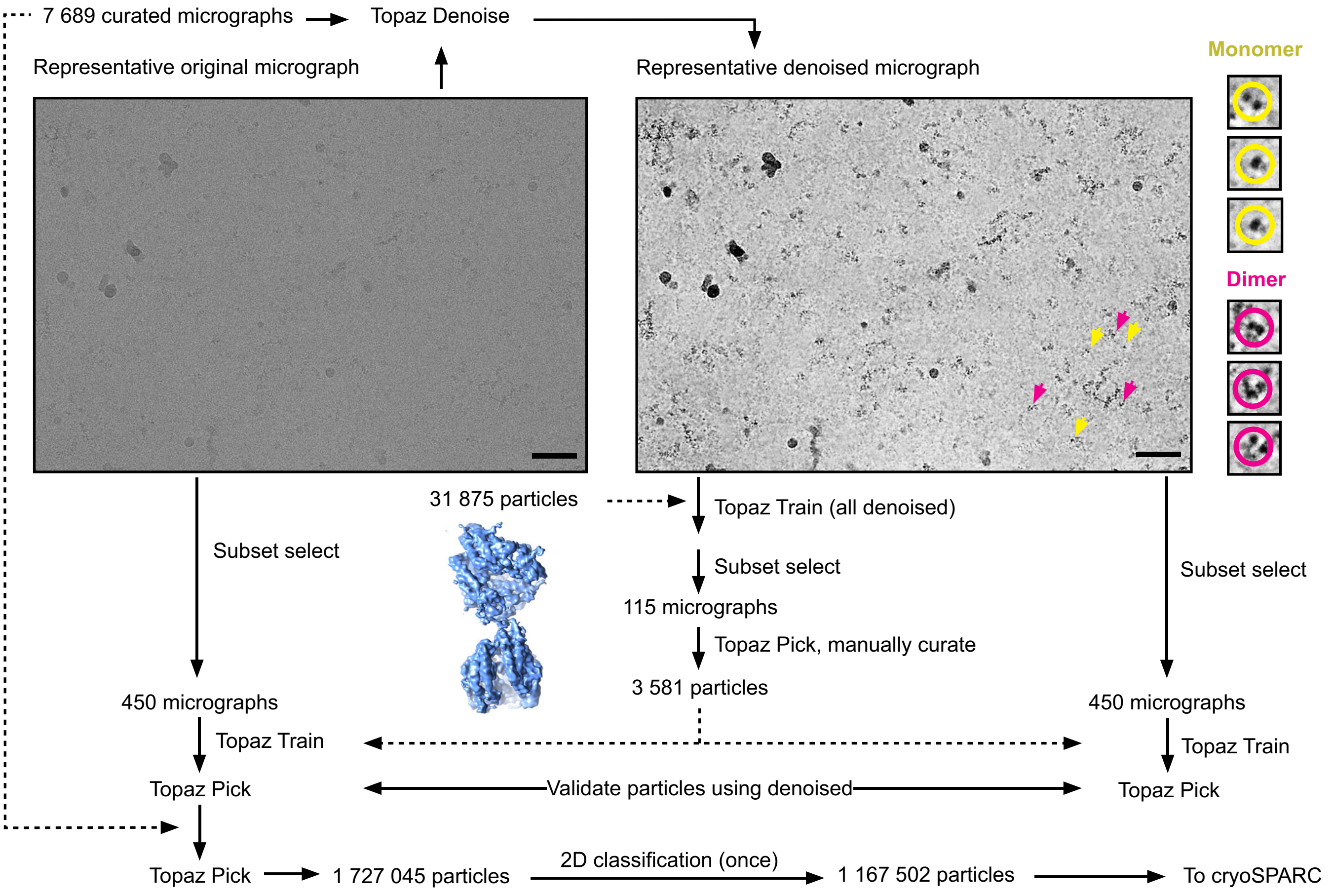

Figure EV2. Schematic depiction of Topaz processing for sACE^{S1211}.

Micrographs were denoised and training of a picking model for sACE^{S1211} done in parallel on noisy micrographs and their denoised counterparts. The 31,875 monomer particles, which refined to ~7 Å resolution during initial processing, were used as positive labels. Coordinates obtained from the denoised micrographs were used for manual validation and optimization of picking parameters. After validating picking from noisy micrographs by comparison with the corresponding denoised coordinates, the model trained on noisy data was used for picking from all micrographs. Solid arrows indicate sequential jobs where the results from one step fed directly into the next. Dashed arrows indicate where results from earlier steps were used as additional input. Scale bar: 50 nm.

© 2022 The Authors.

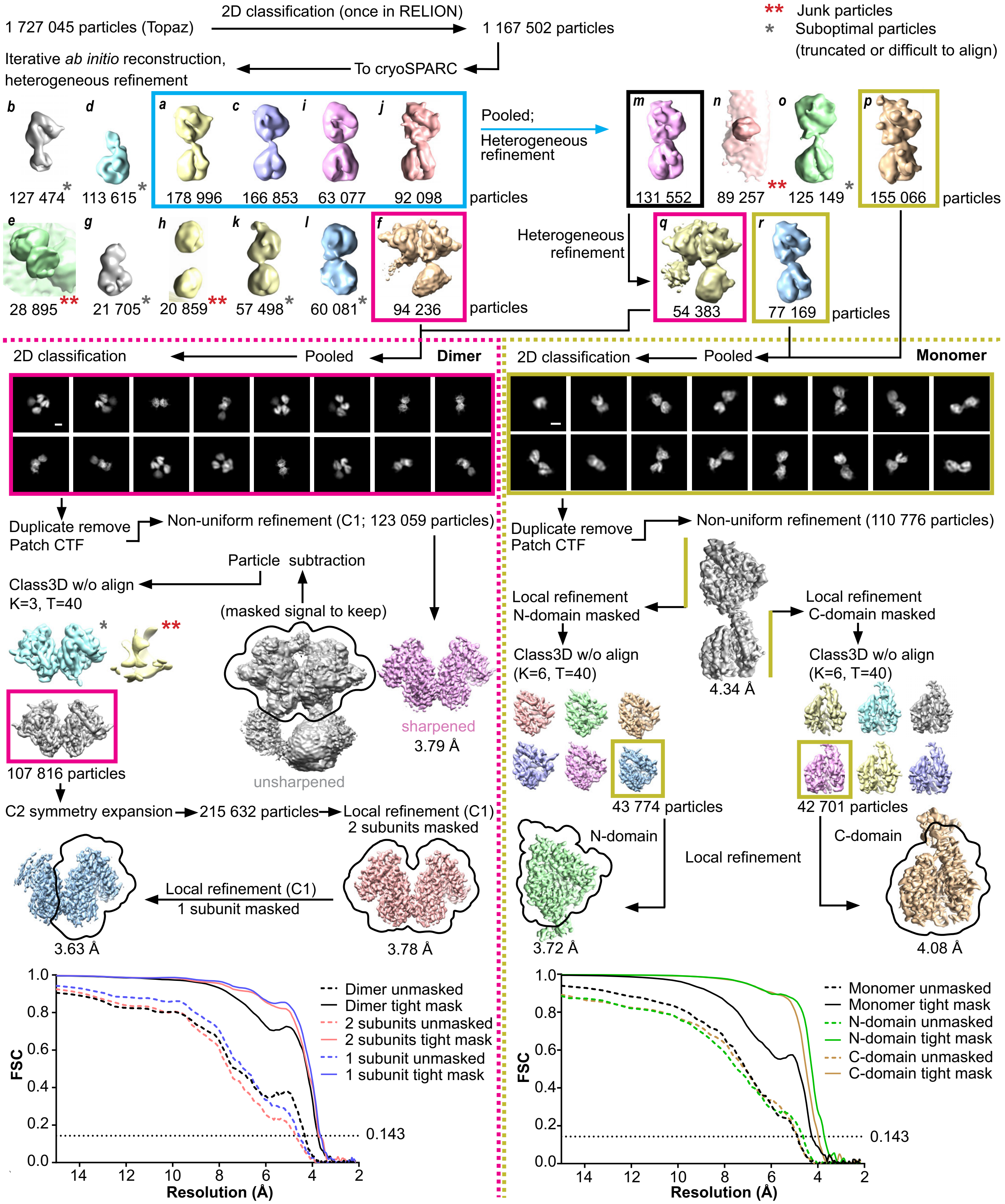

Figure EV3. Schematic depiction of image processing performed to obtain monomeric and dimeric sACE[S1211] reconstructions.

Class3D without align and particle subtraction were done in RELION with all other stages performed in cryoSPARC. 3D classes are labeled *a-r*. Scale bar: 50 Å.

© 2022 The Authors.

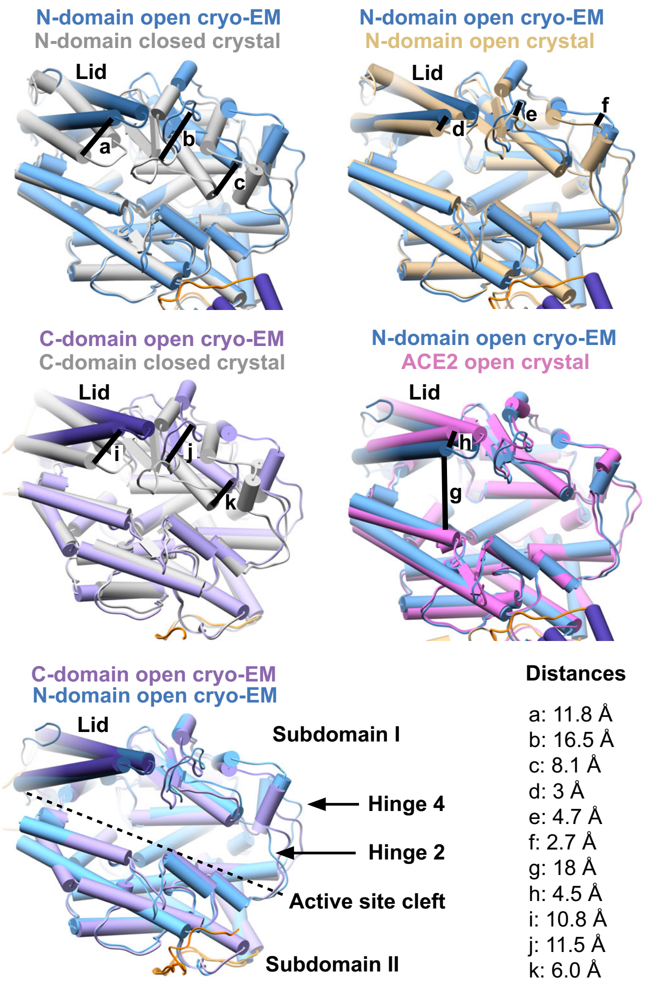

Figure EV4. Comparison between the monomeric sACE^{S1211} structure determined here by cryo-EM and published crystal structures.

Differences in active site cleft exposure are shown for the truncated single-domain crystal structures (C-domain PDB ID: 1O8A; N-domain PDB ID: 4BXK for the closed conformation and PDB ID: 6ZPQ for the open conformation) and the open conformation of ACE2 (PDB ID: 1R42). Hinge regions 2 and 4, which are key for active site closure, are indicated.

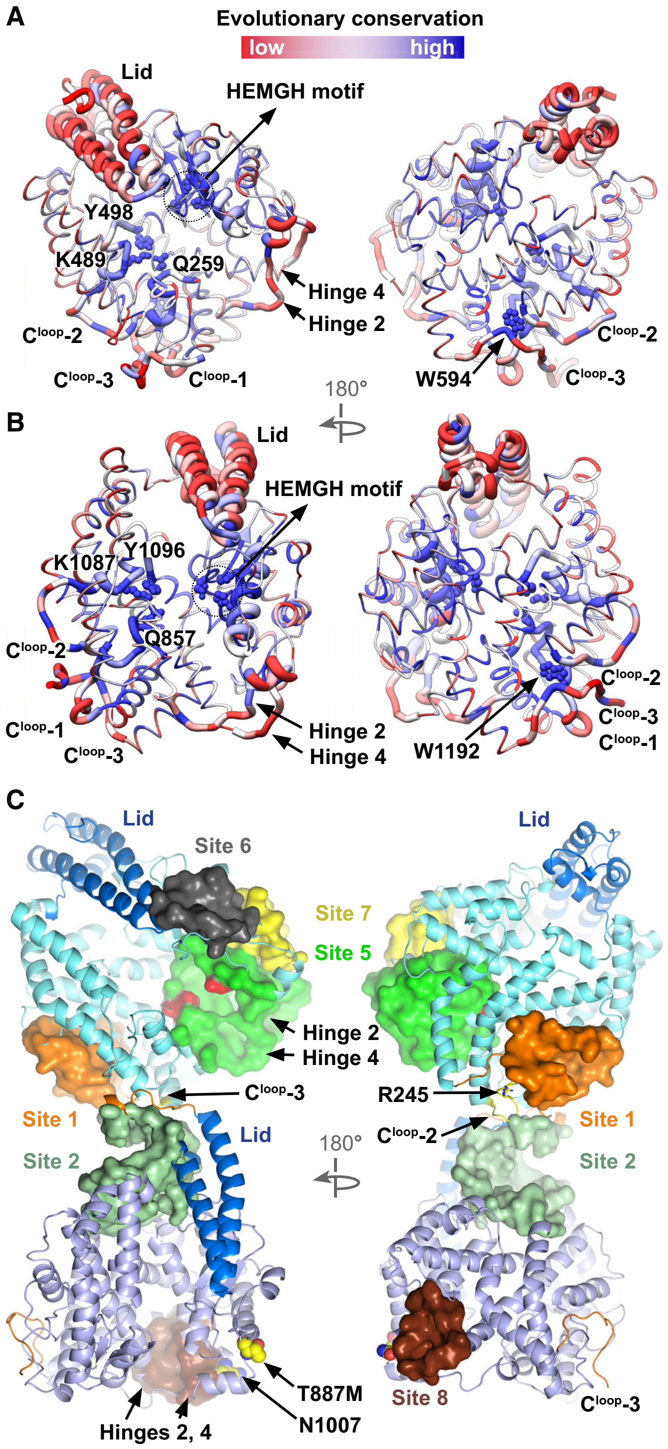

Figure EV5. Evolutionary conservation and allosteric regulation of sACE^{S1211}.

Evolutionary conservation was calculated using ConSurf and mapped onto the protein structure with high and low conservation shown in blue and red, respectively. The lid region and hinges 2 and 4 were poorly conserved while the zinc-binding motif, catalytic anchor (Q259/857, K489/1,087, and Y498/1,096), and a key tryptophan residue (W594/1,192) on C^{loop}-3 were highly conserved in both domains. The zinc-binding motif and catalytic anchor residues are shown as spheres while C^{loop}-2, C^{loop}-3, the two hinges, and the lid region are shown as thick helices/coils. Allosteric sites were predicted for the N- and C-domain orthosteric sites (zinc-binding and catalytic anchor residues) using CorrSite2.0.

A, B N- and C-domain structures, respectively, with evolutionary conservation mapped.

C Allosteric sites (Sites 1–8) predicted for sACE^{S1211} shown as colored surfaces. The central active site clefts (Sites 3 and 4) allosterically regulated each other but were omitted from the figure for clarity. Sites 1, 2, and 6 were predicted to only regulate the N-domain orthosteric site, while Sites 5 and 7 also affected the C-domain orthosteric site. Site 8 was predicted to regulate the C- but not the N-domain orthosteric site. The Alzheimer's disease-associated mutation T887M is shown as a yellow sphere. The unique D354 and E431 residues in the distal N-domain prime subsite extension are shown as red spheres for orientation.

© 2022 The Authors.
