# Supplementary material for: Cryo‐EM reveals mechanisms of angiotensin I‐converting enzyme allostery and dimerization
Source: EMBO J. 2022 Jul 12;41(16):e110550. doi: 10.15252/embj.2021110550 (PMC9379546; doi:10.15252/embj.2021110550)
Supplement: Supplementary file 3 — Table EV1 [file EMBJ-41-e110550-s002.docx]

**Table EV1. PI-score analysis of protein-protein interactions** ^a^.

PDB ID 7Q4D is the cryo-EM structure reported in the present study. The first 23 crystal structures showed the same dimerization interface as the cryo-EM structure between two molecules in the asymmetric unit while PDB ID 2C6F and 2C6N showed a different dimerization interface.

| **PDB ID** | **Chains** | **PI-score** | **^i^N_res_** | **Polar** | **Hydrophobic** | **Charged** | **Conservation** | **^i^N_pairs_** | **sc** | **N_HB_** | **N_SB_** | **Δ^i^G (kcal/mol)** | **^i^Area (Å^2^)** | **Δ^i^G**  **P- value** |
| --- | --- | --- | --- | --- | --- | --- | --- | --- | --- | --- | --- | --- | --- | --- |
| **4UFB** | D_B | 2.42 | 27 | 0.519 | 0.074 | 0.296 | 0.185 | 30 | 0.78 | 8 | 2 | -7.76 | 976.62 | 0.26 |
| **5AMB** | B_A | 2.38 | 27 | 0.519 | 0.074 | 0.296 | 0.185 | 30 | 0.77 | 8 | 2 | -7.32 | 969.91 | 0.28 |
| **6TT4** | B_A | 2.32 | 22 | 0.545 | 0.091 | 0.273 | 0.182 | 26 | 0.753 | 8 | 0 | -6.36 | 944.66 | 0.34 |
| **6EN6** | B_A | 2.38 | 26 | 0.538 | 0.077 | 0.269 | 0.192 | 29 | 0.758 | 8 | 5 | -5.54 | 1029.63 | 0.36 |
| **6EN5** | B_A | 2.49 | 26 | 0.538 | 0.077 | 0.269 | 0.192 | 29 | 0.783 | 8 | 3 | -6.85 | 959.87 | 0.26 |
| **3NXQ** | B_A | 2.45 | 26 | 0.538 | 0.077 | 0.269 | 0.192 | 29 | 0.770 | 10 | 1 | -8.87 | 952.13 | 0.24 |
| **6F9V** | B_A | 2.5 | 26 | 0.538 | 0.077 | 0.269 | 0.192 | 29 | 0.781­ | 8 | 3 | -7.43 | 1009.36 | 0.26 |
| **5AMC** | B_A | 2.52 | 28 | 0.5 | 0.071 | 0.286 | 0.214 | 31 | 0.769 | 8 | 2 | -6.2 | 972.02 | 0.34 |
| **6QS1** | B_A | 2.34 | 27 | 0.519 | 0.074 | 0.296 | 0.185 | 30 | 0.770 | 8 | 4 | -8.13 | 984.36 | 0.22 |
| **5AM8** | D_B | 2.31 | 27 | 0.519 | 0.074 | 0.296 | 0.185 | 30 | 0.763 | 8 | 0 | -6.85 | 957.87 | 0.28 |
| **6F9R** | B_A | 2.57 | 28 | 0.5 | 0.071 | 0.286 | 0.214 | 31 | 0.784 | 8 | 3 | -8.23 | 958.78 | 0.22 |
| **5AMA** | D_B | 2.43 | 28 | 0.5 | 0.071 | 0.286 | 0.214 | 31 | 0.757 | 8 | 1 | -6.6 | 953.35 | 0.3 |
| **4CA6** | B_A | 2.33 | 27 | 0.519 | 0.074 | 0.296 | 0.185 | 30 | 0.768 | 8 | 0 | -8.45 | 957.79 | 0.21 |
| **6ZPT** | D_C | 1.62 | 32 | 0.438 | 0.063 | 0.375 | 0.25 | 33 | 0.656 | 13 | 4 | -3.85 | 1191.51 | 0.55 |
| **6H5X** | B_A | 2.53 | 28 | 0.5 | 0.071 | 0.286 | 0.214 | 31 | 0.781 | 8 | 6 | -7.37 | 991.65 | 0.26 |
| **6ZPQ** | D_B | 2.16 | 24 | 0.583 | 0.083 | 0.25 | 0.167 | 27 | 0.735 | 8 | 1 | -5.5 | 1035.7 | 0.41 |
| **2XYD** | B_A | 2.42 | 28 | 0.5 | 0.071 | 0.286 | 0.214 | 31 | 0.761 | 8 | 0 | -9.65 | 926.81 | 0.17 |
| **5AM9** | D_B | 2.29 | 27 | 0.519 | 0.074 | 0.296 | 0.185 | 30 | 0.763 | 8 | 0 | -8.66 | 931.43 | 0.2 |
| **4BXK** | B_A | 2.2 | 27 | 0.519 | 0.074 | 0.296 | 0.185 | 30 | 0.744 | 8 | 1 | -6.98 | 958.98 | 0.3 |
| **4UFA** | B_A | 2.42 | 27 | 0.519 | 0.074 | 0.296 | 0.185 | 30 | 0.779 | 8 | 1 | -8.12 | 995.66 | 0.25 |
| **6TT1** | B_A | 2.49 | 28 | 0.5 | 0.071 | 0.286 | 0.214 | 31 | 0.765 | 8 | 2 | -6.83 | 966.74 | 0.33 |
| **6TT3** | B_A | 2.24 | 25 | 0.56 | 0.08 | 0.28 | 0.16 | 28 | 0.774 | 8 | 4 | -8.12 | 979.9 | 0.23 |
| **4BZS** | B_A | 2.38 | 25 | 0.56 | 0.08 | 0.24 | 0.2 | 28 | 0.761 | 8 | 2 | -9.53 | 953.88 | 0.14 |
| **2C6F** | B_A | 0.92 | 34 | 0.412 | 0.235 | 0.176 | 0.235 | 33 | 0.615 | 5 | 2 | -2.51 | 927.92 | 0.63 |
| **2C6N** | B_A | 0.95 | 32 | 0.438 | 0.25 | 0.188 | 0.188 | 31 | 0.616 | 6 | 2 | -2.26 | 809.28 | 0.74 |
| **7Q4D** | B_A | -0.04 | 24 | 0.583 | 0.083 | 0.25 | 0.167 | 27 | 0.497 | 4 | 1 | -7.29 | 1054.76 | 0.27 |

^a^ Abbreviations: ^i^N_res_, number of interface residues; ^i^N_pairs_, number of interface pairs; sc, shape complementarity; N_HB_, number of hydrogen bonds; N_SB_, number of salt bridges; Δ^i^G, interface solvation energy gain;  ^i^Area (Å^2^), interface area.
