# Supplementary material for: Cryo‐EM reveals mechanisms of angiotensin I‐converting enzyme allostery and dimerization
Source: EMBO J. 2022 Jul 12;41(16):e110550. doi: 10.15252/embj.2021110550 (PMC9379546; doi:10.15252/embj.2021110550)
Supplement: Supplementary file 4 — Movie EV1 [file EMBJ-41-e110550-s001.zip › EMBOJ-2021-110550R_MovieEV1/EMBOJ-2021-110550R_Movie Legend for Movie EV1.docx]

**Extended View Movie Legend for Movie EV1** (related to Figure 6).

Bending (component 0) and breathing (component 1 of the full-length soluble monomer and component 0 of each focused domain) motions observed for monomeric sACE^S1211^ by 3D variability analysis.
