# Supplementary material for: Cryo‐EM reveals mechanisms of angiotensin I‐converting enzyme allostery and dimerization
Source: EMBO J. 2022 Jul 12;41(16):e110550. doi: 10.15252/embj.2021110550 (PMC9379546; doi:10.15252/embj.2021110550)
Supplement: Supplementary file 5 — Movie EV2 [file EMBJ-41-e110550-s003.zip › EMBOJ-2021-110550R_MovieEV2/EMBOJ-2021-110550R_Movie Legend for Movie EV2.docx]

**Extended View Movie Legend for Movie EV2** (related to Figure 6).

Pivoting (mode 7), bending (modes 8 and 9), and contraction (mode 10) of the interdomain linker (N-domain C^loop^-3) observed for full-length soluble monomeric sACE^S1211^ by normal mode analysis.
