# Supplementary material for: Cryo‐EM reveals mechanisms of angiotensin I‐converting enzyme allostery and dimerization
Source: EMBO J. 2022 Jul 12;41(16):e110550. doi: 10.15252/embj.2021110550 (PMC9379546; doi:10.15252/embj.2021110550)
Supplement: Supplementary file 6 — Movie EV3 [file EMBJ-41-e110550-s006.zip › EMBOJ-2021-110550R_MovieEV3/EMBOJ-2021-110550R_Movie Legend for Movie EV3.docx]

**Extended View Movie Legend for Movie EV3** (related to Figure 6).

Sliding (mode 7) and clam shell-like (mode 8) opening and closing of the active site cleft between subdomains I and II observed for the focused N- and C-domain models of monomeric sACE^S1211^ by normal mode analysis.
