# Supplementary material for: Cryo‐EM reveals mechanisms of angiotensin I‐converting enzyme allostery and dimerization
Source: EMBO J. 2022 Jul 12;41(16):e110550. doi: 10.15252/embj.2021110550 (PMC9379546; doi:10.15252/embj.2021110550)
Supplement: Supplementary file 7 — Movie EV4 [file EMBJ-41-e110550-s007.zip › EMBOJ-2021-110550R_MovieEV4/EMBOJ-2021-110550R_Movie Legend for Movie EV4.docx]

**Extended View Movie Legend for Movie EV4** (related to Figures 6 and 9).

Swinging (components 0 and 4), twisting (component 1), and contraction (component 2) of the interdomain linker (N-domain C^loop^-3) with large-scale C-domain motions observed for dimeric sACE^S1211^ by 3D variability analysis.
