# Supplementary material for: Cryo‐EM reveals mechanisms of angiotensin I‐converting enzyme allostery and dimerization
Source: EMBO J. 2022 Jul 12;41(16):e110550. doi: 10.15252/embj.2021110550 (PMC9379546; doi:10.15252/embj.2021110550)
Supplement: Supplementary file 8 — Movie EV5 [file EMBJ-41-e110550-s004.zip › EMBOJ-2021-110550R_MovieEV5/EMBOJ-2021-110550R_Movie Legend for Movie EV5.docx]

**Extended View Movie Legend for Movie EV5** (related to Figure 9).

Swinging (mode 7), rotation (mode 8), and large-scale shift (mode 9) of the C-domains observed for full-length soluble dimeric sACE^S1211^ by normal mode analysis.
