# Supplementary figures and images for: Cryo‐EM reveals mechanisms of angiotensin I‐converting enzyme allostery and dimerization
Source: EMBO J. 2022 Jul 12;41(16):e110550. doi: 10.15252/embj.2021110550 (PMC9379546; doi:10.15252/embj.2021110550)

Source data for Fig 2A.

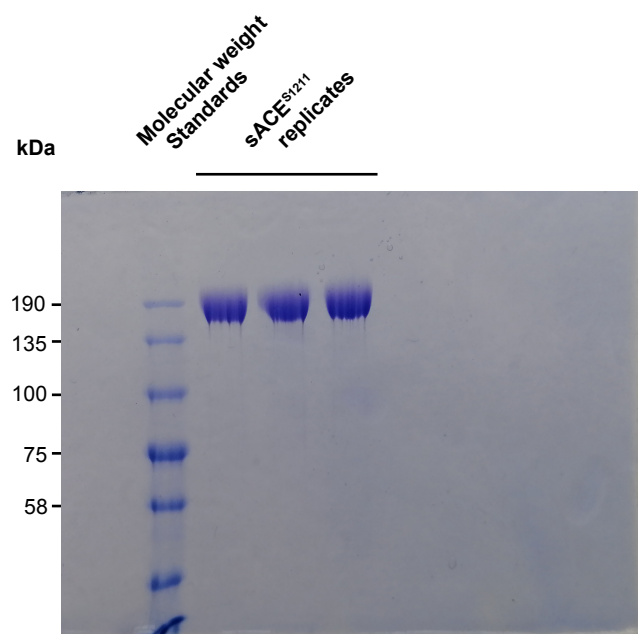

Source data for Fig 2C.

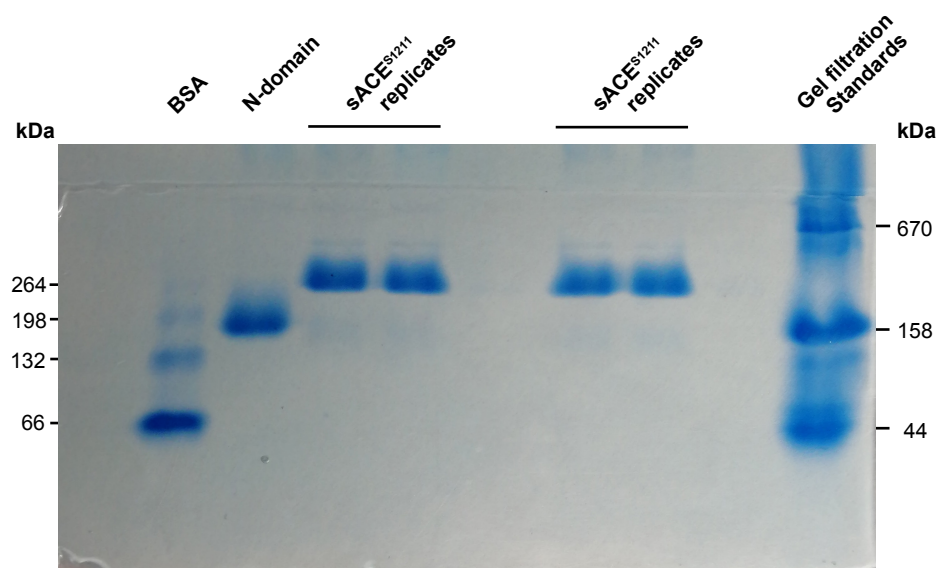

Supplement: Supplementary file 9 — Source Data for Figure 2 [file EMBJ-41-e110550-s010.pdf]
