# Supplementary material for: Cryo‐EM reveals mechanisms of angiotensin I‐converting enzyme allostery and dimerization
Source: EMBO J. 2022 Jul 12;41(16):e110550. doi: 10.15252/embj.2021110550 (PMC9379546; doi:10.15252/embj.2021110550)
Supplement: Supplementary file 10 [file EMBJ-41-e110550-s009.pdf]

THE EMBO JOURNAL

Cryo-EM reveals mechanisms of angiotensin I-converting enzyme allostery and dimerization

Lizelle Lubbe, Bryan Trevor Sewell, Jeremy Woodward, and Edward Sturrock
DOI: 10.15252/embj.2021110550

Corresponding author(s): Lizelle Lubbe (lbbliz002@myuct.ac.za) , Edward Sturrock (Edward.Sturrock@uct.ac.za)

Review Timeline:

Submission Date:	29th Dec 21
Editorial Decision:	24th Feb 22
Revision Received:	21st Apr 22
Accepted:	27th May 22

Editor: Kelly Anderson/Karin Dumstrei

Transaction Report:

(Note: With the exception of the correction of typographical or spelling errors that could be a source of ambiguity, letters and reports are not edited. Depending on transfer agreements, referee reports obtained elsewhere may or may not be included in this compilation. Referee reports are anonymous unless the Referee chooses to sign their reports.)

1st Editorial Decision 24th Feb 2022

Dear Lizelle,

Thank you for submitting your manuscript to The EMBO Journal. The handling editor for your manuscript is Dr. Kelly Anderson, but as Kelly is away this week, I am stepping in as secondary editor on the manuscript.

I am sorry for the delay in getting back to you with a decision, but we have now received the two referee reports on your study. We are still waiting for the input from a third referee, but at this stage I don't think we will receive it. I will therefore go ahead with the two referee reports on hand.

As you can see from the comments, the referees find the analysis interesting and suitable for publication here. They raise several different concerns that should be addressed. Both referees note that the writing of the manuscript has to be improved and made more accessible.

It would be good if you could discuss the revisions further with Kelly when she is back either via email or a video call.

When preparing your letter of response to the referees' comments, please bear in mind that this will form part of the Review Process File, and will therefore be available online to the community. For more details on our Transparent Editorial Process, please visit our website: https://www.embopress.org/page/journal/14602075/authorguide#transparentprocess

We generally allow three months as standard revision time. As a matter of policy, competing manuscripts published during this period will not negatively impact on our assessment of the conceptual advance presented by your study. However, we request that you contact the editor as soon as possible upon publication of any related work, to discuss how to proceed. Should you foresee a problem in meeting this three-month deadline, please let us know in advance and we may be able to grant an extension.

Thank you for the opportunity to consider your work for publication.

Yours sincerely,

Karin

Karin Dumstrei, PhD
Senior Editor
The EMBO Journal

Instructions for preparing your revised manuscript:

I have attached a guide with helpful tips on how to prepare the revised version.

Guide For Authors: https://www.embopress.org/page/journal/14602075/authorguide

We realize that it is difficult to revise to a specific deadline. In the interest of protecting the conceptual advance provided by the work, we recommend a revision within 3 months (25th May 2022). Please discuss the revision progress ahead of this time with the editor if you require more time to complete the revisions. Use the link below to submit your revision:

https://emboj.msubmit.net/cgi-bin/main.plex

Referee #2:

The manuscript of Lubbe et al. is the summation of a huge amount of outstanding work. The authors are to be congratulated. There is no question that the observations in this manuscript are remarkable and outstanding and deserve to be published in EMBO. That said, the presentation of the data in the manuscript is not optimal. First, I am sure that Lubbe et al. must realize that virtually all readers of EMBO (and essentially all readers on-line) are not expert in structural analysis and I have my doubts as to exactly how many readers will remain engaged to the end of this long manuscript (my left hand has 5 fingers). Thus, I believe it absolutely incumbent upon the authors to shorten and simplify their presentation of the data. In fact, it is to the authors best interest to make this wonderful study intellectually accessible to more readers. Several suggests are below:

Introduction

1. The introduction is way too long. The introduction of ACE in the introduction can be summarized in many fewer words.

2. Despite the long introduction, the authors do not give any real introduction to cryo electron microscopy. What is it exactly in simple terms? How can it answer questions in a complementary fashion to crystallography.

3. It would be very helpful to the reader to summarize clearly (as if you were explaining to a 1st year graduate student) what are the 3 or 4 most important questions you will investigate and answer in your study.

Results

1. Also too long. In some ways, this reads like a Ph.D. thesis. I would strongly urge the authors to focus on what they view as the most important questions and explain why they are the most important questions.

2. I would recommend the authors use several more subheaders ('cryo electron microscopy' is the first subheader as example) but use the subheaders to make simple declarative statements and summarize what they find in that part of the results (just as an example: 'assembly of x images shows ....' or something like 'both domains of ACE are closely associated'). The authors can do it better than I can but use these liberally to take the hand of the reader and explain the key conclusions of the data.

3. There are parts of the second half of the results where it is not clear what exactly is fact (based on the data) and what is speculation that may or may not be true. The section after 'Voronov et al. found...' is like that. Another example is:

"The proximity of the N480 glycan could likely contribute to a change in the Cloop-1 dynamics and loss of the E596-R245 interaction could further destabilize Cloop-3. Disorder of the dimer C-domains may also cause a loss of R240 and Y241 hydrogen bonds to the C domain lid N681, which would contribute to destabilization of Cloop-1. C-domain disorder could also destabilize the Y607-E161-R479 interactions."

While all this may eventually be found to be true, in the context of this long study - and to make things easier for the reader - I would pare much of this speculation (could likely, may also, could also) from the Results and keep the Results simpler, focusing on what you find to be true. Even better would be to pose relatively simple (ie graduate student level) specific questions, then give data, then summarize conclusions.

4. As a reader, I would urge a slightly greater focus on insight into catalytic activity and whether these data lead to general conclusions about enzymes similar to ACE. Concerning one section:

"Although the density is not of sufficient resolution to identify the potential ligand, the open N-domain active site is clearly primed for substrate binding to the zinc site and these 3 catalytic anchor residues. Although the domains are closely associated, full-length sACES1211 does not have a continuous active site channel for substrate cleavage. The N-domain active site cleft is instead inclined by ~30 degrees relative to the C-domain. This means that substrate molecules are unlikely to get cleaved by the one domain and then passed to the next. Endopeptidase activity at multiple sites has been observed for sACE with large substrates such as amyloid-β Larmuth et al., 2016; Oba et al., 2005; Zou et al., 2009). However, it is clear from this domain orientation that such substrates are unlikely to bind simultaneously to both domains to yield this activity. Yet, despite this independent active site arrangement, cooperativity is known to occur between the domains."

I do not understand how you conclude that substrates are unlikely to go from one site to the next site based on only a 30 degree incline. I would think that the probability of going from one site to the other site of a single enzyme is still far higher (given local concentration analysis) than the probability of going to a different site of a different ACE molecule. Also, your rationale for concluding that a single larger peptide may not bind simultaneously to both domains is also unclear (though perhaps true). Please make this section - which is very important - clearer.

Discussion

1. I thought the discussion was good.

Movies -

1. Very nice. It is probably not possible but it would be great to add a voice track explaining in a very few words what is happening.

Referee #3:

The manuscript by Lubbe et al. addresses the structural organization of angiotensin-I converting enzyme (ACE) as determined by cryo-EM, image processing and significant structure sorting and focused refinements. This provides novel insights

considering that this protein has until now only been studied at the level of the individual N- and C-terminal domains (crystal structures). The study now comprises the almost complete sequence (1211 residues out of 1277, the very C-terminus comprises a transmembrane region which hampers protein solubility). Furthermore, an important aspect is the presence of numerous protein modifications (glycans) that were not present in previous structures. The present work describes the overall architecture of the multi-domain protein, localizes glycan sites and gives insights into substrate-binding pockets (though no substrates are present). As the protein can form dimers or monomers (separated by image processing), the inter-domain protein-protein interactions can be addressed, in particular for the N-terminal domain pair that is also the best resolved region and is shown to induce dimerization. Interesting is also that an interdomain linker in fact wraps around a domain instead of serving as a distance-creating linker.
Altogether, this work appears to be an interesting and important step forward in understanding the structure and function of ACE. The cryo-EM part appears sound technically and involved a number of specific approaches including sorting and focused refinements. The resolution is fine to address the overall structure and refine the atomic model (considering that crystal structures of parts exist) but one should be more careful with regards to side-chains positions/conformations and avoid over-interpretation (e.g. hydrogen-bonding between residues whose interpretation depend on precise side-chain conformations or a water molecule in a 3.7 Å map cannot be addressed). That being said, the structure as it is represents an important finding by itself without going too far in interpretation. The part on the normal mode analysis etc. is less strong, probably also because some parts are a bit lengthy and could be described more concisely. I would suggest keeping the focus on the new structure which is the main new finding without going too much into hypothesis-driven NMA, flexibility etc. A question the reader my have from the beginning is in how far this is similar to the ACE2 receptor which is involved in SARS-CoV spike recognition; the answer to that comes at the end but could be introduced at the beginning.

Detailed points:
- clarify: N-glycosylation: was that analysed by MS? Do data exist that could be discussed in the context of the structure?
- full-length: almost (page 7)
- 7 Å reconstruction: obtained how (page 7)
- page 8: what is the meaning of the "truncated single domain forms" found in the classification?
- page 8: "attracting" this is well known and doesn't refer to the 2021 paper cited here but instead to correlation artefacts known in image processing for more than 20 years; this is reference bias due to correlation artefacts; no need for this new naming
- page 8/9 local refinement, focused 3D classifications etc. and elsewhere in the MS and methods: as the structure sorting and refinements heavily rely on focused classifications and refinements it may be worth mentioning some key references such as Curr Op Struct Biol 2017, eLife 2018, Biosci Rep 2018 etc.
- references missing for Relion, Isolde, Phenix etc.
- sphericity values: defined how?
- dimer processing: good that no C2 symmetry was imposed; yet, afterwards C2 symmetry expansion is mentioned, was it finally imposed or not or fully relaxed for final reconstructions?
- particle subtraction etc.: with re-centering? that could improve image processing even further
- "classes" should be better called class averages
- expected pattern of glycosylation: determined how?
- final resolution of 3.6 Å, more data needed to go further? Even a 3.3-3.4 Å map would make a significant difference for atomic model building, side chain positioning etc. (which looks approximate in some places as visible in the figures)
- presence of proline next to Asn: if this hinders glycosylation then such places should probably not be called "sites"; instead only those really modified and confirmed by MS. E.g. N1196 and N494 appear not to be sites
- Diffmaps, explain
- N131, N1162: partial density; could these be partially modified? Anything known from MS?
- ACE2: what is the sequence identity and similarity? "homology" is not the correct term here. Topic to be introduced earlier
- activation of a water molecule: based on which knowledge? Any paper on this?
- visualisation of a water molecule: in the 3.5 - 4.5 Å resolution range one should be careful with this as maps do not allow to obtain that information; if done for one catalytic water molecule, one could expect to see others also but that remains not that much reliable
- fractional TEMPyDiffMap: explain
- cooperativity known to occur: reference?
- NMA section: rather lengthy part; also, how reliable is that discussion? Model is based on model etc.
- interdomain cooperativity: could be discussed directly in the context of the structure description;
- "hydrophobic interactions": is incorrect / imprecise: such interactions are very weak (~10x less energy than a hydrogen bond); hence these should better be called hydrophobic contacts (several occurrences)
- Y465 interaction with F461: sounds like a pi-stacking interaction if reasonably parallel? Certainly not a "hydrophobic interaction"
- K489: is actually a stacking interaction with W257; flipped out compared to what? Other conformation seen in other states or only in crystal structure?
- H361 and K489: do mutants exist to address the function?
- "initial model" is incorrect term, meant is initial reconstruction; models are those for atomic models or in the AI part of Topaz (AI models) for example
- figure EV1: the 2 maps at the bottom left look identical, is that correct?
- Fig. 5: H963 fit and others not optimal; water molecule not really assignable at that resolution
- cryo-EM maps, half maps, masks etc. and fully refined atomic models should be deposited in the EMDB and PDB, and

representative data deposited onto the EMPIAR data base

Referee #1

The authors determined glycosylation and structures of both monomeric and dimeric soluble ACEs using cryo-EM technique which has several advantages compared to conventional X-ray crystallography including structural analysis of functional and glycosylated proteins. The paper is well written, comprehensive and well illustrated with detailed molecular models and movies to demonstrate the possible conformational alterations. The results are explained in detail and extensively documented. The results indicate that both domains of ACE are open conformation, monomeric ACE is more reminiscent of an hourglass shape, separated domains are closely associated and separated, a bridge formed between glycans in ACE homodimer, and alteration of active site upon dimerization. The study provides conclusive structural evidence that sACE dimerizes via the N-domain interface in the native state. These outcomes provide a better understanding of the dynamics of sACE activity and may help facilitate development of novel ACE inhibitors such as domain-specific inhibitors as discussed in the manuscript.

Thank-you for the encouraging remarks.

However, there are several concerns that should be appropriately addressed.

Major:
1. Since several previous studies used CHO-derived ACEs for molecular analyses, this study also employed the cell line to generate target ACE proteins. As mentioned in the Discussion section, ACEs can be glycosylated in a tissue-specific manner and the functional relevance remains unclear.

Thus, analysis of glycosylation should be performed using ACEs generated by other cell lines and compared to the present data.

There is unfortunately insufficient data available in the literature regarding the glycan structures of sACE expressed in different cell lines to perform a comparative analysis in the present study. No detailed glycan mass spectrometry studies have been reported for sACE to date. Although Kryukova *et al.* (2015) attempted to determine the differences in human lung vs seminal fluid sACE glycosylation, there was incomplete coverage of the protein sequence following trypsin cleavage and the peptides were not analysed by tandem mass spectrometry. The glycan structures proposed were thus putative and the results incomplete.

Glycosylation is known to be important for the processing and thermal stability of sACE (O'Neill *et al.*, 2008; Redelinghuys PhD). The importance of tissue-specific glycosylation, however, is not known at present. Tissue specific differences in glycosylation are unlikely to influence the enzyme's catalytic activity as deglycosylation with endoglycosidase H did not alter the kinetics of substrate hydrolysis (Yu *et al.*, 1997). It is possible that the interaction of sACE with other molecules like bilirubin, lysozyme, albumin, angiotensin receptor, or the bradykinin receptor is sensitive to tissue-specific differences in glycosylation, but this remains to be studied.

In the present study, we built the fucosylated complex-type glycan (the parts we could observe in the density maps) as it has been observed for the truncated ACE C-domain expressed in CHO-K1 cells (Yu *et al.*, 1997). We agree that it is important to study sACE glycosylation and believe that this is an exciting avenue for future work. However, with the limited information available, it is not currently possible to predict the functional relevance of tissue-specific sACE glycosylation.

2. Due to technical limitations of structural analysis, only soluble ACEs were used in this study.

Accordingly, possible structural differences between membrane ACE and soluble ACEs should be discussed.

Thank-you for pointing out this omission, we have now added a short discussion to address this point (Discussion page 18 paragraph 1).

The dimer structure solved in the present study agrees with the previous findings from studies on membrane-bound sACE dimerization where FRET, BiFC (Abrie *et al.*, 2018), cross-linking western blotting, and shedding assays with mutagenesis (Gordon *et al.*) were used. We have added citations for literature (Discussion page 23 paragraphs 1-2) and a statement that no large-scale structural differences are anticipated between soluble and membrane-bound sACE (Discussion page 18 paragraph 1).

Jaspard *et al.* (1995) assayed monolayers of cells with substrate and compared the kinetics of hydrolysis and chloride activation of sACE to the soluble form of the enzyme. Wild-type soluble and membrane-bound sACE in cell culture had the same K_m and k_{cat} values for hydrolysis of Ang I. In addition, the chloride activation of sACE was seen for both soluble sACE and membrane-bound sACE in cell culture (Jaspard *et al.*, 1995). Lanzillo *et al.* (1980) found no difference in the activity of sACE purified from different human tissue or fluid types (membrane sACE from kidney or lung homogenate, soluble sACE from seminal or blood plasma). Similarly, Wei *et al.* (1991) found no difference between soluble or membrane sACE activity after purification from CHO-K1 cells.

Likewise, describe pathophysiological significance of soluble ACEs and their inhibitions compared to membrane ACE.

We believe that the study of soluble sACE inhibition is physiologically relevant since no large-scale structural or functional differences are currently known between the soluble and membrane-bound forms of the enzyme (as discussed in the previous point). For instance, reports in the literature have shown that sACE inhibition *in vitro* (soluble sACE) translates to the *in vivo* (membrane-bound sACE) setting. Azizi *et al.* (2000) compared the *in vitro* and *in vivo* inhibition of the two active sites of sACE using 3 different substrates and 2 different ACE inhibitors and found that the domain-selective behaviour of the inhibitors/substrates and the efficacy of inhibition was comparable between the two forms.

3. Activity of ACEs obtained in this study was determined (49,094 {plus minus} 5,464 mU/mg) because cryo-EM allows determining structures of functional and active proteins. However, information regarding whether the result is reasonable and expected activity is not provided in this manuscript. Therefore, it is difficult to evaluate if this study determined structures of functional ACEs exhibiting expected activity.

We have added a literature citation (Lanzillo *et al.*, 1980) where 40U/mg was detected, in agreement with our result (Results page 6 paragraph 2).

Minor:
4. Generated ACEs were separated and characterized by a gel-chromatography and electrophoresis (Fig. 2). Would it have been more specific to identify monomeric ACEs by N- and C-domain-specific antibodies?

We agree that N- and C-domain specific antibodies are useful for identifying individual domains to confirm the integrity of the protein. In our case, we could establish that at least the

majority of protein was intact and any traces of single domains below the detection limit of the chromatogram would have been excluded by SEC. In response to this comment, we have tried to clarify the sections describing these results and methods (Results page 6 paragraph 2; Methods page 26 paragraph 1).

Referee #2:

The manuscript of Lubbe et al. is the summation of a huge amount of outstanding work. The authors are to be congratulated. There is no question that the observations in this manuscript are remarkable and outstanding and deserve to be published in EMBO.

Thank-you very much.

That said, the presentation of the data in the manuscript is not optimal. First, I am sure that Lubbe et al. must realize that virtually all readers of EMBO (and essentially all readers on-line) are not expert in structural analysis and I have my doubts as to exactly how many readers will remain engaged to the end of this long manuscript (my left hand has 5 fingers). Thus, I believe it absolutely incumbent upon the authors to shorten and simplify their presentation of the data. In fact, it is to the authors best interest to make this wonderful study intellectually accessible to more readers. Several suggests are below:

Introduction

1. The introduction is way too long.

The introduction of ACE in the introduction can be summarized in many fewer words.

We have removed details that do not directly pertain to the study, and which have previously been reported in other articles and reviews. We have condensed sentences where possible and shortened the introduction while still providing context for the study.

2. Despite the long introduction, the authors do not give any real introduction to cryo electron microscopy. What is it exactly in simple terms? How can it answer questions in a complementary fashion to crystallography.

We have added a reference in the introduction to a review which describes the principles of cryo-EM (Orlova & Saibil, 2011) (Introduction page 5 paragraph 1). We have also highlighted that cryo-EM, in contrast to X-ray crystallography, allows visualization of the protein under close-to-physiological conditions.

3. It would be very helpful to the reader to summarize clearly (as if you were explaining to a 1st year graduate student) what are the 3 or 4 most important questions you will investigate and answer in your study.

We agree with the reviewer. We have summarized the three key aims of our study and added them to the introduction (Introduction page 4 paragraph 4).

Results

4. Also too long. In some ways, this reads like a Ph.D. thesis. I would strongly urge the authors to focus on what they view as the most important questions and explain why they are the most important questions.

We have shortened the results section by shortening sentences and removing speculation so that the results are more focused. Points of discussion were moved to the discussion section. The section on dynamics (3DVA/NMA; Results page 11 paragraph 2) was also significantly shortened as suggested by reviewer 3. We believe that the revised introduction more clearly states the key questions and these questions are now also briefly given at the start of each results section so that it is more focused.

5. I would recommend the authors use several more subheaders ('cryo electron microscopy' is the first subheader as example) but use the subheaders to make simple declarative statements and summarize what they find in that part of the results (just as an example: 'assembly of x images shows ....' or something like 'both domains of ACE are closely associated'). The authors can do it better than I can but use these liberally to take the hand of the reader and explain the key conclusions of the data.

We agree that subheaders with declarative statements are very useful to guide the readers and have added them to the results section as suggested.

6. There are parts of the second half of the results where it is not clear what exactly is fact (based on the data) and what is speculation that may or may not be true. The section after 'Voronov et al. found...' is like that.

Another example is:

"The proximity of the N480 glycan could likely contribute to a change in the Cloop-1 dynamics and loss of the E596-R245 interaction could further destabilize Cloop-3. Disorder of the dimer C-domains may also cause a loss of R240 and Y241 hydrogen bonds to the C domain lid N681, which would contribute to destabilization of Cloop-1. C-domain disorder could also destabilize the Y607-E161-R479 interactions."

While all this may eventually be found to be true, in the context of this long study - and to make things easier for the reader - I would pare much of this speculation (could likely, may also, could also) from the Results and keep the Results simpler, focusing on what you find to be true. Even better would be to pose relatively simple (ie graduate student level) specific questions, then give data, then summarize conclusions.

We have revised the results section significantly by removing the speculations to be focused on facts from the present study.

Instead of the speculations regarding the hydroxyl radicals to the C^{loop}-3 link and activity, we have now performed additional *in silico* experiments (explained in the revised methods) and added their results to the figures (Fig EV5) and text (Results page 13 paragraphs 2-3 and page 14 paragraphs 1-2).

The CorrSite2.0 module of CavityPlus was used to predict allosteric sites for monomeric sACE based on motion correlation analyses of the detected protein cavities. Ma *et al.* (2016) found that the motions of orthosteric and allosteric sites are highly correlated. Based on this observation, CorrSite was developed to predict allosteric sites. It evaluates the motion correlation between protein cavities and how pseudoligand binding to a distal site affects the orthosteric/active site. Allosteric sites are identified as those where pseudoligand binding significantly affects the orthosteric site. Recently, CorrSite2.0 was developed which is superior to CorrSite since it was found to predict known allosteric sites with >90% accuracy, thus also outperforming previous allosteric site prediction servers (Xie *et al.* 2022).

We therefore chose CorrSite2.0 to predict allosteric sites for sACE and believe that this provides valuable insight into previous experimentally observed phenomena regarding cooperativity and allosteric inhibitory effects, and ties in with the 1,8-ANS docking and flexibility analyses performed in the present study. The implications of these results have also been added to the discussion of the revised manuscript (Discussion page 20 paragraph 3).

7. As a reader, I would urge a slightly greater focus on insight into catalytic activity and whether these data lead to general conclusions about enzymes similar to ACE.

Thank-you for this comment, we have now placed more emphasis on the effects of: large scale structural dynamics, hinging of the catalytic pocket and allostery on the catalytic activity of sACE. We have also introduced ACE2 earlier in the manuscript (Introduction page 3 paragraph 2), and mentioned enzymes with similar intradomain hinging mechanisms to sACE in the discussion (Discussion page 21 paragraph 2).

Concerning one section:

"Although the density is not of sufficient resolution to identify the potential ligand, the open N-domain active site is clearly primed for substrate binding to the zinc site and these 3 catalytic anchor residues. Although the domains are closely associated, full-length sACES1211 does not have a continuous active site channel for substrate cleavage. The N-domain active site cleft is instead inclined by ~30 degrees relative to the C-domain. This means that substrate molecules are unlikely to get cleaved by the one domain and then passed to the next. Endopeptidase activity at multiple sites has been observed for sACE with large substrates such as amyloid-β Larmuth et al., 2016; Oba et al., 2005; Zou et al., 2009). However, it is clear from this domain orientation that such substrates are unlikely to bind simultaneously to both domains to yield this activity. Yet, despite this independent active site arrangement, cooperativity is known to occur between the domains."

I do not understand how you conclude that substrates are unlikely to go from one site to the next site based on only a 30 degree incline. I would think that the probability of going from one site to the other site of a single enzyme is still far higher (given local concentration analysis) than the probability of going to a different site of a different ACE molecule.

We agree with the reviewer that this statement regarding the angle impeding passing of substrate from one active site to the next is incorrect. We have therefore removed this statement from the manuscript.

Also, your rationale for concluding that a single larger peptide may not bind simultaneously to both domains is also unclear (though perhaps true). Please make this section - which is very important - clearer.

We have attempted to clarify the original statement. Our interpretation of the sACE structural evidence presented here is that the endopeptidase activity observed for some substrates (like LH-RH, Amyloid beta, substance P) is unlikely to be due to simultaneous binding of these larger substrate molecules to both active sites by simply docking into the exposed active site clefts.

Our reasoning behind this is that: 1) The 45-degree incline (not 30-degrees as originally stated; corrected accordingly in the revised manuscript: Results page 10 paragraph 2; Figure 5 legend) between the two active site clefts increases the distance from the N-domain to C-domain zinc binding sites (where the scissile peptide bond would bind) so that substrates of insufficient length (like the decapeptide LH-RH or the undecapeptide substance P) would not be able to reach both sites simultaneously; 2) The location of the C-domain lid region could sterically hinder the substrate's binding from the N- to C-domain. If the N-terminus of a larger peptide like amyloid-beta was to bind to the N-domain, the longer peptide C-terminus could exit the N-domain prime subsite (right side of the N-domain on the two-domain structure in Figure 5) but binding to the C-domain via its exposed active site cleft would be sterically hindered by the presence of the C-domain lid region (dark purple C-domain helices Figure 5). The only likely access of such a peptide to the C-domain would be by threading from the N-domain through the very small pore underneath the C-domain lid region. We have changed our original statement to one that clarifies it as explained here (Results page 10 paragraph 2).

Discussion

8. I thought the discussion was good.

Movies

9. Very nice. It is probably not possible but it would be great to add a voice track explaining in a very few words what is happening.

We thank the reviewer for the appreciation of our work. We have included a movie legend for each movie which was mentioned in the results section text.

Referee #3:

The manuscript by Lubbe et al. addresses the structural organization of angiotensin-I converting enzyme (ACE) as determined by cryo-EM, image processing and significant structure sorting and focused refinements. This provides novel insights considering that this protein has until now only been studied at the level of the individual N- and C-terminal domains (crystal structures). The study now comprises the almost complete sequence (1211 residues out of 1277, the very C-terminus comprises a transmembrane region which hampers protein solubility). Furthermore, an important aspect is the presence of numerous protein modifications (glycans) that were not present in previous structures. The present work describes the overall architecture of the multi-domain protein, localizes glycan sites and gives insights into substrate-binding pockets (though no substrates are present). As the protein can form dimers or monomers (separated by image processing), the inter-domain protein-protein

interactions can be addressed, in particular for the N-terminal domain pair that is also the best resolved region and is shown to induce dimerization. Interesting is also that an interdomain linker in fact wraps around a domain instead of serving as a distance-creating linker.
Altogether, this work appears to be an interesting and important step forward in understanding the structure and function of ACE. The cryo-EM part appears sound technically and involved a number of specific approaches including sorting and focused refinements.

We thank the reviewer for the appreciation of our work.

The resolution is fine to address the overall structure and refine the atomic model (considering that crystal structures of parts exist) but one should be more careful with regards to side-chains positions/conformations and avoid over-interpretation (e.g. hydrogen-bonding between residues whose interpretation depend on precise side-chain conformations or a water molecule in a 3.7 Å map cannot be addressed). That being said, the structure as it is represents an important finding by itself without going too far in interpretation.

We have re-evaluated all interactions described in the manuscript and have revised the manuscript text and figures (Figures 7 and 8) to only include those interactions for which clear sidechain density was seen.

The part on the normal mode analysis etc. is less strong, probably also because some parts are a bit lengthy and could be described more concisely. I would suggest keeping the focus on the new structure which is the main new finding without going too much into hypothesis-driven NMA, flexibility etc.

We have revised the manuscript as explained in response to Reviewers 1 and 2 (which included shortening of the NMA section) to clarify our main questions and findings.

A question the reader may have from the beginning is in how far this is similar to the ACE2 receptor which is involved in SARS-CoV spike recognition; the answer to that comes at the end but could be introduced at the beginning.

Thank-you, we have added this comparison to the introduction (Introduction page 3 paragraph 2).

Detailed points:

1. clarify: N-glycosylation: was that analysed by MS? Do data exist that could be discussed in the context of the structure?

We did not analyse the *N*-glycosylation by mass spectrometry in the present study. The somatic (two-domain) form of sACE expressed in CHO-K1 cells (used in this study) has never been studied by mass spectrometry. As discussed in the manuscript (Results page 8 paragraph 3), the only glycan mass spectrometry data available for ACE expressed in CHO-K1 cells are of the truncated N- or C-domain (Yu *et al.,* 1997 and Anthony *et al.*, 2010). The presence of a second domain in the full-length sACE protein may slightly alter glycan site occupancy or the extent of

glycosylation at a particular site (compared to the truncated domains) due to steric hindrance, but this has not been studied before.

Glycosylation differs between cell types thus, although some data is available for glycosylation of human kidney sACE (Ehlers *et al.* 1992), human lung plasma sACE, or human seminal fluid sACE (Kryukova *et al.*, 2015), this cannot inform on the type of glycosylation for CHO-K1-expressed sACE in the present study. Furthermore, these studies did not provide detailed mass spectrometric analysis of the glycan structures and in some cases, sequence coverage following proteolytic cleavage by trypsin was incomplete (also discussed in our reply to Reviewer 1, comment 1). Our revised discussion mentions that comprehensive mass spectrometry analysis should be performed in future (Discussion page 24 paragraph 1)

2. full-length: almost (page 7)
We agree and have edited the manuscript text and figure legends accordingly to rather refer to the protein as full-length <u>soluble</u> sACE (the secretase/sheddase cleavage site is at S1203 and our construct includes 1-1211 of which 1-1201 was modelled).

3. 7 Å reconstruction: obtained how (page 7)
We agree that this should be stated. The method for obtaining the 7 Å reconstruction was described in detail in the Materials and Methods section. We have now added a brief description of the method to the results section, with reference to Figure EV1 for more detail (Results page 6 paragraph 3).

4. page 8: what is the meaning of the "truncated single domain forms" found in the classification?
These are the reconstructions shown on Figure EV3 which only showed density for a single domain plus a short stump around the interdomain linker region. They were indicated by a grey asterisk along with the other suboptimal particles (maps which could not be refined further) but we have now edited Figure EV3 to be clearer. Two red asterisks refer to junk particles like micrograph edges while the suboptimal particles have a single grey asterisk. We have added italicized letters to each 3D class from heterogeneous refinement and explained them in the text of the methods (Methods page 31 paragraph 2) and results (Results page 7 paragraph 2) sections.

5. page 8: "attracting" this is well known and doesn't refer to the 2021 paper cited here but instead to correlation artefacts known in image processing for more than 20 years; this is reference bias due to correlation artefacts; no need for this new naming
We have removed this and adjusted the sentence accordingly (Results page 7 paragraph 2).

6. page 8/9 local refinement, focused 3D classifications etc. and elsewhere in the MS and methods: as the structure sorting and refinements heavily rely on focused classifications and refinements it may be worth mentioning some key references such as Curr Op Struct Biol 2017, eLife 2018, Biosci Rep 2018 etc.
We agree and have added key references for local refinements, 3D variability analysis, and

focused 3D classifications to the appropriate results and methods sections (eg. Results page 7 paragraph 3).

7. references missing for Relion, Isolde, Phenix etc.
We have added the citations to each mention of the software used.

8. sphericity values: defined how?
We mentioned this in the methods but not in the results. It was determined by the 3D FSC server. We have now added this name to the results section as well and gave the appropriate reference (Results page 8 paragraph 1).

9. dimer processing: good that no C2 symmetry was imposed; yet, afterwards C2 symmetry expansion is mentioned, was it finally imposed or not or fully relaxed for final reconstructions?
We did not enforce C2 symmetry for the final reconstructions. The subtracted particles were first refined in C1 via non-uniform refine, then the resulting map was aligned to the C2 symmetry axis, used as a reference for a second non-uniform refine in C1, and the refined particles underwent C2 symmetry expansion. This was followed by local refinement of the expanded particle stack in C1. We have ensured that it is clear in the methods as well as the results sections (we clarified Figure EV3) that no symmetry was enforced.

10. particle subtraction etc.: with re-centering? that could improve image processing even further
The particles were not re-centered after particle subtraction. The subtracted particles of the dimer had a centre of mass of x, y, z = -0.053, -0.109, -12.74 pixels which is only ~14 Angstroms away from the center of the box. We thus believe that re-centering is unlikely to improve the resolution. Nevertheless, we have now tested this by re-centering the particles after subtraction and refining them as before. This did not improve the results over those reported in the manuscript.

11. "classes" should be better called class averages
We have adjusted the manuscript accordingly.

12. expected pattern of glycosylation: determined how?
This was based on the previous mass spectrometry of the truncated domains in CHO-K1 cells (as discussed in the previous comment 1 on *N*-glycosylation) and the sequence of sACE. Although the two domains are highly conserved, the glycan locations are unique and could thus be used as markers on the surface of the molecule to identify the domains at a low volume threshold prior to model building. We have now edited the sentence to clarify this (Results page 8 paragraph 2).

13. final resolution of 3.6 Å, more data needed to go further? Even a 3.3-3.4 Å map would make a significant difference for atomic model building, side chain positioning etc. (which looks approximate in some places as visible in the figures)

While we agree that higher resolution will improve model building, and collection of another dataset may in theory improve resolution by increasing the size of the particle stack (and thus the signal-to-noise ratio), we suspect that for this particular sample, the continuous and discrete heterogeneity of the apo protein would still be an important limiting factor to achieving high resolution. The bending at the interdomain linker of the monomer requires focused refinement of a single domain which has a small size and given the hinging between subdomains, it is likely difficult to align by the current algorithms. A larger dataset may not necessarily overcome this limit to resolution.

14. presence of proline next to Asn: if this hinders glycosylation then such places should probably not be called "sites"; instead only those really modified and confirmed by MS. E.g. N1196 and N494 appear not to be sites

We agree and have adjusted the manuscript accordingly to only refer to the 9 N-domain and 5 C-domain asparagines as sites (Introduction page 3 paragraph 3).

15. Diffmaps, explain

Diffmaps were calculated by Privateer between the experimental cryo-EM maps and the fitted models. Where density existed in a map which was not yet represented by the model (difference density), it indicated that model building was incomplete. Where these difference densities were strong, located around the *N*-linked glycosylation sites, and representative of a sugar molecule, it indicated that a sugar molecule could be added at that position. After glycan addition, the model was again validated by Privateer and checked for difference density and conformation and iterated until no more changes were required.

16. N131, N1162: partial density; could these be partially modified? Anything known from MS?

The density for the loop on which these glycosylation sites are found is poorer than the rest of the structure. It is also poorly resolved in several high-resolution crystal structures (where site N131 or N1162 was mutated). Our maps showed no evidence for glycosylation at these sites, but it could just be due to the low resolution or high flexibility in this region. We don't have conclusive evidence that the two sites are not glycosylated in sACE expressed in CHO-K1 cells, or whether they are partially modified. Site N131 was glycosylated in the truncated N-domain based on mass spectrometry after PNGaseF deamidation (Anthony *et al.* 2010). Mass spectrometry of the truncated C-domain showed that the N1162 site was present in both glycosylated and non-glycosylated forms (Yu *et al.*, 1997). The presence of a disulfide bond may reduce the level of glycosylation at this site.

17. ACE2: what is the sequence identity and similarity? "homology" is not the correct term here. Topic to be introduced earlier

We have introduced ACE2 earlier (Introduction page 3 paragraph 2) and provided the sequence identity and similarity in the results section (Results page 10 paragraph 1).

18. activation of a water molecule: based on which knowledge? Any paper on this?
This is based on QM/MM simulations and the presence of a water molecule coordinated to the active site zinc ion in a published crystal structure of the ACE N-domain (PDB ID: 6ZPQ). This structure was used as a template for model-building of the N-domain in the present study. We have added the citation for 3 papers on QM/MM analysis of the ACE domains (Wang *et al.*, 2011; Zhang *et al.*, 2013; Mu *et al.*, 2016) (Results page 11 paragraph 1).

19. visualisation of a water molecule: in the 3.5 - 4.5 Å resolution range one should be careful with this as maps do not allow to obtain that information; if done for one catalytic water molecule, one could expect to see others also but that remains not that much reliable
The value of 3.7 Å is the global resolution across the N-domain map which contains the disordered glycans as well as the better resolved protein core. While we agree that a map of such resolution cannot provide conclusive evidence for the absence/presence of a water molecule, the features of the cryo-EM map at the N-domain zinc-binding site agreed very well with the high-resolution crystal structure (PDB ID: 6ZPQ) (where the zinc was clearly coordinated by a water molecule and two histidine sidechains) and the cryo-EM density seemed to support placement of a water molecule. The crystal structure H361 and H365 sidechain orientations were observed in the monomer N-domain cryo-EM structure, and we thus maintained the crystal structure coordination geometry, including the active site water molecule, during model building. Presence of the water molecule is further supported by QM/MM studies on ACE where the water was shown to play an important role in catalysis (as explained in the previous point). Nevertheless, we have removed the water molecule indicated previously for the N-domain on Figure 5 as the evidence for its placement did not come directly from the results of the present study. We now only refer to the zinc and coordinating histidine sidechains of the cryo-EM structures in the results section (Results page 11 paragraph 1). We did not model a zinc ion for the C-domain active site as the resolution was generally poorer and we saw no density for a zinc ion.

20. fractional TEMPyDiffMap: explain
The TEMPy:DiffMap (Joseph *et al.* 2020) tool of CCPEM was used to calculate differences between the final atomic model and the cryo-EM map of sACE. Fractional difference maps were calculated with local scaling and pre-processing (dust filtering and masking) since this takes into account local resolution variations and has been reported to allow identification of clean and interpretable differences of conformation and composition (like ligands) in cryo-EM maps (Joseph *et al.* 2020)

We have made this clearer in the method section (Methods page 36 paragraph 2) and referred to these as: "fractional difference maps" in the results (Results page 11 paragraph 1) and figure legend. We have also added the Joseph *et al.* (2020) reference.

21. cooperativity known to occur: reference?
We have added these references (Results page 11 paragraph 2).

22. NMA section: rather lengthy part; also, how reliable is that discussion? Model is based on model etc.
We have significantly shortened this section.

We believe that the NMA section is relevant since 1) the intradomain interactions of the truncated domains observed by NMA agree with previous MD simulations, crystal structures, and the 3DVA results presented in this manuscript; 2) the interdomain interactions of the two-domain monomer observed by NMA agree with the 3DVA results presented here. We agree that there is uncertainty in the full-length dimer model as it is based on restrained domain fitting into a frame of the 3DVA component. The NMA motions observed for this model do, however, agree very well with the experimentally observed domain motions observed by 3DVA in the present study. The orientation of the C-domains in the full-length dimer model presented here is also supported by published research describing 1) the involvement of the C-domain free cysteine in dimerization; 2) that C-domain glycans are not important for dimerization; 3) that dimerization decreases shedding from the cell membrane (Discussion page 23 paragraph 2).

23. interdomain cooperativity: could be discussed directly in the context of the structure description;
Thank-you for the suggestion, after editing the manuscript overall to make the results clearer we believe this change is no longer necessary. We have first described the static conformation of the modelled structure with its active site conformation in comparison to the crystal structures and then go on to describing dynamic changes and cooperativity.

24. "hydrophobic interactions": is incorrect / imprecise: such interactions are very weak (~10x less energy than a hydrogen bond); hence these should better be called hydrophobic contacts (several occurrences)
We have changed all instances of "hydrophobic interactions" to hydrophobic contacts.

25. Y465 interaction with F461: sounds like a pi-stacking interaction if reasonably parallel? Certainly not a "hydrophobic interaction"
We thank the reviewer for noticing this. We have checked this interaction and adjusted the sentence and figures accordingly (Results page 15 paragraph 3).

26. K489: is actually a stacking interaction with W257; flipped out compared to what? Other conformation seen in other states or only in crystal structure?
We agree and have edited this sentence to make it clearer (Discussion page 23 paragraph 2). The K489 sidechain is flipped out in the dimer compared to the monomer cryo-EM N-domain. The dimer's orientation also differs from the crystal structures.

27. H361 and K489: do mutants exist to address the function?
We have a double mutant of sACE-H361K, H365K which was previously used to create an N-domain knockout protein (Wei *et al.,* 1992). Mutation of both residues thus abolishes activity of the N-domain while not affecting catalysis by the C-domain. We do not, however, have a single mutant of H361 at present and the effect of its mutation is thus not known. It is likely, however,

that it would have reduced activity as it is important for zinc coordination and thus substrate cleavage.

A mutant for the C-domain equivalent of K489 (K1087) was previously reported in literature (Naqvi *et al.,* 2005) but there is no mutant of K489 itself. The K1087A C-domain mutant (in a truncated single-domain protein) showed drastically reduced catalytic activity for 5 substrates and given the homology between the two domains and evolutionary conservation of K489 revealed in the present study, it is likely that mutation of K489 would also drastically reduce N-domain activity.

28. "initial model" is incorrect term, meant is initial reconstruction; models are those for atomic models or in the AI part of Topaz (AI models) for example
We agree and have changed it to "initial reconstruction" (Methods page 28 paragraph 1 and Figure EV1).

29. figure EV1: the 2 maps at the bottom left look identical, is that correct?
We have confirmed that the figure is correct. We have edited the method section to mention that refinement of this subset of particles gave a low-resolution map similar to the initial 3D reconstruction (Methods page 28 paragraph 1).

30. Fig. 5: H963 fit and others not optimal; water molecule not really assignable at that resolution
We agree that the C-domain is of lower resolution and the sidechain densities (eg. H963) are not entirely clear. For that reason, the zinc-coordinating sidechains were restrained during model building to their orientations in the template crystal structure. The comment regarding the water molecule was addressed for the N-domain in the previous comment 19. We did not model a water molecule or zinc ion in the C-domain.

31. cryo-EM maps, half maps, masks etc. and fully refined atomic models should be deposited in the EMDB and PDB, and representative data deposited onto the EMPIAR data base

The maps, half-maps, masks, and fully refined atomic models are deposited in the EMDB and the PDB and listed in the data availability section. We have now also deposited the raw micrographs onto the EMPIAR database and have added the accession code to the data availability section (page 38). The wwPDB validation reports for our data deposited onto the PDB and EMDB were uploaded as related manuscript files, and our EMPIAR entry can be accessed using the token provided in the letter to the editor.

1st Revision - Editorial Decision 27th May 2022

Dear Lizelle,

Congratulations on an excellent manuscript, I'm pleased to inform you that your manuscript has been accepted for publication in The EMBO Journal. Thank you for your comprehensive response to the referee concerns.

I will begin the final checks on your manuscript before submitting to the publisher next week. Once at the publisher, it will take about 3 weeks for your manuscript to be published online. As a reminder, the entire review process including referee concerns and your point-by-point response will be available to readers.

It has been a pleasure to work with you to get this to the acceptance stage. I will be in touch throughout the final editorial process until publication. In the meantime, I hope you find time to celebrate!

Kind regards,

Kelly

Kelly M Anderson, PhD
Editor
The EMBO Journal
k.anderson@embojournal.org

Referee #1:

We have reviewed the responses by the authors and have examined the revised segments of the manuscript.
We are impressed by the detailed comments and the revisions that the authors have made. They responded adequately to all comments and suggestions and improved the manuscript. We have no further suggestions.

Please note that it is EMBO Journal policy for the transcript of the editorial process (containing referee reports and your response letter) to be published as an online supplement to each paper. If you do NOT want this, you will need to inform the Editorial Office via email immediately. More information is available here:
https://www.embopress.org/page/journal/14602075/authorguide#transparentprocess

Your manuscript will be processed for publication in the journal by EMBO Press. Manuscripts in the PDF and electronic editions of The EMBO Journal will be copy edited, and you will be provided with page proofs prior to publication. Please note that supplementary information is not included in the proofs.

Please note that you will be contacted by Wiley Author Services to complete licensing and payment information. The required 'Page Charges Authorization Form' is available here: https://www.embopress.org/pb-assets/embo-site/tej_apc.pdf

Should you be planning a Press Release on your article, please get in contact with embojournal@wiley.com as early as possible, in order to coordinate publication and release dates.

If you have any questions, please do not hesitate to call or email the Editorial Office. Thank you for your contribution to The EMBO Journal.

** Click here to be directed to your login page: https://emboj.msubmit.net

EMBO Press Author Checklist

Corresponding Author Name: Lizelle Lubbe
Journal Submitted to: EMBO J
Manuscript Number: EMBOJ-2021-110550

USEFUL LINKS FOR COMPLETING THIS FORM
The EMBO Journal - Author Guidelines
EMBO Reports - Author Guidelines
Molecular Systems Biology - Author Guidelines
EMBO Molecular Medicine - Author Guidelines

Reporting Checklist for Life Science Articles (updated January 2022)
This checklist is adapted from Materials Design Analysis Reporting (MDAR) Checklist for Authors. MDAR establishes a minimum set of requirements in transparent reporting in the life sciences (see Statement of Task: 10.31222/osf.io/9sm4x). Please follow the journal's guidelines in preparing your manuscript.
Please note that a copy of this checklist will be published alongside your article.

Abridged guidelines for figures
1. Data
The data shown in figures should satisfy the following conditions:
- the data were obtained and processed according to the field's best practice and are presented to reflect the results of the experiments in an accurate and unbiased manner.
- ideally, figure panels should include only measurements that are directly comparable to each other and obtained with the same assay.
- plots include clearly labeled error bars for independent experiments and sample sizes. Unless justified, error bars should not be shown for technical replicates.
- if n<5, the individual data points from each experiment should be plotted. Any statistical test employed should be justified.
- Source Data should be included to report the data underlying figures according to the guidelines set out in the authorship guidelines on Data Presentation.

2. Captions
Each figure caption should contain the following information, for each panel where they are relevant:
- a specification of the experimental system investigated (eg cell line, species name).
- the assay(s) and method(s) used to carry out the reported observations and measurements.
- an explicit mention of the biological and chemical entity(ies) that are being measured.
- an explicit mention of the biological and chemical entity(ies) that are altered/varied/perturbed in a controlled manner.
- the exact sample size (n) for each experimental group/condition, given as a number, not a range;
- a description of the sample collection allowing the reader to understand whether the samples represent technical or biological replicates (including how many animals, litters, cultures, etc.).
- a statement of how many times the experiment shown was independently replicated in the laboratory.
- definitions of statistical methods and measures:
  - common tests, such as t-test (please specify whether paired vs. unpaired), simple χ2 tests, Wilcoxon and Mann-Whitney tests, can be unambiguously identified by name only, but more complex techniques should be described in the methods section;
  - are tests one-sided or two-sided?
  - are there adjustments for multiple comparisons?
  - exact statistical test results, e.g., P values = x but not P values < x;
  - definition of 'center values' as median or average;
  - definition of error bars as s.d. or s.e.m.

Please complete ALL of the questions below.
Select "Not Applicable" only when the requested information is not relevant for your study.

Materials

Newly Created Materials	Information included in the manuscript?	In which section is the information available? (Reagents and Tools Table, Materials and Methods, Figures, Data Availability Section)
New materials and reagents need to be available; do any restrictions apply?	Yes	Materials and Methods section; materials will be made available upon reasonable request

Antibodies	Information included in the manuscript?	In which section is the information available? (Reagents and Tools Table, Materials and Methods, Figures, Data Availability Section)
For **antibodies** provide the following information:<br>- Commercial antibodies: RRID (if possible) or supplier name, catalogue number and or/clone number<br>- Non-commercial: RRID or citation	Not Applicable	NA

DNA and RNA sequences	Information included in the manuscript?	In which section is the information available? (Reagents and Tools Table, Materials and Methods, Figures, Data Availability Section)
**Short novel DNA or RNA including primers, probes**: provide the sequences.	Not Applicable	NA

Cell materials	Information included in the manuscript?	In which section is the information available? (Reagents and Tools Table, Materials and Methods, Figures, Data Availability Section)
**Cell lines:** Provide species information, strain. Provide accession number in repository **OR** supplier name, catalog number, clone number, and/**OR** RRID.	Yes	Materials and Methods section
**Primary cultures:** Provide species, strain, sex of origin, genetic modification status.	Not Applicable	NA
Report if the cell lines were recently **authenticated** (e.g., by STR profiling) and tested for mycoplasma contamination.	Not Applicable	not tested for mycoplasma contamination

Experimental animals	Information included in the manuscript?	In which section is the information available? (Reagents and Tools Table, Materials and Methods, Figures, Data Availability Section)
**Laboratory animals or Model organisms:** Provide species, strain, sex, age, genetic modification status. Provide accession number in repository **OR** supplier name, catalog number, clone number, **OR** RRID.	Not Applicable	NA
**Animal observed in or captured from the field:** Provide species, sex, and age where possible.	Not Applicable	NA
Please detail **housing and husbandry conditions**.	Not Applicable	NA

Plants and microbes	Information included in the manuscript?	In which section is the information available? (Reagents and Tools Table, Materials and Methods, Figures, Data Availability Section)
**Plants:** provide species and strain, ecotype and cultivar where relevant, unique accession number if available, and source (including location for collected wild specimens).	Not Applicable	NA
**Microbes:** provide species and strain, unique accession number if available, and source.	Not Applicable	NA

Human research participants	Information included in the manuscript?	In which section is the information available? (Reagents and Tools Table, Materials and Methods, Figures, Data Availability Section)
If collected and within the bounds of privacy constraints report on age, sex and gender or ethnicity for all study participants.	Not Applicable	NA

Core facilities	Information included in the manuscript?	In which section is the information available? (Reagents and Tools Table, Materials and Methods, Figures, Data Availability Section)
If your work benefited from core facilities, was their service mentioned in the acknowledgments section?	Yes	Acknowledgements

Design

Study protocol	Information included in the manuscript?	In which section is the information available? (Reagents and Tools Table, Materials and Methods, Figures, Data Availability Section)
If study protocol has been **pre-registered, provide DOI in the manuscript**. For clinical trials, provide the trial registration number **OR** cite DOI.	Not Applicable	NA
Report the **clinical trial registration number** (at ClinicalTrials.gov or equivalent), where applicable.	Not Applicable	NA

Laboratory protocol	Information included in the manuscript?	In which section is the information available? (Reagents and Tools Table, Materials and Methods, Figures, Data Availability Section)
Provide DOI OR other citation details if **external detailed step-by-step protocols** are available.	Not Applicable	NA

Experimental study design and statistics	Information included in the manuscript?	In which section is the information available? (Reagents and Tools Table, Materials and Methods, Figures, Data Availability Section)
Include a statement about **sample size** estimate even if no statistical methods were used.	Yes	All sample sizes (Cryo-EM protein particle numbers) were given in the Materials and Methods, Figures or Results sections
Were any steps taken to minimize the effects of subjective bias when allocating animals/samples to treatment (e.g. **randomization procedure**)? If yes, have they been described?	Not Applicable	NA
Include a statement about **blinding** even if no blinding was done.	Not Applicable	NA
Describe **inclusion/exclusion criteria** if samples or animals were excluded from the analysis. Were the criteria pre-established?<br><br>If sample or data points were omitted from analysis, report if this was due to attrition or intentional exclusion and provide justification.	Yes	Details were given in the Materials and Methods sections where cryo-EM protein particles were excluded from downstream image processing
For every figure, are **statistical tests** justified as appropriate? Do the data meet the assumptions of the tests (e.g., normal distribution)? Describe any methods used to assess it. Is there an estimate of variation within each group of data? Is the variance similar between the groups that are being statistically compared?	Not Applicable	NA

Sample definition and in-laboratory replication	Information included in the manuscript?	In which section is the information available? (Reagents and Tools Table, Materials and Methods, Figures, Data Availability Section)
In the figure legends: state number of times the experiment was **replicated** in laboratory.	Not Applicable	NA
In the figure legends: define whether data describe **technical or biological replicates**.	Not Applicable	NA

Ethics

Ethics	Information included in the manuscript?	In which section is the information available? (Reagents and Tools Table, Materials and Methods, Figures, Data Availability Section)
Studies involving **human participants**: State details of **authority granting ethics approval** (IRB or equivalent committee(s), provide reference number for approval.	Not Applicable	NA
Studies involving **human participants**: Include a statement confirming that **informed consent** was obtained from all subjects and that the experiments conformed to the principles set out in the WMA Declaration of Helsinki and the Department of Health and Human Services Belmont Report.	Not Applicable	NA
Studies involving **human participants:** For publication of **patient photos**, include a statement confirming that consent to publish was obtained.	Not Applicable	NA
Studies involving experimental **animals**: State details of **authority granting ethics approval** (IRB or equivalent committee(s), provide reference number for approval. Include a statement of compliance with ethical regulations.	Not Applicable	NA
Studies involving **specimen and field samples:** State if relevant **permits** obtained, provide details of authority approving study; if none were required, explain why.	Not Applicable	NA

Dual Use Research of Concern (DURC)	Information included in the manuscript?	In which section is the information available? (Reagents and Tools Table, Materials and Methods, Figures, Data Availability Section)
Could your study fall under dual use research restrictions? Please check biosecurity documents and list of **select agents and toxins** (CDC): https://www.selectagents.gov/sat/list.htm	Not Applicable	NA
If you used a select agent, is the security level of the lab appropriate and reported in the manuscript?	Not Applicable	NA
If a study is subject to dual use research of concern regulations, is the name of the **authority granting approval and reference number** for the regulatory approval provided in the manuscript?	Not Applicable	NA

Reporting

The MDAR framework recommends adoption of discipline-specific guidelines, established and endorsed through community initiatives. Journals have their own policy about requiring specific guidelines and recommendations to complement MDAR.

Adherence to community standards	Information included in the manuscript?	In which section is the information available? (Reagents and Tools Table, Materials and Methods, Figures, Data Availability Section)
State if relevant guidelines or checklists (e.g., **ICMJE, MIBBI, ARRIVE, PRISMA**) have been followed or provided.	Not Applicable	NA
For **tumor marker prognostic studies,** we recommend that you follow the **REMARK** reporting guidelines (see link list at top right). See author guidelines, under 'Reporting Guidelines'. Please confirm you have followed these guidelines.	Not Applicable	NA
For **phase II and III randomized controlled trials**, please refer to the **CONSORT** flow diagram (see link list at top right) and submit the CONSORT checklist (see link list at top right) with your submission. See author guidelines, under 'Reporting Guidelines'. Please confirm you have submitted this list.	Not Applicable	NA

Data Availability

Data availability	Information included in the manuscript?	In which section is the information available? (Reagents and Tools Table, Materials and Methods, Figures, Data Availability Section)
Have **primary datasets** been deposited according to the journal's guidelines (see 'Data Deposition' section) and the respective accession numbers provided in the Data Availability Section?	Yes	Accession codes for raw cryo-EM micrographs (EMPIAR database), cryo-EM maps (EMDB database), and atomic coordinates (PDB database) are provided in the "Data Availability" section
Were **human clinical and genomic datasets** deposited in a public access-controlled repository in accordance to ethical obligations to the patients and to the applicable consent agreement?	Not Applicable	NA
Are **computational models** that are central and integral to a study available without restrictions in a machine-readable form? Were the relevant accession numbers or links provided?	Not Applicable	NA
If publicly available data were reused, provide the respective **data citations in the reference list**.	Not Applicable	NA
